# Supplementary material for: A comparative field evaluation of six medicine quality screening devices in Laos
Source: PLoS Negl Trop Dis. 2021 Sep 30;15(9):e0009674. doi: 10.1371/journal.pntd.0009674 (PMC8483322; doi:10.1371/journal.pntd.0009674)
Supplement: S1 Text — (PDF) [file pntd.0009674.s001.pdf]

S1 Text. UPLC confirmatory methods protocols

Summary of changes made to adapt HPLC methods to UPLC

Overview

Pharmacopeial methods utilizing HPLC were adapted for UPLC primarily by using columns with smaller particle sizes and dimensions. This resulted in lower flow rates, smaller injection volumes, and significantly shortened cycle times while maintaining the required quality of separations. With the exception of sulfamethoxazole and trimethoprim, the C18 column chemistry specified in the pharmacopeial methods was used. Separations by UPLC provided the additional benefit of significant reductions in solvent use.

Pharmacopeial protocols called for isocratic elution for all active ingredients except artemether/lumefantrine. The UPLC methods used isocratic separations for all adapted pharmacopeial methods. Proportions of mobile phases A and B were modified to improve separations and reduce cycle times. Unless noted below, mobile phases and detection wavelengths were the same or modified slightly. When detection wavelengths were altered, two actives with different absorbance spectra were being analyzed (e.g. sulfamethoxazole and trimethoprim), and the changes improved measurements.

In most instances the solvents used for extractions were the same as used in the pharmacopeial methods [1]. When these were altered, it simplified the solvents while ensuring the solubility of the active ingredients. Whereas pharmacopeial methods often specify the extraction of multiple tablets, in this study samples were analyzed on a per tablet basis, often sampling a fraction of the ground tablet.

A pharmacopeial method was not available for dihydroartemisinin/piperaquine and an HPLC method from the literature was adapted as described below.

Comments on Specific Methods

The following provides references to pharmacopeial methods that formed the basis of the adapted methods and highlights adaptations of significance.

1. Amoxicillin and Clavulanate
   The analysis of Amoxicillin and Clavulanate was adapted from the USP Monographs, 2017, pp 2792-3. The NaH_2PO_4 buffer was reduced from 65mM to 10 mM.
2. Artemether and Lumefantrine
   The analysis of artemether and lumefantrine was adapted from The Int. Pharmacopoeia Sixth Ed., 2016. Artemether was dissolved using a simplified solvent containing KH_2PO_4 buffer and acetonitrile (50:50), in which lumefantrine is sparingly soluble. This enhanced the signal from artemether relative to lumefantrine. Lumefantrine was dissolved in dimethyl sulfoxide. Artemether and lumefantrine were measured from separate injections using different isocratic conditions. Detection of lumefantrine was changed to 300 nm.
3. Artesunate
   The analysis of artesunate followed the method in The Int. Pharmacopoeia Sixth Ed., 2016. The only changes made were those related to the column used for UPLC.
4. Azithromycin
   The analysis of azithromycin was adapted from the method published in 2010 in The United States Pharmacopeial Convention. The extraction solvent was changed to 30% 33 mM KH_2PO_4, pH 8.5, 70% methanol. Octanesulfonate was removed from the mobile phase.
5. Dihydroartemisinin and Piperaquine
   A pharmacopeial method for dihydroartemisinin and piperaquine was unavailable and a method was adapted from Peterson et.al., PLoS One. 2017, https://doi.org/10.1371/journal.pone.0184165 September 6, 2017 pp 1-22. Dihydroartemisinin was dissolved in acetonitrile and mobile phase A was 10 mM KH_2PO_4, pH 3.0. For Piperaquine analysis, a gradient was used in place of isocratic conditions and the detection wavelength was changed to 240 nm.
6. Ofloxacin
   The analysis of ofloxacin was adapted was adapted from the USP Monographs, 2017, pp 5413-4. Standards and extracts were prepared with water.

7. Sulfamethoxazole and Trimethoprim

   The analysis was adapted from the USP Monographs, 2017, pp 6272-3. A Waters BEH-phenyl column was used. Mobile phase A was water with 0.2% acetic acid. Detection wavelengths were optimized for sulfamethoxazole (270 nm) and trimethoprim (240 nm).

Standard and Sample Preparations

Amoxicillin/Clavulanic Acid

- Standards: Make a combined Amoxicillin and Clavulanate Standard
    1. Weigh 9 - 11 mg Potassium clavulanate into a 12 mL vial.
    2. Add 10 ml water and sonicate 5 min.
    3. Weigh 23 - 27 mg Amoxicillin trihydrate into a 25 ml volumetric flask
    4. Add 10 - 15 ml water and mix.
    5. Add 4.000 ml of the clavulanate stock and sonicate 5 min.
    6. Bring to volume and mix thoroughly.
    7. Dilute as needed to be in sample range.
- Sample Preparation
    1. Record tablet mass.
    2. Divide tablet into 4 quarters. Store in a vial at room temperature away from light.
    3. Weigh one of the tablet quarters, record mass and transfer to a flask
    4. Add 150 - 300 ml water (so that amoxicillin is approx. 0.8 mg/ml. Record the ml of water added.
    5. Sonicate for 5 min.
    6. Mix with stir bar for 15 - 30 min.
    7. Centrifuge 1.6 mL of sample in a microfuge at high speed for 4 min.
    8. Transfer 1 mL of clear sample to UPLC vial for analysis.
- Notes
    1. Tablet extractions have been stable for both amoxicillin and clavulanate for at least 24 hours.
    2. Clavulanate Standards may be used for up to 24 hr.
    3. Amoxicillin is stable for at least 2 days.

Artemether

- Standards
    - Artemether
        1. Dissolve 8 - 10 mg in 10 ml of 50% A, 50% B.
        2. Sonicate 5 min.
        3. Use as standard.

- Sample Preparation
    - Artemether
        1. Weigh out 1/6 to 1/5 of tablet weight into an 11 ml glass vial. Sonicate 5 min.
        2. Add 5 ml of 50% A, 50%B.
        3. Sonicate 5 min.
        4. Measure as soon as possible and no later than 5 hour after dissolving
    - Notes
        1. Protect API from light
        2. Tablet extractions for both Artemether and Lumefantrine have been stable

Artesunate

- Standard Preparation
    1. Weigh approx. 100 mg into a 25 ml volumetric flask. Record the mass.
    2. Fill flask approx. 90% with acetonitrile, sonicate 5 min, and bring to volume with acetonitrile.
    3. Dilute to obtain standards ranging from approx. 4 to 1 mg/ml.
- Sample Preparation
    1. Open top of vial and add 10 ml acetonitrile. Swirl to dissolve contents.
    2. Transfer to a 25 ml volumetric flask
    3. Repeat transfer from the vial to the volumetric flask with two 5 ml washes
    4. Sonicate 2 min and bring to volume.

Azithromycin

- Standard Preparation
    1. Dissolve 25 mg in 25 ml mobile phase
    2. Dilute 10-fold for approximately 100 µg/ml, or as needed for a calibration curve
- Sample Preparation
    1. Record weight of tablet
    2. Crush tablet into a fine powder using a mortar and pestle, store in 11 ml vial
    3. Transfer 1/10 to 1/5 of tablet to 25 ml of mobile phase
    4. Dilute 10-fold in mobile phase for approx. 100 µg/ml

Dihydroartemisinin

- Standard Preparation
    1. Weigh 9 - 11 mg dihydroartemisinin.
    2. Dissolve in 10 ml acetonitrile
    3. Sonicate 5 minutes.

4. Dilute 75%, 50% and 25% for a set of calibration standards

- Sample Preparation
  1. Record tablet mass.
  2. Grind to a fine powder.
  3. Weigh out approximately 1/10 of tablet mass to a 15 ml high speed centrifuge tube. Record mass.
  4. Add 5.00 ml acetonitrile.
  5. Sonicate for 5 min.
  6. Centrifuge 12,000 x G for 5 min.
  7. Measure extract without further dilution.
- Notes
  1. Standards stable for at least 1 days (at room temp).
  2. Tablet extractions stable for at least 24 hr.
  3. This method separates the alpha and beta epimers, combine peak areas from both peaks for quantitation.

Lumefantrine

- Standard Preparation
  1. Dissolve 10 -14 mg into 25 ml of dimethyl sulfoxide
  2. Sonicate 5 min.
  3. Dilute as needed in 50%water/50% acetonitrile to obtain a standard at 15 -20 µg/ml.
- Sample Preparation
  1. Weigh out 1/10 of tablet weight into a 25 ml volumetric flask.
  2. Add approx. 20 ml dimethyl sulfoxide.
  3. Sonicate 5 min and bring to volume with dimethyl sulfoxide.
  4. Combine 100 µl extract with 3.000 ml 35% 10 mM KH_2PO_4, pH 3.0/65% acetonitrile
- Notes
  1. Protect API from light
  2. Tablet extractions for both Artemether and Lumefantrine have been stable

Ofloxacin

- Standard Preparation
  1. Weigh 19 - 21 mg ofloxacin into a 40 ml extraction vial.
  2. Add 20 ml water using a 10 ml glass pipet.
  3. Sonicate 5-10 min or until the ofloxacin is completely dissolved.

4. Dilute 500 µl of the stock with 24.500 ml water using a 25 ml volumetric flask for a standard at approximately 20 µg/ml.

- Sample Preparation
  1. Record tablet mass.
  2. Cut tablet in half using a tablet cutter.
  3. Weigh one of the tablet halves and record mass.
  4. Place the half tablet into a 250 ml flask. Add 200 ml water - record volume of water.
  5. Sonicate 5 min or until the tablet is completely broken apart.
  6. Mix on stir plate for 15 min.
  7. Transfer 1.8 ml to a 2 ml microfuge tube and centrifuge at high speed for 4 min.
  8. Dilute 1000 µl of the spun extract with 24.000 ml water using a 25 ml volumetric flask.
- Notes
  1. Standards and extracts stable for at least 4 days (at room temperature).
  2. Do not filter through polyethersulfone

Piperaquine

- Standard Preparation
  1. Weigh 9 - 11 mg Piperaquine tetraphosphate tetrahydrate in to 50 ml volumetric flask.
  2. Add approximately 40 ml 0.01 N hydrochloric acid
  3. Sonicate 5 minutes.
  4. Bring to volume and mix thoroughly. Use as calibration standard (approx. 200 µg/ml).
- Sample Preparation
  1. Record tablet mass.
  2. Grind to a fine powder.
  3. Weigh out approximately 1/5 of tablet mass to a 250 ml flask. Record mass.
  4. Add 100 ml 0.01 N hydrochloric acid. Record the ml of water added. (piperaquine at approximately 640 µg/ml).
  5. Sonicate for 5 min.
  6. Mix with stir bar for 15 - 30 min.
  7. Centrifuge 1.6 ml of sample in a microfuge at high speed for 4 min.
  8. Add 1.00 ml water to UPLC vials. Add 500 µl of extract to vial and mix.
- Notes
  1. Standards stable for at least 4 days (at room temperature).
  2. Tablet extractions stable for at least 2 days.

Sulfamethoxazole/Trimethoprim

- Standard Preparation
    1. Weigh 28 - 32 mg Sulfamethoxazole into a 25 ml volumetric flask.
    2. Weigh 9 - 11 mg Trimethoprim into the same flask.
    3. Add 20 mL methanol and sonicate 5 min.
    4. Bring to volume
    5. Dilute as needed to be in sample range.
- Sample Preparation
    1. Record tablet mass.
    2. Grind tablet to a fine powder using a mortar/pestle. Store in a vial at room temperature away from light.
    3. Transfer 1/10 - 1/4 of tablet mass to a vial or volumetric flask.
    4. Record the mass to be extracted.
    5. Dissolve in 10, 20, or 25 ml methanol, depending on mg extracted.
    6. Sonicate 5 min.
    7. Centrifuge 4 ml of sample in at 13,000 x g for 4 min.
    8. Dilute 100 µl of clear sample with 5.00 ml methanol. Transfer to UPLC vial for analysis.
- Notes
    1. Protect API from light
    2. Stability of tablet extractions: At least 48 hr

Reference

1. United States Pharmacopeia and National Formulary (USP 40-NF 35). United States Pharmacopeial Convention; 2017. Available from: https://www.uspnf.com/official-text/proposal-statuscommentary/usp-40-nf-35
