# Supplementary material for: A comparative field evaluation of six medicine quality screening devices in Laos
Source: PLoS Negl Trop Dis. 2021 Sep 30;15(9):e0009674. doi: 10.1371/journal.pntd.0009674 (PMC8483322; doi:10.1371/journal.pntd.0009674)
Supplement: S2 Text — (PDF) [file pntd.0009674.s002.pdf]

S2 Text. Basic processes for qualitative spectral comparison and protocols for reference library creation

Text A. Illustration of the basic processes for qualitative spectral comparison 2

Text B. The MicroPHAZIR RX spectrometer 3

Text C. The 4500a FTIR spectrometer 4

Text D. The Progeny spectrometer 5

Text E. The Truscan RM spectrometer 6

Text F. Difficulties encountered during reference library entries creation 7

Text A. Illustration of the basic processes for qualitative spectral comparison

Process for reference library creation and spectral comparison analysis are illustrated in the figure below (**Fig A in S2 Text A**). From top to bottom: (i) spectra are collected from different batches of the same medicine and compiled into a mean spectrum representative of that medicine (with specificities depending on the device, **see S2 Text B-F**). (ii) This mean spectrum is used to build a "library" or database that serves as the comparator against which test samples are compared. (iii) Test samples are scanned and then (iv) the test sample spectra are overlaid with the reference spectrum for visual or computational comparison to determine a pass or fail.

Fig A. Illustration of the process for reference library creation and spectral comparison analysis

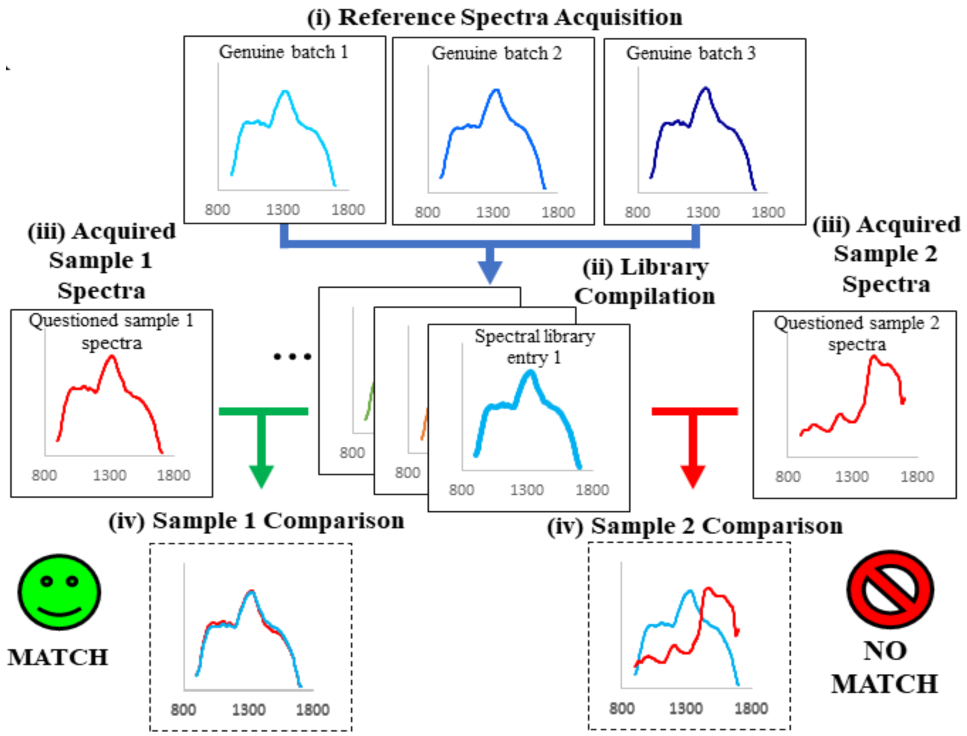

Text B. The MicroPHAZIR RX spectrometer

- For library reference creation, how many scans were taken per sample (on average, specify exceptions)?
  - 5 scans.
- How many spectra per library entry?.
  - 5 spectra.
- Were separate libraries created for samples both in and out of packaging?
  - Yes.
- How was the tablet positioned? (e.g. held by hand; tablet holder *etc.*)
  - The tablet rested on top of the sampling window and was not held by anything or anyone. The sampling window was parallel to the tablet the device was resting on. Blistered tablets were held with the clear side flush against the sampling window.
- Was each scan of a different tablet, or the same tablet in different orientations, or another way?
  - The protocol for tablet sampling was the following:
    - For tablets:
      - Spectra 1 = Tablet #1 Side #1
      - Spectra 2 = Tablet #1 Side #2
      - Spectra 3 = Tablet #2 Side #1
      - Spectra 4 = Tablet #2 Side #2
      - Spectra 5 = Tablet #3 Side #1
    - For tablets still in the blister packaging:
      - Spectra 1 = Tablet #1
      - Spectra 2 = Tablet #2
      - Spectra 3 = Tablet #3
      - Spectra 4 = Tablet #4
      - Spectra 5 = Tablet #5
  - Note: this protocol was used for any sample that had enough tablets. For cases with fewer tablets than the specified protocol, tablets were repeated, but either spun around if the sample was too small, or a different side of the tablet was tested if large enough. If available, different tablets were taken from different batches of the same brand.
- What was the reason behind that decision?
  - To ensure sample placement on the sampling window did not affect the library.
- What dictated how the reference library was created?
  - Experimental sampling strategy/decision, as described above, was designed in consultation with the manufacturer representative. In terms of device configuration for reference library creation, the MicroPHAZIR RX's user manual was used.
- Any potential problems encountered that would cause a bad spectrum (physical/experimental)?
  - Two major problems were encountered:
    - The sampling window on the MicroPHAZIR RX was very large, so for any tablets that were smaller than the sampling window, a cover was used to block ambient light from entering the device. This was not applicable to blistered samples.
    - Round/curved tablets could easily shift position during analysis because there was no tablet holder. Movement of the sample would result in bad spectra being collected. Due to this, the MicroPHAZIR RX was always placed on a table so that the sampling window was parallel to the top of the table and did not move.
- Any potential problems with specific samples encountered?
  - None to report, besides problems mentioned in the previous question.

Text C. The 4500a FTIR spectrometer

- For library reference creation, how many scans were taken per sample (on average, specify exceptions)?
    - 1 scan.
- How many spectra per library entry?
    - 1 spectrum.
- Were separate libraries created for samples both in and out of packaging?
    - No, the instrument cannot scan through packaging.
- How was the tablet positioned? (e.g. held by hand; tablet holder used etc.).
    - Tablets were crushed into a homogenized powder, no positioning. The crushed powder was then placed on the sampling window of the 4500a and pressure was applied to the powder with the device sample press on the window.
- Was each scan of a different tablet, or the same tablet in different orientations, or another way?
    - The library software only allows one spectrum per library entry. For this reason, the tablet was crushed into a homogenized powder, loaded on the sampling window with the sample press, and then scanned.
- What was the reason behind that decision?
    - Following the instruments user manual.
- Any potential problems encountered that would cause bad spectra (physical/experimental)?
    - Major problems (and potential ones):
        - When not enough pressure was applied with the sample press, little to no signal would be obtained.
        - The instrument and or software would occasionally freeze, requiring a reset of the systems.
        - If the tablet was not crushed enough to ensure a homogenous mixture, there is a potential for inconsistencies between spectra of the same medicine.
        - If the sample window and press was not cleaned properly after every sample with isopropanol and a delicate task wipe, there is potential for cross contamination.
- Any potential problems with specific samples encountered?
    - DHAP and ACA medicines have thick coatings, and thus required additional effort in crushing to ensure a proper homogenous mixture.

Text D. The Progeny spectrometer

- For library reference creation, how many scans were taken per sample (on average, specify exceptions)?
  - 3 scans for tablets. Two scans for measurements through the blister, one for each side of a tablet (each side a separate tablet in the same blister pack to preserve the tablet in the blister).
- How many spectra per library entry?
  - All reference spectra were placed in the same master library. Therefore, there were three spectra for tablet samples and two spectra for packaged samples.
- Were separate libraries created for samples both in and out of packaging?
  - All reference spectra were compiled into the same master library, tablets and blistered samples had their own reference spectra.
- How was the tablet positioned? (e.g. held by hand; tablet holder used etc.)
  - The tablets were held by hand in front of, and flush with the sampling cone of the device. This procedure was chosen because the simulated medicines that we tested tended to break with the holder.
- Was each scan of a different tablet, or the same tablet in different orientations, or another way?
  - The protocol for tablet sampling was the following:
    - For tablets:
      - Spectra 1 = Tablet #1 Side #1.
      - Spectra 2 = Tablet #1 Side #2.
      - Spectra 3 = Tablet #2 Side #1.
    - For tablets still in the blister packaging:
      - Spectra 1 = Tablet #1.
      - Spectra 2 = Tablet #2.
  - Note: this was the protocol chosen for any sample that had enough tablets. For samples with fewer tablets than the specified protocol, measurements were repeated on a given tablet. If available, different tablets were taken from different batches of the same brand.
- What was the reason behind that decision?
  - To ensure adequate sampling, to utilize the full capabilities of the master library function, and to keep testing consistent between the Progeny and Truscan RM devices.
- What dictated how the reference library was created?
  - After referencing the user manual and exploring the different functions of the Rigaku spectrometer, we deemed the master library function as the simplest and fastest way of performing experiments.
- Any potential problems encountered that would cause a bad spectrum (physical/experimental)?
  - Round/curved tablets could easily move during analysis because the tablet holder was not utilized. Due to this, the Progeny spectrometer was always placed on a table so that the sampling window was parallel to the top of the table and did not move. The instrument could also be used with one hand and the tablet secured with the other, avoiding direct exposure to the laser (Careful: do not point the laser beam towards the user face or anybody).
  - If one tablet is positioned incorrectly, the analysis could last long. The Progeny averages a series of spectra to obtain the final signal so if the position is not adequate, the instrument could spend 10 min or more averaging spectra until it reached the desired signal-to-noise.
- Any potential problems with specific samples encountered?
  - We were not able to obtain quality spectra for artesunate samples (performing measurements through the vial). So, in order to obtain spectra, we placed the samples in a bag and collected spectra through these containers.

Text E. The Truscan RM spectrometer

- For library reference creation, how many scans were taken per sample (on average, specify exceptions)?
    - 3 scans for tablets. Two scans for measurements through the blister, one for each side of the tablet (each side of a separate tablet in the same blister pack to preserve the tablet in the blister)
- How many spectra per library entry?
    - Only one spectrum was selected per library entry.
- Were separate libraries created for samples both in and out of packaging?
    - Separate library entries were created for samples in and out of packaging.
- How was the tablet positioned? (e.g. held by hand; tablet holder used etc.)
    - We used the tablet holder since the holder did not break the samples, although the device allows the user to hold the tablet by hand.
- Was each scan of a different tablet, or the same tablet in different orientations, or another way?
    - The protocol for tablet sampling was the following:
        - For tablets:
            - Spectra 1 = Tablet #1 Side #1.
            - Spectra 2 = Tablet #1 Side #2.
            - Spectra 3 = Tablet #2 Side #1.
        - For tablets still in the blister packaging:
            - Spectra 1 = Tablet #1.
            - Spectra 2 = Tablet #2.
    - Note: this was for any sample that had enough tablets. For samples with fewer tablets than the specified protocol, tablets were repeated. If available, different tablets were taken from different batches of the same brand.
- What was the reason behind that decision?
    - To ensure adequate sampling, to utilize the full capabilities of the master library function, and to keep testing consistent between the Progeny and Truscan RM.
- What dictated how the reference library was created for each device?
    - Referencing the user manual and a discussion with the manufacturer’s representative.
- Any potential problems encountered that would cause a bad spectrum (physical/experimental)?
    - Round/curved tablets could easily be positioned wrong inside the sample holder. We always double checked that the tablet was centered in the holder.
- Any potential problems with specific samples encountered?
    - We were not able to obtain quality spectra for artesunate samples (through vial measurements). Therefore, we placed the samples in plastic bags and collect the spectra through these containers.

Text F. Difficulties encountered during reference library entries creation

Ideally, five different batches or lots are required for a library based on the MicroPHAZIR RX instruction book. How this differs between medicines and devices, and how the number of batches would affect the results of the performances of the devices is unknown. There are also differences in device specific library creation methods when attempting to introduce variability with batch to batch variation. For the NIR-S-G1 and MicroPHAZIR RX some variability was introduced into a single library entry. For the Progeny and Truscan RM, variability was introduced by creating different library entries for different samples.

Reference libraries for the devices were made by recording the spectra of medicine samples which were assumed to be genuine medicines (obtained from large wholesalers or directly from manufacturers). All samples were sent for UPLC analysis, but results were not received until after completion of much of the testing. Some of the samples whose spectra were recorded as reference library entries were found to be poor quality. As a result, we did not have access to good reference library comparators for the affected brands, and it was decided to discard results from testing of all seven affected brands. Reference library creation differed between all instruments due to the wide variety of data capture and software capabilities for each device. There was very limited medicine batch to batch variation in generation of reference library spectra.
