# Supplementary material for: A comparative field evaluation of six medicine quality screening devices in Laos
Source: PLoS Negl Trop Dis. 2021 Sep 30;15(9):e0009674. doi: 10.1371/journal.pntd.0009674 (PMC8483322; doi:10.1371/journal.pntd.0009674)
Supplement: S3 Text — (PDF) [file pntd.0009674.s003.pdf]

S3 Text. Instructing the trainers and inspectors training in the use of the devices

Instructing the trainers

Nine days of training on device use were first given to three Lao postgraduate research pharmacists by the lead chemist overseeing the laboratory evaluation phase. The training consisted of an overview of the chemistry underlying each device, practice in basic operation, common potential errors, avoidance of errors, instruction in retrieving stored data and protocols to make new entries in the reference library, where applicable. Two devices were assigned to each research assistant who wrote or adapted existing standard operating procedures, training materials, and quick-start user guides. The documents were produced in English, reviewed by the lead chemist, and then translated into Lao for training the medicine inspectors and as a reference during inspections.

Inspectors' training

Intensive training was delivered not less than 3 days prior to the inspection visit.

This training consisted of:

1. Presentation/overview of the device and underlying technology.
2. Written SOP instructions.
3. Opportunity to test the device on a 'training set' of medicines, consisting of two to seven different APIs, depending on the device used (different from the APIs of interest), under the supervision and instruction from the trainers, with the SOP available for reference.

During this training session, the Lao pharmacist observers from the LOMWRU Medicine Quality Team noted common problems that the inspectors experienced with the devices in order to refine the time and motion recording form for the inspection phase.

The inspectors who received the intensive training also received the rudimentary training prior to the inspection visit.

Rudimentary training was given separately for each device immediately prior to the inspection visit. On arrival for the inspection visit, all inspectors (including those who had received intensive training) received verbal instructions on how to use the device, and had 15 minutes to practice using the device on a single blister of genuine medicine. During this 15-minute period, the trainer was available to answer questions.

All the inspectors were provided with a Quick guide in Lao language, irrespectively of the type of training.
