# Supplementary material for: A comparative field evaluation of six medicine quality screening devices in Laos
Source: PLoS Negl Trop Dis. 2021 Sep 30;15(9):e0009674. doi: 10.1371/journal.pntd.0009674 (PMC8483322; doi:10.1371/journal.pntd.0009674)
Supplement: S5 Text — Text A. 4500a FTIR Single Reflection. Table A. Results from evaluation pharmacy inspections with the 4500a FTIR by four inspectors. Table B. Results from sample set testing for the 4500a FTIR: AL and OFLO sample set tested twice by a total of 4 inspectors. Text B. MicroPHAZIR RX. Table A. Main errors made by the three inspectors during the evaluation pharmacy inspections with the MicroPHAZIR RX. Table B. Performance of the MicroPHAZIR RX during evaluation pharmacy inspections by three inspectors. Table C. Results from sample set testing with the MicroPHAZIR RX (SMTM, OFLO, and AL sample sets, each tested once by one inspector). Text C. Minilab. Table A. Results from Minilab testing of sample sets conducted by 3 FDQCC Lao technicians. Text D. NIR-S-G1 (Beta Version). Table A. Main errors made by four inspectors during the evaluation pharmacy inspections with the NIR-S-G1. Table B. Performance of the NIR-S-G1 during evaluation pharmacy inspections by four inspectors. Table C. Results from sample set testing with NIR-S-G1. Text E. Paper Analytical Devices (PAD). Table A. Performance of the PAD during evaluation pharmacy inspections by four inspectors. Figure A. Inspector record sheet (left) for an AZITH sample (in blue pen). Lane interpretation instructions for AZITH are given (right). Table B. Main errors made by four inspectors during the evaluation pharmacy inspections with the PAD. Table C. Results from sample set testing–Paper analytical devices. Text F. Progeny. Table A. Number of samples tested and scans performed using analyse or application functions during four inspections of the evaluation pharmacy with the Progeny. Table B. Performance of the Progeny during evaluation pharmacy inspections by four inspectors. Table C. Results from four sample sets tests (SMTM by two inspectors, OFLO and AL by one inspector each) with the Progeny. Text G. Truscan RM. Table A. Results from evaluation pharmacy inspections with Truscan RM by four inspectors. Table B. Performance [file pntd.0009674.s005.pdf]

S5 Text. Results of the evaluation by device

Text A. 4500a FTIR Single Reflection 1
Text B. MicroPHAZIR RX 7
Text C. Minilab 12
Text D. NIR-S-G1 (Beta Version) 14
Text E. Paper Analytical Devices (PAD) 19
Text F. Progeny 26
Text G. Truscan RM 34

Text A. 4500a FTIR Single Reflection

The 4500a FTIR was set up to return the six highest matches of the sample spectrum to the reference library entry, provided that the 'hit quality' was greater than 0.80. After discussion with the expert chemist, the following procedure was agreed for interpreting the results: if the tested medicine appeared in the six highest matches with a 'hit quality' > 0.9, the sample would be classed as a 'pass'. If the tested medicine appeared in the six highest matches with a 'hit quality' of <0.9, it should be flagged as suspicious and the test repeated as per other spectrometers protocol[1]. Prior to inspecting the pharmacy, however, the instructions given to inspectors were incorrect, due to a misunderstanding by the trainer. The inspectors were told during training that if the specific brand/API name **was not returned as the top match**, they should treat the sample as suspicious, and repeat the test two times. The final result would come from the most commonly occurring in the three tests.

The 'scan results' below (**Table A in S5 Text A**) are the results returned by the device, as recorded in the device memory, applying the rule that a 'hit quality' of > 0.9 to the correct brand and API can be taken as a 'pass', whereas any 'hit quality' < 0.9 to the correct brand and API should be regarded as a fail. 'User decision' refers to the final decision recorded by the inspector on their record

[1] If the first scan resulted in a 'pass', then the result was recorded as a 'pass'. If the first scan resulted in a 'fail', then two more scans were performed. The interpretation of the three scan results was done as follow: if the two subsequent scans are 'fail' then the sample is considered as 'fail'; if the two subsequent scans are 'pass' then the sample is considered as 'pass'; if one subsequent scan is 'pass' and one is 'fail' then the sample is considered as a 'fail'.

sheet during the inspection and is influenced by the training delivered. The ‘user decision’ results thus should be interpreted with caution as the performance of the 4500a FTIR in the field evaluation may have been underestimated (similarly, regarding only the scan result may lead to an overestimate of the device performance on the field, as it does not take into account potential user interpretation error).

Table A. Results from evaluation pharmacy inspections with the 4500a FTIR by four inspectors. Numbers in parentheses are the numbers including all brands of medicines tested, including samples from brands subsequently found to have reference library spectra obtained from poor quality reference samples (as per UPLC analyses). Numbers in red are highlighted to indicate a 'wrong' classification by device and/or user.

API	Number of samples	Number of scans	Scan result[a]				User decision (sample)[b]				Extra scans due to instructions error[c]	Extra scans due to device error[d]
			TN	FN	FP	TP	TN	FN	FP	TP		
**ACA**	4 (4)	6 (6)	5 (5)	0 (0)	1 (1)	0 (0)	3 (3)	0 (0)	1 (1)	0 (0)	2 (2)	1 (1)
**ART**	4 (4)	5 (5)	5 (5)	0 (0)	0 (0)	0 (0)	3 (3)	0 (0)	1 (1)	0 (0)	1 (1)	0 (0)
**DHAP**	0 (4)	0 (6)	0 (6)	0 (0)	0 (0)	0 (0)	0 (3)	0 (0)	0 (0)	0 (1)	0 (2)	0 (0)
**AL**	5 (5)	9 (9)	3 (3)	0 (0)	0 (0)	6 (6)	1 (1)	0 (0)	1 (1)	3 (3)	2 (2)	0 (0)
**OFLO**	9 (9)	9 (9)	9 (9)	0 (0)	0 (0)	0 (0)	9 (9)	0 (0)	0 (0)	0 (0)	0 (0)	0 (0)
**SMTM**	3 (6)	3 (8)	3 (8)	0 (0)	0 (0)	0 (0)	3 (6)	0 (0)	0 (0)	0 (0)	2 (2)	0 (0)
**AZITH**	6 (6)	8 (8)	7 (7)	0 (0)	1 (1)	0 (0)	6 (6)	0 (0)	0 (0)	0 (0)	0 (0)	1 (1)
**Total**	**31 (38)**	**40 (51)**	**32 (43)**	**0 (0)**	**2 (2)**	**6 (6)**	**25 (31)**	**0 (0)**	**3 (3)**	**3 (4)**	**7 (9)**	**2 (2)**

TN: true negative; TP: true positive; FN: false negative; FP: false positive
[a] *Indicates the result given by the device, as recorded by the inspector, and checked by the study team in the device memory post-inspection. Where 'match quality' is > 0.9, the device result is deemed 'genuine' with no further testing required. Match quality < 0.9 means the test result has deemed the sample 'suspicious'.*
[b] *As recorded by the inspector on the record sheet, vs UPLC results*
[c] *Interpretation error was caused by the inspectors being given the wrong instructions as to how interpreting the matches given by the device*
[d] *According to device memory*

Overall EP inspections, 51 scans of a total of 38 samples[2] were performed with the device during four inspections of the pharmacy by four medicine inspectors, including samples/scans from brands subsequently found to have reference library spectra obtained from poor quality reference samples (**Table A in S5 Text A**).

User interpretation errors resulted in a total of seven unnecessary additional scans in the EP inspections (nine if we include all brands tested). As mentioned previously, this was due to an error in the training delivered and is therefore not a fair reflection of the accuracy of the device.

During evaluation pharmacy inspections, three samples out of 31 (9.7%) tested against genuine reference library samples that were wrongly classified as failing because the inspectors were incorrectly taught how interpret the device's results. These errors were made by three inspectors, two with rudimentary training, and one with intensive training. All interpretation errors were due to the

[2] A 'sample' here is defined as a single dosage unit from a unique blister stocked in the evaluation pharmacy. A 'scan' refers to a single result returned by the device on one sample.

medicine not being returned as the top match in the table of results, and therefore being wrongly identified as ‘suspicious’ by the inspector.

User interpretation errors resulted in a total two unnecessary additional scans inspections in SSM (nine if we include all brands tested). As mentioned previously, this was due to an error in the training delivered and is therefore not a fair reflection of the accuracy of the device (**Table 2**).

Table B. Results from sample set testing for the 4500a FTIR: AL and OFLO sample set tested twice by a total of 4 inspectors. Numbers in parentheses are the numbers including all brands of medicines tested, including samples from brands subsequently found to have reference library spectra obtained from poor quality reference samples (as per UPLC analyses). Numbers in red are highlighted to indicate a ‘wrong’ classification by device and/or user.

API	Device results (scans)[a]				Inspector classification of samples (sample)[b]				Device error (scans)[a]	Device error (samples)[a]
	TN	FN	FP	TP	TN	FN	FP	TP		
**AL**	2 (12)	0(0)	0(0)	8(12)	2(4)	0(0)	0(2)	4(6)	0(0)	0(0)
**OFLO**	10(10)	1(1)	0(0)	6(6)	8(8)	1(1)	0(0)	3(3)	1(1)	1(1)
**Total**	**12 (22)**	**1(1)**	**0(0)**	**14(18)**	**10(12)**	**1(1)**	**0(2)**	**7(9)**	**1(1)**	**1(1)**

TN: true negative; TP: true positive; FN: false negative; FP: false positive
[a] *Indicates the result given by the device, as checked by the study team in the device memory post-inspection. Where ‘hit quality’ is > 0.9, the device result is deemed ‘genuine’ with no further testing required. Hit quality < 0.9 means the test result has deemed the sample ‘suspicious’.*
[b] *As recorded by the inspector on the record sheet, vs UPLC results*

Across the four EP inspections and SSM, a total of three device errors were noted: two in evaluation pharmacy testing and one in sample set testing. In one error in the EP, the device failed to return a result for genuine Augmentin (‘no match’ found). In the second error, the device failed to match the targeted brand of AZITH (of note, none of the six ‘matched’ brands displayed by the device were brands containing AZITH). In both cases, additional scans were rerun and the overall classification of the sample was correct. In the third error in the SSM, a substandard brand of OFLO was identified as genuine (with a matching value of 0.925). In all cases, there was no identifiable user error.

Another observed error (apart from interpretation of the result, which can be attributed to incorrect training, as mentioned above) was forgetting to rename the sample spectrum after acquisition, leading to the result being stored with a default file name in the device memory. This had

no direct consequences during the EP inspections where results were also noted on paper, but would affect the traceability of sample results in practice.

Even with these instructions errors, the median number of samples (IQR) wrongly categorized during evaluation pharmacy inspections was 1.0 (0.3-1.0). The number of samples wrongly categorized was significantly lower than for initial visual inspections ($p < 0.05$, Wilcoxon rank sum). Comparing between devices, the 4500a was also significantly lower than the number wrongly categorized in inspections with the PAD ($p < 0.05$) but the percentage of samples wrongly categorized over all inspections was not significantly different to that of any of the other spectrometers.

In sample set testing, one out of 18 (5.6%) samples was wrongly categorized (false negative) because of a device error (see above). Overall, this proportion of wrongly categorized samples across the four inspections was not significantly different from any other devices tested ($p > 0.05$).

Median (IQR) total time taken per sample during sample set testing was 5 min 16 sec, significantly longer than the other spectrometers, ($p < 0.01$, mixed effects generalized linear regression), but significantly less time than with the PAD and Minilab (both of which took significantly longer per sample, $p < 0.001$, mixed effects linear regression). Broken down by phase, the majority of the extra time taken was concentrated in sampling [median sampling time = 242 sec (179-320) sec compared to 50 (32-67) sec for the NIR-S-G1 (fastest device), $p<0.001$], which is consistent with the need to crush the sample prior to testing and for cleaning the device in between samples. The 4500a FTIR is significantly faster ($p < 0.001$, mixed effects linear regression) than all other devices except the MicroPHAZIR RX for the device testing phase.

USER OPINION – 4500A FTIR SINGLE REFLECTION

Immediately after inspection with the 4500a FTIR, all inspectors reported that the device was easy-to-use, and produced trustworthy results. They liked the table of matches, with its list of API and percentage match, as this helped with identifying the contents (medicines of unknown identity) and quality of samples. However, all inspectors also commented that testing involved a large number of steps and crushing and calibration were time-consuming. They felt this would limit its use in pharmacy inspections, especially if many samples were to be tested.

In the focus group discussions, one inspector liked that the device is different than other devices in that there is no need to select any information to run a sample. However, its heavy weight, that makes it hard to carry in the field, was a recurring issue during the focus group discussions. As in the post-inspection survey, it was often raised that the sampling procedure is too complicated: crushing samples and cleaning the sampling window. ***"It's quite wasting time having to crush samples and cleaning carefully between each test even when we test the same sample."*** Two inspectors claimed the results given by the device are reliable, especially when they saw, while testing twice the same sample, that the matching values in the two tests were very similar. One of the inspectors who also used the Progeny spectrometer expressed his preference for the Progeny because there is no need to set-up, clean the device, crush the sample, takes less space, and it is handheld.

In both group discussions the issue of the lack of space for the 4500a FTIR and the computer in the pharmacy outlets was raised.***"[…] in most of the big pharmacies in our country there's no place to test, people queue for hours to get their medicines; there's no way to place the heavy device like this and computer and if we want to test it's just rarely possible.***" However, they all agreed it should be suitable in manufacturers and distributors where there is a laboratory.

All four inspectors suggested that the device should be lighter. One inspector recommended that the device lid cover could integrate a computer screen to avoid the need for a separate computer. One inspector would like to have an accessory to collect the left-over powder after testing the samples.

Text B. MicroPHAZIR RX

Although the MicroPhazir RX has a built-in barcode scanner that can be used by the operator to correctly select the appropriate reference library, it was not utilized. None of the primary packaging of the samples tested in our study had barcodes to present.

Overall, 65 scans of a total of 44 samples[3] were performed with the device during four inspections of the pharmacy by three medicine inspectors. These included samples/scans from brands subsequently found to have reference library spectra obtained from poor quality reference samples (**Table A in S5 Text B).**

Table A. Main errors made by the three inspectors during the evaluation pharmacy inspections with the MicroPHAZIR RX. Numbers in parentheses are the numbers including all brands of medicines tested, including samples from brands subsequently found to have reference library spectra obtained from poor quality reference samples (as per UPLC analyses). Results from one inspection (10 samples, 10 scans) are removed because of concerns over the reference library uploaded to the device. Numbers in red are highlighted to indicate a 'wrong' classification by device and/or user.

API	Total of samples tested	Total scans performed	Samples tested using wrong method	Scans performed using wrong method[a]
**ACA**	5(5)	9(9)	1(1)	2(2)
**ART**	5(5)	7(7)	0(0)	0(0)
**DHAP**	0(5)	0(7)	0(0)	0(0)
**AL**	6(6)	11(11)	1(1)	1(1)
**OFLO**	8(8)	11(11)	1(1)	1(1)
**SMTM**	2(7)	3(8)	0(0)	0(0)
**AZITH**	8(8)	12(12)	1(1)	2(2)
**Total**	**34(44)**	**51(65)**	**4(4)**	**5(5)**

[a] *'wrong method' refers to the inspector either failing to put the sample in the device before testing, or selecting the wrong reference library (see text below)*

[3] A 'sample' here is defined as a single dosage unit from a unique blister stocked in the evaluation pharmacy. A 'scan' refers to a single result returned by the device on one sample.

Table B. Performance of the MicroPHAZIR RX during evaluation pharmacy inspections by three inspectors. Results for samples from brands subsequently found to have reference library spectra obtained from poor quality reference samples (as per UPLC analyses) are not presented and results from one inspection (10 samples, 10 scans) are removed because of concerns over the reference library uploaded to the device. Numbers in red are highlighted to indicate a 'wrong' classification by device and/or user.

Scans performed against correct reference library					Inspector classification of samples [a]				Samples wrongly categorised[b]-user interpretation error
**API**	**TN**	**FN**	**FP**	**TP**	**TN**	**FN**	**FP**	**TP**	
**ACA**	6 (6)	0 (0)	0 (0)	0 (0)	5 (5)	0 (0)	0 (0)	0 (0)	0 (0)
**ART**	7 (7)	0 (0)	0 (0)	0 (0)	5 (5)	0 (0)	0 (0)	0 (0)	0 (0)
**DHAP**	0 (7)	0 (0)	0 (0)	0 (0)	0 (5)	0 (0)	0 (0)	0 (0)	0 (0)
**AL**	3 (3)	0 (0)	0 (0)	6 (6)	3 (3)	0 (0)	0 (0)	3 (3)	0 (0)
**OFLO**	8 (8)	0 (0)	0 (0)	0 (0)	8 (8)	0 (0)	0 (0)	0 (0)	0 (0)
**SMTM**	3 (8)	0 (0)	0 (0)	0 (0)	2 (7)	0 (0)	0 (0)	0 (0)	0 (0)
**AZITH**	9 (9)	0 (0)	0 (0)	0 (0)	8 (8)	0 (0)	0 (0)	0 (0)	0 (0)
**Total**	**36 (48)**	**0 (0)**	**0 (0)**	**6 (6)**	**31 (41)**	**0 (0)**	**0 (0)**	**3 (3)**	**0 (0)**

TN: true negative; TP: true positive; FN: false negative; FP: false positive
[a]*Sample classification as recorded by the inspector on the record sheet, regardless of reference library used*
[b]*Total number of samples wrongly classified (=FP + FN) over all four inspections*

Five of 51 (9.8%) scans performed by the three inspectors using a genuine reference library were performed with a user error (see **Table A in S5 Text B**). All of these mistakes were made by the same inspector, who had received rudimentary training. Of these five, two were performed against the wrong reference library entry where the correct brand and API were selected, but the inspector selected the reference library spectrum for 'through packaging' testing rather than 'not through packaging'. The inspector recognized the mistake and did not record these scan results. The remaining three scans made in error were from failing to insert the sample under the sample cover before running the test. No samples was misclassified as a result of these errors (**Table B in S5 Text B Table**).

Apart from these, no other user mistakes were noted by observers during inspections with the device. The ability of the user with rudimentary training to recognize a wrong result due to operating error and self-correct to improve accuracy suggests that the device is relatively easy-to-use, even with a minimal level of training, hence improving its reliability in a field-setting.

Results from testing of three SSM inspections (SMTM, OFLO, and AL) are reported in **Table C in S5 Text B** [4]. The MicroPHAZIR RX correctly categorized the 50% API samples of both OFLO and SMTM in all four tests as failures. There were 2 device errors (2 FP on one sample of OFLO) over a total of 33 scans (6.1%), leading to 1 genuine sample being wrongly selected as suspicious by one inspector out of 13 samples tested (7.7%).

Table C. Results from sample set testing with the MicroPHAZIR RX (SMTM, OFLO, and AL sample sets, each tested once by one inspector)[a]. Numbers in parentheses are the numbers including all brands of medicines tested, including samples from brands subsequently found to have reference library spectra obtained from poor quality reference samples (as per UPLC analyses). Numbers in red are highlighted to indicate a 'wrong' classification by device and/or user.

API	Device results (scans) - for scans performed against correct reference library				Inspector classification of samples (sample) [b]				Device errors (scans)	Device errors (samples)	N° Scans with wrong library selected
	TN	FN	FP	TP	TN	FN	FP	TP			
**AL**	2(6)	0(0)	0(0)	7(9)	1(2)	0(0)	0(0)	2(3)	0(0)	0(0)	0(0)
**OFLO**	3(3)	0(0)	2(2)	4(4)	3(3)	0(0)	1(1)	2(2)	2(2)	1(1)	0(0)
**SMTM[a]**	1(3)	0(0)	0(0)	6(6)	1(3)	0(0)	0(0)	3(3)	0(0)	0(0)	0(0)
Total	6(12)	0(0)	2(2)	17(19)	5(8)	0(0)	1(1)	7(8)	2(2)	1(1)	0(0)

a We believe that an error occurred during the editing of the reference library of the device by the investigators (in order to update the sample names in the library), leading to increased number of errors from the device during one of four inspections. Consequently, we excluded this sample set testing results from the present table
b sample classification as recorded by the inspector on the record sheet, regardless of reference library used

Pairwise-comparison over all devices using mixed effects model (with device as the main factor, adjusting on training, sample, and clustering by inspector) suggest this was not significantly better than any other device.

Training (in both rudimentary and intensive sessions) was given for the use of the sample cover, a calibration holder modified by laboratory team for small tablets to prevent from ambient light interferences. However, most of the inspectors chose not to use the sample cover during the evaluation pharmacy inspections or the sample set testing, possibly contributing to some of the observed device errors[5] particularly with the simulated medicines in the sample sets (the simulated medicines tablets are small, and ambient light can easily interfere with the collected spectra). Artesunate was tested

[4] One further test of the SMTM sample set is not reported due to the poor quality reference library used in this inspection.
[5] Refers to an inherent error from the device (i.e. with no noticeable user error)

inside the glass vial by all inspectors, with the MicroPHAZIR RX returning the correct result in all seven tests.

Median time per sample in SSM was 2 minutes 14 sec, making the MicroPHAZIR RX significantly faster than all the other devices ($p < 0.001$) except for being slightly but significantly slower than the NIR-S-G1 ($p<0.001$). Median total time spent in the evaluation pharmacy with the MicroPHAZIR RX was 50 min 6 sec, significantly longer when compared to the initial visual inspection without device ($p=0.007$).

Due to the relatively fast speed of analysis, one inspector felt able to perform multiple tests on the same sample. Even when a 'pass' result was obtained in the first test, more tests were performed giving greater him/her confidence in the device results.

USER OPINION – MICROPHAZIR RX

Immediately after inspection with the MicroPHAZIR RX, all of the inspectors felt that the MicroPHAZIR RX was a reliable, precise device, returning comprehensive results which gave confidence in the quality of a sample and provided very useful information on the potential identity of suspicious samples. There were minor comments on its usability: two inspectors commented on the long calibration time, which they felt might hinder its use in routine inspections; another commented that the buttons were quite hard to press, making it harder to use than some of the other devices. The sample window indicator, which shows the inspector whether the sample window is sufficiently covered by the medicine to produce a reliable result when sampling, was cited as a helpful additional feature, giving the inspector additional confidence in their sampling technique. The ability of the device to test through packaging was also felt by the inspectors to increase its usefulness in the field.

During the focus group discussions, although the device was often described as easy to use, comfortable and fast enough to scan samples, some drawbacks were mentioned. One inspector mentioned the long time to perform the set-up and calibration as a barrier for routine inspection in

the pharmacy. When asking about what they did not like, three inspectors agreed that the device is heavy and hard to carry. One inspector mentioned that the device froze during the drug inspection and all the records made in the pharmacy were lost, which made her think that the device would waste her time. Because the difficulty to navigate with the current buttons was mentioned, suggestions for improvement of the device focused on the device design. Improvement of the navigating system (e.g. a touch screen system) and of the portability were mentioned. ***"It takes times to type each letter and when we used the device for long time."*** One inspector stated to dislike the handle of the device and would rather have a stationary device. ***"I would change the design, it would be likely no handle part but just use the device stationary (lay device on a surface), reduce the weight of device and smaller size, typing button should be easier to type."***

Text C. Minilab

Of the evaluation pharmacy samples tested (40 genuine and three falsified medicines), all were correctly categorised by the Minilab (TLC and disintegration).

Overall, median (IQR) total time per sample processing in sample set testing was 34 minutes 23 seconds (25 min 40 sec – 90 min 8 sec), significantly higher than any of the other devices tested ($p < 0.001$). All the phases (sampling, analysing, interpreting/recording) took significantly longer compared to other devices. It should be noted that the technicians ran several samples concurrently on the same TLC plate, so the total time to complete the different samples tests allowed us to only calculate an estimate of the total time per sample. However, there is significant sample preparation required for each sample, including preparation of two reference sample solutions, as well as time for the development of the TLC, inevitably contributing to the much longer total time per sample.

It should be noted that there was significant variation in the time taken to test samples for the Minilab among the three FDQCC technicians, which was consistent with user experience and familiarity with the device. Though all FDQCC technicians have received official training and are actively involved in delivering training in use of the Minilab to provincial staff, the observers noticed significant differences in self-confidence between users. For example, one technician conversed with observers during testing (despite instructions not to) and appeared to lack confidence in testing technique, repeatedly re-checking written protocols and also with colleagues in the laboratory at various stages of the testing process.

In sample set testing, all genuine medicines and 0% API/wrong API samples (n=3) were correctly identified (**Table A in S5 Text C**). All 50% API (n=2) samples tested were incorrectly identified as genuine (false negative), consistent with other studies which show reduced sensitivity of the Minilab for testing non-extreme deviations from the stated content.

Table A. Results from Minilab testing of sample sets conducted by 3 FDQCC Lao technicians. Numbers in red are highlighted to indicate a 'wrong' classification by device and/or user.

API	Results (Tests)[a]				Results (Samples)[b]			
	TN	FN	FP	TP	TN	FN	FP	TP
**AL**	5	0	0	6	2	0	0	3
**SMTM**	6	2	0	4	3	1	0	2
**OFLO**	8	2	0	2	4	1	0	1
**Total**	**19**	**4**	**0**	**12**	**9**	**2**	**0**	**6**

[a]*Results of all separate TLC tests run (i.e.. equivalent to one lane on a TLC plate)*
[b]*Overall sample classification by Lao technician*
FDQCC, Food and Drug Quality Control Center

Text D. NIR-S-G1 (Beta Version)

Results from the evaluation pharmacy inspections by four independent inspectors are given below (**Table A and B in S5 Text D**). Over four inspections, 81 tests were performed, and 53 samples tested[6], including samples from brands subsequently found to have reference library spectra obtained from poor quality reference samples.

Table A. Main errors made by four inspectors during the evaluation pharmacy inspections with the NIR-S-G1. Numbers in parentheses are the numbers including all brands of medicines tested, including samples from brands subsequently found to have reference library spectra obtained from poor quality reference samples (as per UPLC analyses).

API	Total samples tested	Samples tested against wrong reference library[a]	Total scans performed	Scans against wrong reference library[b]
**ACA**	7 (7)	4 (4)	9 (9)	4 (4)
**ART**	6 (6)	3 (3)	13 (13)	9 (9)
**DHAP**	0 (6)	0 (4)	0 (8)	0 (6)
**AL**	6 (6)	1 (1)	11 (11)	1 (1)
**OFLO**	8 (8)	0 (0)	11 (11)	0 (0)
**SMTM**	4 (10)	1 (3)	8 (18)	1 (7)
**AZITH**	8 (8)	2 (2)	11 (11)	2 (2)
**Total**	**39 (53)**	**11 (17)**	**63 (81)**	**17 (29)**

[a] *When the sample was tested against the wrong reference library entry*
[b] *according to device memory*

[6] A ‘sample’ here is defined as a single dosage unit from a unique blister stocked in the evaluation pharmacy. A ‘test’ refers to a single result returned by the device on one sample.

Table B. Performance of the NIR-S-G1 during evaluation pharmacy inspections by four inspectors. Numbers in parentheses are the numbers including all brands of medicines tested, including samples from brands subsequently found to have reference library spectra obtained from poor quality reference samples (as per UPLC analyses). Numbers in red are highlighted to indicate a ‘wrong’ classification by device and/or user.

API	Device error[a] (No. of scans)	Scans performed by user against the right reference library[b]				Inspector classification of sample[c]				Samples wrongly categorised[d]
		TN	FN	FP	TP	TN	FN	FP	TP	
ACA	0 (0)	5 (5)	0 (0)	0 (0)	0 (0)	7 (7)	0 (0)	0 (0)	0 (0)	0 (0)
ART	1 (1)	3 (3)	0 (0)	1 (1)	0 (0)	4 (4)	0 (0)	2 (2)	0 (0)	2 (2)
DHAP	0 (0)	0 (2)	0 (0)	0 (0)	0 (0)	0 (4)	0 (0)	0 (1)	0 (0)	0 (1)
AL	0 (0)	5 (5)	0 (0)	1 (1)	4 (4)	4 (4)	0 (0)	0 (0)	2 (2)	0 (0)
OFLO	0 (0)	11 (11)	0 (0)	0 (0)	0 (0)	8 (8)	0 (0)	0 (0)	0 (0)	0 (0)
SMTM	3 (3)	4 (8)	0 (0)	3 (3)	0 (0)	3 (7)	0 (0)	1 (3)	0 (0)	1 (3)
AZITH	0 (0)	9 (9)	0 (0)	0 (0)	0 (0)	8 (8)	0 (0)	0 (0)	0 (0)	0 (0)
**Total**	**4 (4)**	**37 (43)**	0 (0)	**5 (5)**	**4 (4)**	**34 (42)**	0 (0)	**3 (6)**	**2 (2)**	**3 (6)**
		**46 (52)**				**39 (50)**				

TN: true negative; TP: true positive; FN: false negative; FP: false positive
[a]*with no observable user error*
[b]*including only scans performed against right reference spectra*
[c]*sample classification as recorded by the inspector on the record sheet, regardless of reference library used*
[d]*total number of samples wrongly classified (=FP + FN) over all four inspections*

The most common user mistake identified in the evaluation pharmacy was the selection of the wrong reference library to compare the sample scanned. Of the 81 scans performed, 29 (35.8%) affecting 17 samples were made with the user selecting the wrong reference library for comparison. All of the wrongly-selected reference libraries were of the correct API but of the incorrect brand, highlighting the importance of acquiring and appropriately using formulation-specific reference libraries for every medicine to be tested with the device. Nineteen (65.5%) of these mistakes were made by one inspector, who received the ‘rudimentary’ training. For six (20.7%) of the 29 scans performed using the wrong reference library (affecting six samples), the user recognized the mistake, repeated the test against the correct reference library, and did not include the initial incorrect result when deciding on the final classification. As a result, this did not lead to final sample misclassification. Of the 17 samples where the user selected the wrong reference library for comparison, eleven were from brands with genuine reference spectra. Of these, two (18.2%) were misclassified as suspicious as a result of using the wrong reference library (i.e. false positive: the

inspector did not realise their mistake, the device returned a 'fail' result, and the sample was subsequently wrongly classified as suspicious).

Considering only samples for which genuine reference spectra were present, 39 samples were scanned over the four inspections, of which five (12.8%) samples failed the test. Three of these five (60%) were false positives (two resulting from user error, and one from device error[7]), and two (40%) were true positives. The median (IQR) number of samples wrongly categorised per inspection was 1 (0-2) out of a median (IQR) of 10 (7-12) samples tested. Overall, the proportion of wrongly categorised samples across the four inspections was 7.7% (1.6-20.9%), which was not significantly different from any other devices tested ($p > 0.05$), except the PAD that resulted in a higher proportion of wrongly categorised samples ($p = 0.005$).

Five of 63 (7.9%) scans (affecting 3/39 samples) performed without observed user error gave false results (5 false positive). The most commonly affected medicine was SMTM, for which 3 of 8 scans (on 2 samples of the same brand, Strimside®) during the same inspection gave a false positive result with no observed user error. Hence, these two genuine samples were incorrectly classified using the NIR-S-G1 as poor quality.

During sample set testing, 18 samples with good quality reference library spectra were tested (**Table C in S5 Text D**). The inspector with rudimentary training and who made nineteen mistakes in the evaluation pharmacy consistently selected the wrong reference library entry over all six samples in the set, leading to two samples being wrongly categorized as fail (false positives).

[7] Refers to an inherent error from the device (i.e. with no observable user error in device use)

Table C. Results from sample set testing with NIR-S-G1. Numbers in parentheses are the numbers including all brands of medicines tested, including samples from brands subsequently found to have reference library spectra obtained from poor quality reference samples (as per UPLC analyses). Numbers in red are highlighted to indicate a 'wrong' classification by device and/or user.

API	Device results (scans) - for scans performed against correct reference library				Inspector classification of samples (sample)[a]				Device errors (scans)	Device errors (samples)	N° Scans with wrong library selected
	TN	FN	FP	TP	TN	FN	FP	TP			
**OFLO**	4(4)	0(0)	0(0)	4(4)	6(6)	0(0)	2(2)	4(4)	0(0)	0(0)	10(10)
**AL**	2(5)	0(0)	0(2)	8(11)	2(4)	0(0)	0(1)	4(6)	0(2)	0(1)	0(0)
**Total**	**8(11)**	**0(0)**	**4(6)**	**16(19)**	**8(10)**	**0(0)**	**2(3)**	**8(10)**	**0(0)**	0(1)	10(10)

[a]*Sample classification as recorded by the inspector on the record sheet, regardless of reference library used*

Median total time spent in the evaluation pharmacy by the inspectors with the NIR-S-G1 was the shortest of all tested devices (32 min 33 sec), and was not significantly different to the time taken to perform the initial visual inspection without a device (25 min 16 sec, $p = 0.443$). This is consistent with sample set testing by the inspectors, in which the NIR-S-G1 had the fastest median (IQR) time per sample [(1 min 34 sec (61 sec - 1 min 52 sec)] and was significantly faster than for all other devices tested ($p < 0.001$).

USER OPINION – NIR-S-G1

From immediate post-inspection feedback, the medicine inspectors who used this device noted the advantages as they saw them as:

- Size: small enough to be easily portable (3 out of 4 inspectors)
- Fast analysis time
- Easy-to-use compared to other devices they tested

All medicine inspectors felt the NIR-S-G1 would be useful to them in their routine pharmacy inspections, but all stated that the lack of capability to update the reference library locally was a key limitation to its use.

During the focus group discussions, four inspectors agreed that it is the most portable device and the easier and faster operated device by running an application on the phone. ***“It is the easiest to operate, portable and good scanning device.”*** Two out of four inspectors however, underlined the limited reference library entries and acknowledged that is would gain usability if users could create their own reference libraries. In addition, they all agreed that a great improvement would be the ability to test other formulations such as liquids. When asking their level of trust on the NIR-S-G1 results, all four inspectors fairly trusted the device: ***“We give more than 70% of reliability.”*** Four inspectors believed that the device would be suitable to test in many different sites of the pharmaceutical supply chain: pharmacies, manufacturer’s sites, distributor’s sites and border check points.

Text E. Paper Analytical Devices (PAD)

Over four inspections by four inspectors with the PAD in the evaluation pharmacy, 29 samples were tested (one test per sample), and 22 errors were counted, leading to a total of 11 samples (37.9%) being wrongly identified as suspicious[8] (**Table A in S5 Text E**).

Table A. Performance of the PAD during evaluation pharmacy inspections by four inspectors. In the field evaluation, only genuine medicines were available for the API that the PAD can test. Thus, the only possible results here are True Negative or False Positive.

**API**	**Inspector classification of sample[a,b]**		**Number of samples wrongly identified as suspicious**
	**TN**	**FP**	
**Amoxicillin**	4	0	0
**ART[c]**	N/A	N/A	N/A
**Piperaquine**	2	2	2
**AL[c]**	N/A	N/A	N/A
**OFLO**	2	6	6
**Sulfamethoxazole**	7	0	0
**AZITH**	3	3	3
**Total**	**18**	**11**	**11**

TN: true negative; FP: false positive
[a]All inspectors performed only one test per API and per sample. Consequently, the number of samples tested equals the number of tests performed
[b]This is the classification of the sample, as given on the inspector record sheet
[c]The PAD used in this study do not have the capability to test artesunate or artemether-lumefantrine.

In the written protocol, and also in both rudimentary training, and intensive training, the inspectors were instructed to photograph the PAD result three minutes after removal from the solvent (they were provided with a smartphone), prior to reading and interpreting the result. In practice, this was done inconsistently[9], and only 14 of the 29 PAD results in the evaluation pharmacy were photographed.

Different types of errors were observed:

[8]The PAD were only used to test genuine medicines in the evaluation pharmacy because the only poor quality medicines stocked in the pharmacy were falsified AL. The PAD currently cannot test AL

[9] The first inspector to test the PAD did not take any photos during evaluation pharmacy inspection (8 samples) and sample set testing (6 samples). In the subsequent inspections, inspectors were prompted to photograph the PAD.

1. Wrong lane read by the user: the user recorded results in the wrong lane columns on the inspector record sheet (**Fig A in S5 Text E**), suggesting that the incorrect lanes were being interpreted by the inspector

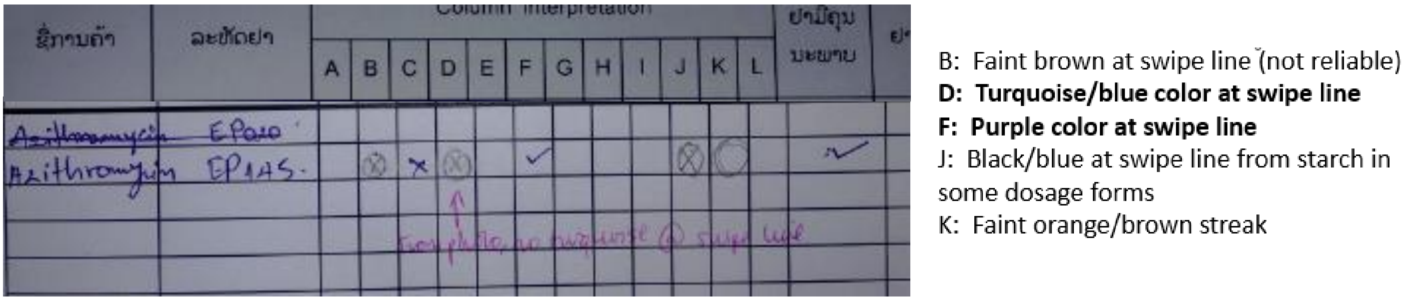

Figure A. Inspector record sheet (left) for an AZITH sample (in blue pen). Lane interpretation instructions for AZITH are given (right). The inspector has read colours in lane B and F rather than D and F (wrong lane read); has not realised the mistake and classified the sample as genuine (correct categorisation, wrong reasons).

2. Wrong colour: one or more of the PAD lanes did not show the expected colour according to the supplied reference photographs for the medicine tested. This was confirmed by comparison with the photograph when possible[10] – i.e. the colour pattern displayed on the PAD was not consistent with a genuine sample[11]
3. User interpretation error: the user correctly read and recorded the colour pattern, but came to the wrong conclusion about the quality of the sample based on the pattern seen. For example for ACA, the inspector would correctly note that the PAD revealed 'green' in lane C, 'dark green' in lane F, and no 'cherry red' in lane K. Then, the inspector would deem the sample 'genuine' despite lane K requiring the 'cherry red' colour to qualify the sample as a genuine ACA medicine.

[10] Where a photograph existed, a 'wrong colour' result recorded on the inspector record sheet was verified by review of the photograph. Where no photograph existed, the inspector record is the only evidence. If the photograph was not taken at the advised time point (3 minutes after removal from the water), it is possible that the colours observed in the photographs are inaccurate, which should be considered a 'user' rather than 'device' error. The time elapsed between removal of the PAD from the water and taking the photograph was not recorded, hence we cannot further categorise error in these results.

[11] All evaluation pharmacy samples tested with the PAD were good quality (confirmed by UPLC); the PAD used here cannot evaluate artemisinin-based medicines, and the only poor quality medicine stocked in the pharmacy was artemether-lumefantrine.

Table B. Main errors made by four inspectors during the evaluation pharmacy inspections with the PAD

API	Tests/Samples[a]	Type of error			
		Wrong lane read by the user	Wrong colour in PAD lane[b]		Wrong user interpretation of lane results
			Inspector record	Photo confirmation[c]	
**Amoxicillin**	4	1	1	0	0
**ART[d]**	N/A	N/A	N/A	N/A	N/A
**Piperaquine**	4	2	2	1	2
**AL[d]**	N/A	N/A	N/A	N/A	N/A
**OFLO**	8	1	5	2	0
**Sulfamethoxazole**	7	0	1	0	1
**AZITH**	6	1	5	1	1
**Total**	**29**	**3**	**14**	**4**	**4**

[a]All inspectors performed only one test per API and per sample. Consequently the number of samples tested equals the number of tests performed
[b]As recorded on the inspector record sheet. This was confirmed by review of a photograph, where the photograph existed (see 'Photo confirmation').
cThe photo was examined by one investigator of the study after the end of the study, which is subject to error of interpretation
d The PAD used in this study do not have the capability to test artesunate or artemether-lumefantrine.

The most common error that occurred was a PAD lane displaying the wrong or no colour and hence leading to the wrong result (14 errors, 4 confirmed by review of photographed PAD results) (**Table B in S5 Text E**). This occurred most commonly for samples of AZITH (lane F showing no colour – an error which is known to developers) and OFLO (lane D not showing a blue colour – it was noted by the developers that this colour is quick to fade). It is notable that this mistake did not occur in sample set testing where eight OFLO samples were tested (**Table C in S5 Text E**).

An inspector with rudimentary training continued to use the same visibly contaminated water as the solvent for multiple PAD during testing in the evaluation pharmacy, despite being told to use fresh water after every test. The contamination is likely from the medicine applied to the PAD falling into the water or from chemicals from the PAD that may have contaminated the water. In addition in the evaluation pharmacy, none of the inspectors tested any sample more than once, although during the training they were all notified to perform a re-run test in case of failure of a test as specified by the developer of the PAD.

Reading the wrong lanes or the inspector coming to the wrong conclusion about the interpretation of the displayed colour bar code was another ‘user interpretation’ errors made both in the evaluation pharmacy and in sample set testing. This wrong conclusion occurred despite each lane being independently ‘read’ correctly. Interestingly, for four of 29 samples in the evaluation pharmacy, although a number of mistakes were made during PAD use (user reading the wrong lanes, or the device displaying the wrong colour), overall the sample was correctly categorized as genuine.

These results support the impression that the training given may have been insufficient for all inspectors and more practice with result interpretation should be given prior to use in the field.

Table C. Results from sample set testing – Paper analytical devices Numbers in red are highlighted to indicate a ‘wrong’ classification by device and/or user.

Device results[a]					Inspector classification of sample[b]				Wrong lane read by user	Wrong colour (confirmed by photo)	User interpretation of lane results
**API**	**TN**	**FN**	**FP**	**TP**	**TN**	**FN**	**FP**	**TP**			
**OFLO**	3	0	0	1	8	1	0	3	0	0	0
**SMTM**	6	1	0	5	4	2	2	4	2	1	3
**Total**	**9**	**1**	**0**	**6**	**12**	**3**	**2**	**7**	**2**	**1**	**3**

[a]*This refers to the actual device result, as determined by review of the photograph by the investigator of the study (available for only 16 of 24 samples tested. Note that this is subject to error of interpretation.*
[b]*Inspector classification of the sample, as recorded on inspector record sheet.*

The median (IQR) number of samples wrongly categorised per evaluation pharmacy inspection was 2 (1-6), which was not significantly different to initial visual inspection ($p = 0.6311$, Wilcoxon rank sum). The median (IQR) number of samples tested per inspection was 7.5 (5 – 9), which was not significantly lower than for other devices ($p > 0.05$, Dunn test). However, significantly longer time was spent in the pharmacy [median (IQR) 93 min 20 sec (48 min 48 sec - 133 min 36 sec)] compared to any of the other devices tested ($p < 0.05$ for all paired comparisons). Overall, the proportion (95% CI) of wrongly categorised samples across the four inspections was 37.9% (20.7-57.7%), which was significantly different from all other devices tested ($p < 0.05$).

The median (IQR) time to test one sample in sample set testing [10 min 20 sec (9 min 22 sec – 11 min 56 sec)] was significantly longer than for any of the other devices tested, apart from the Minilab (34 min 23 sec, $p < 0.0001$). The extent of the time spent on the PAD was most pronounced

in the device testing phase[12], during which the time taken for the device to produce a result was significantly longer than for any other devices ($p < 0.001$) except the Minilab (median device testing time = 18 min 54 sec ($p < 0.001$). Sampling time was comparable to the 4500a FTIR (which also requires sample destruction) and interpreting and recording time was not significantly different to the 4500a FTIR or Progeny ($p > 0.05$). A large proportion of the time taken in the device testing phase was the time taken for water to be drawn up the card (set to 3 minutes by the developer) which cannot be reduced.

Of the three false negative results obtained in sample set testing, one was a substandard OFLO sample. The other two were falsified SMTM samples which correctly gave a 'falsified' colour barcode as observed by the investigator on the picture of the PAD, but were interpreted incorrectly by the user. Both false positive results were a consequence of user interpretation error as well.

Although the PAD make no claim to be able to detect medicines with reduced API, three of the four 50%API samples tested in sample set testing were correctly identified as suspicious.

USER OPINION – PAPER ANALYTICAL DEVICES

Immediately after inspection, the inspectors liked the simplicity of the PAD, with their lack of reliance on electricity or other instrumentation. However, all commented that the 'wet chemistry' element would limit its usefulness in a routine pharmacy inspection setting because of the need to prepare the sample, have working space to carry out the analysis, and the relatively long analysis time. Two out of four inspectors felt the analysis and interpretation of the final result was difficult, and one inspector who had received rudimentary training specifically commented that they did not have much confidence in the results. However, another inspector who also received rudimentary training stated that he enjoyed doing the visual comparison with the reference and found the PAD

[12] For the PAD, the analysis phase began when the PAD is placed into the water , included time for the water to reach the end of PAD; removal of the PAD from water; and waiting for 3 minutes for colours to develop (mayat same time possibly preparing next sample). Ended at the end of the 3 minutes or the soonest time after the 3 minutes for development that the inspector picked up the PAD or picked up their pen to record the result.

easy-to-use; his only suggested improvement was an increase of the number of API that the PAD are able to test.

During the focus group discussions, the low-cost of the PAD, their practicability and the need for only few accessories and no other chemicals were again claimed as of great interest, underlying the benefits for use in low and middle-income countries. However, the inspectors did not like the difficulty to prepare the sample by crushing. The inspectors were also worried that the volume of water used for running the test may not be sufficient, or too much, without knowing the impact of inadequate amount of water on the results. One inspector stated that the sampling process was complicated and not standardized enough:

"We need to crush the sample which we do not know if it was fine enough then press it on paper and we cannot tell if it was well spread. For water that we used as a solvent, we didn't know how much we need to pour in the tank."

When asking about the trust on the device results, most inspectors were quite concerned that the interpretation of the colour code was too much user-dependent.

"Even if we can see the color and can compare with the reference, we can make mistake on interpretation." "For example, in the protocol it's said it's pink and in reality it's a faded pink so it depends on the user's eyesight and his/her decision. So, it's difficult to tell the actual color."

All four inspectors felt that it may not be appropriate to test medicine quality in pharmacies or at the distributors sites because of the time taken to run the test, and also because of the destructive feature of the PAD. They mentioned the lack of budget to buy the medicines as a barrier for destructive technique use in the field. However, two inspectors acknowledged that it would be useful to test raw materials in manufacturers sites.

Most of the comments about the features to improve the PAD usability in the routine practice were on the interpretation of the colour code:

"The shown color should be a clear straight color for example pink is pink, not pinkish-purple."

It was also suggested to integrate a 'ditch' into the PAD so that the sample is placed in a more standardized way.

"It should have a little ditch for sample placing for example we have to fulfil the ditch then dip into water."

Text F. Progeny

Although the Progeny has a built-in barcode scanner that can be used by the operator to correctly select the appropriate reference library, it was not utilized. None of the primary packaging of the samples tested in our study had barcodes to present.

The Progeny has two scanning modes, 'analyse' and 'application'. For the field evaluation, inspectors were instructed to use the 'analyse' function to inspect all medicines initially because it compares the sample's spectra to the entire reference library of spectra. If the 'top match' result using the 'analyse' function did not match the brand and API tested, the test was repeated using the 'application' function where the user selects the reference spectrum for the brand of interest. A 'no match' result was obtained during an 'analyse' test if the sample spectrum failed to achieve a greater than 80% match with any stored spectra in the reference library.

A summary of results from testing in the evaluation pharmacy is given in **Table A in S5 Text F.**

Table A. Number of samples tested and scans performed using analyse or application functions during four inspections of the evaluation pharmacy with the Progeny. Numbers in parentheses are the numbers including all brands of medicines tested, including samples from brands subsequently found to have reference library spectra obtained from poor quality reference samples (as per UPLC analyses)

API	Number of samples	Total scans
**ACA**	4 (4)	9 (9)
**ART[a]**	6 (6)	16 (16)
**DHAP**	0 (4)	0 (4)
**AL**	4 (4)	9 (9)
**OFLO**	7 (7)	12 (12)
**SMTM**	3 (7)	6 (14)
**AZITH**	6 (6)	7 (7)
**Total**	**30 (38)**	**53 (71)**

[a]Samples were scanned through the glass vials by the inspectors, although reference library was created by scanning through a replacement packaging (see text below)

Table B. Performance of the Progeny during evaluation pharmacy inspections by four inspectors. Numbers in parentheses are the numbers including all brands of medicines tested, including samples from brands subsequently found to have reference library spectra obtained from poor quality reference samples (as per UPLC analyses). Numbers in red are highlighted to indicate a 'wrong' classification by device and/or user.

API	Analyse function (Scans)				Application function (Scans)			Application function (Scans)[a]				Inspector classification of sample[b]			
	Match: brand and API	Match: API, not brand	Match: wrong API	No match	Number of samples tested	Number of scans	Wrong library chosen	TN	FN	FP	TP	TN	FN	FP	TP
**ACA**	3	1	0	0	3	5	1	4	0	0	0	4	0	0	0
**ART[d]**	0	0	0	6	6	10	1	4	0	5	0	3	0	3	0
**DHAP**	0 (4)	0	0	0	0	0	0	0	0	0	0	0 (4)	0	0	0
**AL**	0	2	0	2	3	5	0	3	0	0	2	3	0	0	1
**OFLO**	3	3	1	0	3	5	2	3	0	0	0	5	0	2	0
**SMTM**	1 (2)	3[c] (5)	0	0	1 (3)	2 (6)	0 (2)	2 (4)	0	0	0	3 (7)	0	0	0
**AZITH**	6	0	0	0	0	0	0	1	0	0	0	4	0	0	0
**Total**	**13 (18)**	**9[c] (11)**	**1**	**8**	**16 (18)**	**27 (31)**	**4 (6)**	**17 (19)**	**0**	**5**	**2**	**22 (31)**	**0**	**5**	**1**

[a] *Scans performed against correct reference library*
[b] *Sample classification as recorded by the inspector on the record sheet, regardless of reference library used*
[c] *One user performed 'analyse' test twice in the same sample*
[d] *Samples were scanned through the glass vials by the inspectors, although reference library was created by scanning through a replacement packaging (see text below)*

Table C. Results from four sample sets tests (SMTM by two inspectors, OFLO and AL by one inspector each) with the Progeny. Numbers in parentheses are the numbers including all brands of medicines tested, including samples from brands subsequently found to have reference library spectra obtained from poor quality reference samples (as per UPLC analyses). Numbers in red are highlighted to indicate a 'wrong' classification by device and/or user.

API	Total		Analyse function [a]						Application function (scans)			Application function result (scans)				Inspector classification of sample[c]			
	Number of samples	Total scans Analyse function + application function	For genuine samples			For falsified samples		For 50%/80 % samples	Number of samples tested	Number of scans	Wrong method chosen	TN	FN	FP	TP	TN	FN	FP	TP
			Match= Brand and API	Match= API but not brand	Match= Incorrect API	Match= correct main excipient	No match	Match[b]= API not Brand											
**SMTM**	8 (12)	19 (26)	0(0)	2(6)	0(0)	2(2)	2(2)	2(2)	7 (12)	10 (14)	0	2 (6)	3 (3)	0 (0)	5 (5)	2(6)	2(2)	0(0)	4(4)
**OFLO**	6	11	2(2)	2(2)	0(0)	1(1)	0(0)	1(1)	3 (3)	5 (5)	0	1(1)	0(0)	2(2)	2(2)	3(3)	1(1)	1(1)	1(1)
**AL**	3 (6)	12 (18)	0(0)	1(3)	0(0)	2(2)	0(0)	N/A[d]	3 (6)	6 (12)	0	2(6)	0(0)	0(0)	4(6)	1(3)	0(0)	0(0)	2(3)
**Total**	**17 (18)**	**41 (44)**	2(2)	**5(11)**	0(0)	**5(5)**	**2(2)**	**3(3)**	**14 (21)**	**21 (31)**	**0**	**5(13)**	**3(3)**	**2(2)**	**11(13)**	**6(12)**	**3(3)**	**1(1)**	**7(8)**

[a]*Analyse function was performed only once per sample so the number of scans is equal to the number of samples*
[b]*Only False negative results were obtained for substandard samples using the analyse function*
[c]*Sample classification as recorded by the inspector on the record sheet, regardless of reference library used*
[d]*There was no50%/80% API samples included in the AL sample set*

A total of 38 samples were tested in the evaluation pharmacy inspections, and 71 scans completed, including samples/scans from brands subsequently found to have reference library spectra obtained from poor quality reference samples (**Table A in S5 Text F**). Of those tested against a genuine reference library spectrum, 13 samples correctly matched the brand on the first 'analyse' scan. Nine samples matched the API but not the brand, and eight samples[13] (7 genuine, 1 non-genuine) showed no match or matched the wrong API and brand (**Table B in S5 Text F**).

In the evaluation pharmacy, of the eight samples (nine tests) where the 'analyse' function matched the API but not the brand, no samples were wrongly classified overall. These matched API were seen most often for SMTM and OFLO, for which the largest number of brands were included in the pharmacy. For eight of these nine tests (seven of eight samples), the inspector followed up the 'analyse' result with the appropriate method using the 'application' function, which matched the correct brand and API. The remaining one sample was declared 'genuine' by an inspector, who had received intensive training, without running a further 'application' function test (deviation from the suggested protocol).

One issue with using the 'application' function is the opportunity for the user to select the wrong reference library spectrum for comparison. Of 30 scans made using the 'application' function in the evaluation pharmacy, the wrong library was chosen in six scans (five samples involved). From these 6 scans, two scans (two samples) by an inspector with intensive training who may have realised the mistake and repeated the test using the correct reference library. The remaining four scans (three samples) were by an inspector with rudimentary training who may have realized the mistake and repeated the test using the correct reference library for two out of four scans (two samples). Errors in selecting appropriate reference libraries could have been

[13] Nine tests with 'analyse' function as one inspector performed the 'analyse' test twice on the same sample (deviation from protocol).

avoided if the built-in barcode had been used. However, none of the samples tested in our study had barcodes to present.

Over the four EP inspections, two samples of OFLO (different brands) were wrongly classified as fail (both by one inspector with rudimentary training). In both cases, the ‘Analyse’ scan did not match to API and brand, and the ‘Application’ scans were either incorrectly performed (wrong reference library chosen) or incorrectly interpreted by the user.

Artesunate, sampled inside the glass vial, presented a particular problem for the ‘analyse’ mode: no match was obtained for any of the six samples tested. All six samples were then tested with the ‘application’ function. Of the ten scans performed correctly (using the correct reference library), four returned a ‘pass’ result whereas six returned a ‘fail’. As a result, three samples failed (false positive) overall. However, as for the Truscan RM, the reference library spectrum was taken from powder inside a polythene bag, due to problems with obtaining a strong signal from the sample inside packaging during the laboratory phase. The inspectors were unable to extract the powder during the pharmacy inspections (the glass vial is, appropriately, very difficult to open) and hence sampled inside packaging for all artesunate tests. This problem also occurred during the laboratory phase of the project.

In sample set testing (**Table C in S5 Text F**), the Progeny failed to match the correct brand and API for the two genuine SMTM samples tested, but both were correctly categorised on further testing with the ‘application’ function. All falsified samples were correctly identified as such. Despite matching to the API on ‘application’ function, one genuine OFLO sample was incorrectly identified as suspicious after failing two ‘application’ tests against the correct reference library. The source of this error is unclear: of the 11 genuine OFLO samples tested across both pharmacy and sample set, this error occurred for only one sample.

Overall, the number of user errors made during sample set testing was lower than in the evaluation pharmacy: in 29 scans using the application function, the wrong method was selected only once (one sample of a brand subsequently removed from the analysis due to having a reference library spectrum from a poor quality sample). There was no significant difference in the number of samples wrongly categorised during evaluation pharmacy inspection with the Progeny compared to the initial visual inspection ($p = 0.0792$, Wilcoxon rank sum). Overall, the proportion (95% CI) of wrongly categorised samples across the four inspections was 8.3% (1.0-27.0%), which was not significantly different from any other devices tested ($p > 0.05$), except the PAD that resulted in a higher proportion wrongly categorised ($p = 0.023$).

Median (IQR) total time per sample in sample set testing was 4 min 32 sec, significantly higher than the two NIR devices (NIR-S-G1 and MicroPHAZIR RX, $p < 0.05$). Total test time per sample was not significantly different between the two Raman devices (Progeny and Truscan RM, $p = 0.514$)[14], but device testing and 'recording' times were significantly longer for the Progeny compared to the Truscan RM (median analysing time = 20 sec vs 87 sec; respectively $p = 0.001$).

USER OPINION – PROGENY

Overall, immediately after inspection with the Progeny, the inspectors were impressed by the large number of medicines in the reference library, which they felt would enhance its utility in pharmacy inspection and felt that the result obtained was precise and reliable. Two out of four inspectors cited the ability of the device to return a 'closest' match for suspicious samples as their favorite feature. However, it was also felt to be quite slow to scan, and three inspectors out of four

[14] Note that the validity of the test may be weakened by the very small sample size and wide range of the Truscan results (70.5 – 471 s) compared to the Progeny (98 – 363 s).

commented that the touchscreen was not very responsive, increasing the time taken to record sample details. It was felt to be heavy and therefore less portable compared to the other handheld spectrometers. Three inspectors also commented that the supplied tablet holder was difficult to use with smaller tablets.

In the focus group discussions, inspectors stressed the ability of the device to scan through packaging as a plus. However, three inspectors agreed that the device is heavy.

"It's a heavy device due to the steel body, I was always worried to drop the device every time I used it."

Two of the inspectors mentioned that the typing system is hard even though it's a touchscreen system. The slow set-up and analysis were mentioned by one of them. An inspector claimed that the analysis is fast but that entering the sample details after the analysis takes long because of the difficulty to type with the touch screen. It is important to note that the Progeny does have buttons to select settings, select experiments, and type information; however, in most cases these were not utilized to enter samples details after the scanning. The Progeny used in this study was an ex-demo model. The manufacturer stated that this might be the reason why the touchscreen was slowly responsive. However, with the time constraints for this project, we were not able to return the device to the manufacturer to investigate this issue.

The lack of adaptability of the tablet holder for small size tablets analysis was mentioned twice as a problem.

"Smaller tablets always dropped out, I have to stand vertically then place the sample."

When questioned about how much they trust the device results, two inspectors from different group discussions says they trust it **'50-50'.**

One acknowledged the ability of the device as a screening technique only: ***"[…] the device cannot be sure for 100%, we only know the identification, for example we set the device if it's greater than 0.90 then it passed, if the second time you scan the same sample the match value is reduced by 0.05, it is substandard. I'd say it's just a first/basic scanning before sending to the big laboratory ".***

Interestingly, another inspector claimed to trust the device to around 90-95%. One potential reason for this may be that this inspector liked having matching value displayed by the device (rather than a binary 'pass/fail' result): ***"One good point I like to tell is it has correlation number like how much is the match between the sample and the reference. And it also shows the other matches."***

All inspectors agreed that the device would be useful for drug inspections in different levels of the pharmaceutical supply chains: pharmacies, manufacturers, distributors and border checkpoints. Although one inspector mentioned its heaviness and large size as a potential barrier to inspect pharmacy outlets. Suggestions for development resulted from the previous comments: a tablet holder that can be used for small tablets, the device body should be lighter, and a more responsive touchscreen. One inspector also suggested an in-device calibration process rather than the current calibration that has to be run with the provided standard vial and its specific holder.

Text G. Truscan RM

Although the Truscan RM has a built-in barcode scanner that can be used by the operator to correctly select the appropriate reference library, it was not utilized. None of the primary packaging of the samples tested in our study had barcodes to present.

Overall, 52 scans of a total of 38 samples[15] were performed with the device during four inspections of the pharmacy by four medicine inspectors, including samples/scans from brands subsequently found to have reference library spectra obtained from poor quality reference samples (**Table A in S5 Text G**).

Table A. Results from evaluation pharmacy inspections with Truscan RM by four inspectors. Numbers in parentheses are the numbers including all brands of medicines tested, including samples from brands subsequently found to have reference library spectra obtained from poor quality samples (as per UPLC analyses).

API	Total samples tested	Total scans performed	Samples tested against wrong reference library	Scans against wrong reference library	Samples wrongly classified
**ACA**	4 (4)	4 (4)	0 (0)	0 (0)	0 (0)
**ART[a]**	5 (5)	14 (14)	0 (0)	0 (0)	*0* (0)
**DHAP**	0 (4)	0 (4)	0 (0)	0 (0)	0 (0)
**AL**	4 (4)	7 (7)	2 (2)	4 (4)	0 (0)
**OFLO**	8 (8)	9 (9)	3 (3)	3 (3)	0 (0)
**SMTM**	5 (8)	5 (8)	1 (1)	2 (2)	0 (0)
**AZITH**	5 (5)	6 (6)	0 (0)	0 (0)	0 (0)
**Total**	**31 (38)**	**45 (52)**	**6 (6)**	**9 (9)**	**0 (0)**

[a]Samples were scanned through the glass vials by the inspectors, although reference library was created by scanning through a replacement packaging (see text below)

The most notable finding was the inability of the TruScan RM to correctly screen for parenteral artesunate powder quality through the glass vial. Every test of artesunate through the glass vial performed with the device gave a false positive result (14/14 tests of five samples),

[15] A 'sample' here is defined as a single dosage unit from a unique blister stocked in the evaluation pharmacy. A 'scan' refers to a single result returned by the device on one sample.

leading to all five genuine samples being wrongly identified as suspicious (**Table B in S5 Text G**). This is most likely due to all the inspectors choosing to scan the artesunate through the glass vial packaging, whereas the reference library entry was generated for the artesunate powder re-packaged into a thin polythene bag[16]. In the evaluation pharmacy inspections, all the inspectors chose to sample artesunate through the supplied glass vial, likely because the glass vial is sealed with a metal cap, which is very difficult to remove (impossible to remove without tools such as scissors or a knife).

[16] The reference library used in the inspections was generated by the expert users at Georgia Tech. It was created from artesunate powder sampled through a thin polythene bag. This decision was taken by the laboratory team as they were unable to obtain a good quality spectrum of artesunate while it remained in the supplied glass vial. The field study trainers may have not been clear enough to the medicine inspectors that sampling through packaging would be a problem, and the inspectors were informed that the Truscan RM could sample through all transparent packaging, including the glass vial.

Table B. Performance of the Truscan RM during evaluation pharmacy inspections by four inspectors. Results for samples from brands subsequently found to have reference library spectra obtained from poor quality reference samples (as per UPLC analyses) are not presented. Numbers in red are highlighted to indicate a 'wrong' classification by device and/or user.

API	Device error (No. of scans)	Scans performed against correct reference library[b]				Inspector classification of samples[c]			
		TN	FN	FP	TP	TN	FN	FP	TP
**ACA**	0	4	0	0	0	4	0	0	0
**ART[a]**	*(14)*	0	0	*(14)*	0	0	0	*(5)*	0
**DHAP**	0	0	0	0	0	0	0	0	0
**AL**	0	1	0	0	6	1	0	0	3
**OFLO**	0	5	0	0	0	8	0	0	0
**SMTM**	0	3	0	0	0	5	0	0	0
**AZITH**	0	6	0	0	0	5	0	0	0
**Total**	**14**	**19**	**0**	***(14)***	**6**	**23**	**0**	***(5)***	**3**

TN: true negative; TP: true positive; FN: false negative; FP: false positive
[a]Samples were scanned through the glass vials by the inspectors, although reference library was created by scanning through a replacement packaging. These results might thus be distorted because of the potential effect of different packaging on the performance of the device (see text below).
[b]Including only scans performed against the correct reference library entry (according to device memory) and discounting brands for which the reference spectrum was recorded from a poor quality sample
[c]Sample classification as recorded by the inspector, regardless of reference library used but discounting brands for which the reference spectrum was recorded from a poor quality sample

In addition, the volume of powder in the artesunate vial is very small and needed to be tapped down to the bottom of the vial when sampled in order to maximise the probability of the device returning the correct result. Observers noted that this was not done routinely by the inspectors for the first scan of the sample, although it was done if a second or third scan of the vial was carried out. Due to the small volume of powder, the overall Raman signal is likely to be weak, and further attenuated by the glass packaging. For larger volumes of powder in vials, it is possible that the stronger signal would allow sampling through the glass vial.

In one test, the inspector wrote down on the recording sheet that he thought the sample was genuine despite the 'fail' result given by the device, but acknowledged that he would be obliged to take the sample for further testing anyway. We did not test the ability of devices to check the

authenticity of the accompanying 5% sodium bicarbonate vial required for reconstituting the artesunate for injection.

The most common user error was the selection of the wrong reference library with which to compare the sample scanned. Of the 52 scans of 38 samples performed, five (9.6%) scans affecting five samples (13.1%) were made with the user selecting the wrong reference library for comparison. In each case, the reference library selected was for a medicine containing the same API but of a different brand. The device returned the correct result in each case, and no samples were wrongly categorised as a result.

After removing results from testing of artesunate for the reasons described above, no samples were wrongly categorised by inspectors during evaluation pharmacy testing. As a result of this lack of variation in observations, it was not possible to compare performance against other devices.

In sample set testing, one inspector, who had received 'intensive' training and tested the OFLO sample set failed to select the correct library[17] for 5 out of 6 samples tested (eight scans out of nine), instead selecting a library for the different brand of the same API[18]. This did not lead to any samples being wrongly categorised, supporting the observation in the evaluation pharmacy that this device may be less sensitive to formulation-specific variations than the NIR devices. No other inspectors made method selection errors. The four false negative results recorded by the device were from testing (with no observable user error) three 50%-80% API simulated medicines samples (one OFLO and two SMTM) (**Table C in S5 Text G)**. The Truscan RM gave a 'pass'

---

[17] The Truscan is first run after selecting the formulation-specific library for the tested medicine (called 'method' in the device), giving a 'pass' or 'fail' result. If it fails to match the reference spectrum, the whole library of stored spectra is then automatically searched by the device to find the 'closest match', which is displayed on the screen, together with the 'Fail' result.

[18] The same inspector accounted for 5/9 wrong reference library selections during evaluation pharmacy inspection

result for 4 out of 5 scans, leading to both samples being incorrectly categorised as genuine. The remaining OFLO substandard sample was tested against the wrong reference library entry (wrong brand containing different API selected), though correctly gave a 'false' (true positive) result in all three scans performed. The device correctly identified all 0% API simulated medicines samples and FC/Simulated genuines in sample set testing.

Table C. Results from sample set testing with the Truscan RM. Numbers in parentheses are the numbers including all brands of medicines tested, including samples from brands subsequently found to have reference library spectra obtained from poor quality reference samples (as per UPLC analyses). Numbers in red are highlighted to indicate a 'wrong' classification by device and/or user.

API	Device results - Scans performed against correct reference library[a]				Inspector classification of samples[b]				Number of scans against wrong reference library	Device error (scans)[c]	Device error (samples)[d]
	TN	FN	FP	TP	TN	FN	FP	TP			
**SMTM[c]**	2(6)	2(2)	0(0)	8(8)	2(6)	2(2)	0(0)	4(4)	0(0)	2(2)	2(2)
**OFLO**	5(5)	2(2)	0(0)	4(4)	8(8)	1(1)	0(0)	3(3)	8(8)	2(2)	1(1)
**Total**	**10(14)**	**4(4)**	**0(0)**	**17(17)**	**10(14)**	**3(3)**	**0(0)**	**7(7)**	8(8)	**4(4)**	**3(3)**

TN: true negative; TP: true postive; FN: false negative; FP: false positive
[a]Including only scans performed against the correct reference library entry (according to device memory) and discounting brands for which the reference spectrum was recorded from a poor quality sample
[b]Sample classification as recorded by the inspector, regardless of reference library used but discounting brands for which the reference spectrum was recorded from a poor quality sample
[c]Number of scans for which the device returned an erroneous result with no obvious user error
[d]Number of samples affected by the 'device error (scans)'. NB: this did not necessarily lead to the sample being wrongly categorised as more than one scan per sample was performed.

Median (IQR) total time to test a sample in sample set testing was 2 min 27.5 sec, slower than the NIR-S-G1 (median total time 93.5 s, $p < 0.001$) and the MicroPHAZIR RX ($p = 0.002$), but not significantly different from the Progeny ($p = 0.514$). Device testing and recording times were significantly faster for the Truscan RM compared to the Progeny, the other Raman instrument tested in the present study ($p < 0.001$ and $p = 0.025$, for the device testing and recording times, respectively). This may be due to the device's sampling strategies, signal acquisition, and signal processing. As mentioned above, when the start button is pressed, the Truscan RM first runs a

comparison with the selected formulation-specific library entry for the tested medicine (called ‘method’ in the device). If it fails to match the reference spectrum, then the whole library of stored spectra is automatically searched by the device to find the ‘closest match’, which is displayed on the screen together with the ‘Fail’. The initial scan on the Progeny, on the other hand, was conducted in the present study in the ‘analyse’ mode, which searches through the entire reference library to find the closest match (which is the result displayed on the screen), which might explain the longer device testing time for the Progeny.

There was no significant difference in the number of samples tested or scans performed in evaluation pharmacy testing compared to any of the other devices.

USER OPINION – TRUSCAN RM

Overall, from immediate feedback post-inspection, the inspectors felt that the device could be useful to them in pharmacy inspections, but would be limited by what was perceived to be a slow analysis time relative to the other handheld spectrometric devices and by the relative bulkiness of the carry case. One inspector also had doubts over the reliability of the results, as he correctly identified that the device consistently gave the wrong result for artesunate sampled through the glass vial (see previous section). In addition, the supplied tablet holder was felt by one inspector to be difficult to use with small tablets.

In the focus group discussion, the slow analysis of the Truscan RM was not mentioned during the focus group discussion, but the heaviness was mentioned as compared to the NIR-S-G1. Three out of four inspectors found it is easy and comfortable to use**.** Three inspectors agreed that it is bothersome to change the tablet holder and the cone for scanning through packaging test.

“I think it's annoying to pull out the tablet holder for every new testing.”

All of the inspectors claimed to have at least 70% confidence in the device results. However, they recognized that this was dependent on the users to correctly follow the protocol for making the reference library correctly.

"We trusted the device 70 - 90% because all the settings were already well set. If we performed the procedure correctly on making the reference library, it should work as we have set. Whatever it says Pass or Fail, we'll go with it."

They all felt that the device was suitable to use in different levels of the supply chain: pharmacies, manufacturers' plants, distributors and border check points. In each test performed in the evaluation pharmacy, if the device gave a fail result for the first time they retested again for confirmation. Of these tests, if one failed and one passed, they then tested for the third time:

"We retested a failing sample in the mock pharmacy. If it failed again then we suspect that sample would be spurious."

When asking for desired improvement of the device for their daily work, they mostly focused on the body of the device, which they felt should be lighter. Three inspectors mentioned the usefulness of developing only one 'sample holder' that would enable to scan the tablet both through and out of packaging, instead of the current system in which one cone is used for scanning through the blister and one tablet holder is used for analyzing the tablet out of the packaging. One inspector also suggested to develop ability to test through non-transparent blisters, and to add a search function for the brand names to select the 'method'. The current system only allows to select the brand name in a 'folder of the brands first letter', the desired brand must then be searched for with scroll function through the folder.
