# Supplementary material for: A comparative field evaluation of six medicine quality screening devices in Laos
Source: PLoS Negl Trop Dis. 2021 Sep 30;15(9):e0009674. doi: 10.1371/journal.pntd.0009674 (PMC8483322; doi:10.1371/journal.pntd.0009674)
Supplement: S1 Table — (PDF) [file pntd.0009674.s006.pdf]

S1 Table. Main characteristics and UPLC results of the medicines utilized in the evaluation

Study Code (each blister of the sample)	Study Phase (EP, RL, SSM)	Stated brand name	API name	API Strength (mg)	Expiry Date (mm/ yyyy)	Formulation	Types of packaging	Origin - Quality of Sample	Mass Spectroscopy Result	UPLC Result (%)
G071	RL	Sulfatrim	SMTM	400-80	04/2018	Tablet	O	FC - Genuine	N/A	88¥-89¥
G072	RL	Sulfatrim	SMTM	400-80	10/2017	Tablet	O	FC - Genuine	N/A	93-95
G080	RL	Vactrim	SMTM	400-80	04/2016	Tablet	O	FC - Genuine	N/A	93-98
G137	RL	Ofloxin	OFLO	200	03/2016	Tablet	O	FC - Genuine	N/A	89.9
G259	RL	Ofloxacin	OFLO	200	01/2017	Tablet	O	FC - Genuine	N/A	94.2
SPS20	SSM	Sulfatrim	SMTM	200	03/2019	Tablet	O	FC - Genuine	N/A	90-92
G275	RL	Oflocee	OFLO	200	04/2018	Tablet	O	FC - Genuine	N/A	89.1 ¥ (1st test)<br>96 (2nd test)
G278	RL	Azithromax	AZITH	250	07/2017	Tablet	T	FC - Genuine	N/A	102
G281	RL	Di-flo	OFLO	200	02/2017	Tablet	O	FC - Genuine	N/A	92.4
G311	RL	Strim-Side	SMTM	200	06/2017	Tablet	O	FC - Genuine	N/A	96-93 (1st test)<br>99-99 (2nd test)
G314	RL	Vactrim	SMTM	250	09/2016	Tablet	O	FC - Genuine	N/A	97-98
G317	RL	Oflocee	OFLO	200	12/2020	Tablet	O	FC - Genuine	N/A	89.2 ¥ (1st test)<br>92.3 (2nd test)
G318	RL	Augmentin	ACA	200	03/2018	Tablet	T	FC - Genuine	N/A	92-96
EP063	EP	Ofloxin	OFLO	200	01/2019	Tablet	O	FC - Genuine	N/A	97.4
G324	RL	Azithromax	AZITH	250	08/2018	Tablet	T	FC - Genuine	N/A	95 (1st test)<br>98 (2nd test)
G337	RL	Azithromax	AZITH	250	06/2018	Tablet	T	FC - Genuine	N/A	97
G344	RL	Di-flo	OFLO	200	03/2018	Tablet	O	FC - Genuine	N/A	93.4
G354	RL	OralZicin	AZITH	500	03/2018	Tablet	T	FC - Genuine	N/A	107
G388	RL	Artemether-Lumefantrine	AL	200	09/2016	Tablet	T	FC - Genuine	N/A	115¥-102
G419	RL	Strim-Side	SMTM	200	03/2019	Tablet	O	FC - Genuine	N/A	96-98
G426	RL	Ofloxin	OFLO	200	01/2019	Tablet	O	FC - Genuine	N/A	94.4
G429	RL	Biseptrim	SMTM	60	05/2018	Tablet	O	FC - Genuine	N/A	96-100
G432	RL	Vactrim	SMTM	60	07/2018	Tablet	O	FC - Genuine	N/A	94-135¥ (1st test)<br>95-135¥ (2nd test)<br>94-114¥ (3rd test)<br>93-132¥ (4th test)

Study Code (each blister of the sample)	Study Phase (EP, RL, SSM)	Stated brand name	API name	API Strength (mg)	Expiry Date (mm/ yyyy)	Formulation	Types of packaging	Origin - Quality of Sample	Mass Spectroscopy Result	UPLC Result (%)
G435	RL	Ofloxacin	OFLO	200	08/2018	Tablet	O	FC - Genuine	N/A	93.9 (1st test)<br>93.4 (2nd test)<br>97.4 (3rd test)
G437	RL	Sulfatrim	SMTM	200	09/2020	Tablet	O	FC - Genuine	N/A	80¥-81¥ (1st test)<br>84¥-86¥ (2nd test)<br>87¥-89¥ (3rd test)
G429	RL	D-Artepp	DHAP	250	09/2017	Tablet	O	FC - Genuine	N/A	90.2-99.1
G457	RL	Lumartem	AL	200	06/2016	Tablet	T	FC - Genuine	N/A	No UPLC performed (not enough samples)
EP006/<br>EP007	EP	Augmentin	ACA	200	01/2018	Tablet	T	FC - Genuine	N/A	99-102 (1st test)<br>96-96 (2nd test)
EP114 to EP119/<br>EP120/EP121	EP	Biseptrim	SMTM	250	01/2019	Tablet	O	FC - Genuine	N/A	98-103
G485	RL	Azithromax	AZITH	250	03/2019	Tablet	T	FC - Genuine	N/A	99
EP008	EP	Augmentin	ACA	200	01/2018	Tablet	T	FC - Genuine	N/A	99-103
G526	RL	D-Artepp	DHAP	60	03/2017	Tablet	O	FC - Genuine	N/A	86.9¥-101.3
G529	RL	Cavumox 1G	ACA	250	02/2018	Tablet	O	FC - Genuine	N/A	103-102
G530	RL	Cavumox 1G	ACA	200	09/2017	Tablet	O	FC - Genuine	N/A	100-100
G533	RL	AMK<br>1000 mg	ACA	500	07/2018	Tablet	T	FC - Genuine	N/A	100-77¥ (1st test)<br>97-54¥ (2nd test)
G534	RL	AMK<br>1000 mg	ACA	250	04/2018	Tablet	T	FC - Genuine	N/A	99-78¥ (1st test)<br>99-52¥ (2nd test)
EP112/EP113/G563	EP	Biseptrim	SMTM	200	08/2019	Tablet	O	FC - Genuine	N/A	96-101
EP152/SPS21	EP/SSM	Sulfatrim	SMTM	250	05/2021	Tablet	O	FC - Genuine	N/A	90-92
G542	RL	OralZicin	AZITH	500	09/2018	Tablet	T	FC - Genuine	N/A	108
EP052	EP	Oflocee	OFLO	200	02/2021	Tablet	O	FC - Genuine	N/A	97.2
G547	RL	Artesun	ART	60	06/2019	Vial	T	FC - Genuine	N/A	96.7
G548	RL	Artesun	ART	60	05/2019	Vial	T	FC - Genuine	N/A	97.2
G550	RL	D-Artepp	DHAP	500	02/2018	Tablet	O	FC - Genuine	N/A	91.4-98.4

Study Code (each blister of the sample)	Study Phase (EP, RL, SSM)	Stated brand name	API name	API Strength (mg)	Expiry Date (mm/ yyyy)	Formulation	Types of packaging	Origin - Quality of Sample	Mass Spectroscopy Result	UPLC Result (%)
G551	RL	D-Artepp	DHAP	40-320	12/2017	Tablet	O	FC - Genuine	N/A	87.3¥-103
EP024/G552	EP	D-Artepp	DHAP	40-320	01/2018	Tablet	O	FC - Genuine	N/A	91.9-99.1
EP102 to EP111	EP	Strim-Side	SMTM	400-80	11/2019	Tablet	O	FC - Genuine	N/A	100-97
EP072 to EP076/SPS13	EP/SSM	Di-flo	OFLO	200	08/2018	Tablet	O	FC - Genuine	N/A	95.5 (1st test)<br>91.2 (2nd test)
EP091 to EP100/G556	EP	Vactrim	SMTM	400-80	08/2019	Tablet	O	FC - Genuine	N/A	96-101
EP053/EP054/EP057 to EP059/EP061/EP062/ SPS15	EP/SSM	Ofloxacin	OFLO	200	08/2019	Tablet	O	FC - Genuine	N/A	98.9
SPS16	SSM	Diabeta	Chlorpropamide	250	08/2021	Tablet	T	FC - wrong API	N/A	No SMTM detected
EP009/EP010/G563	EP	Augmentin	ACA	500-125	02/2016	Tablet	T	FC - Genuine	N/A	101-97
EP122 to EP126	EP	Sulfatrim	SMTM	400-80	09/2020	Tablet	O	FC - Genuine	N/A	92-92
EP077 to EP081	EP	Di-flo	OFLO	200	07/2018	Tablet	O	FC - Genuine	N/A	96.9
EP141 to EP143/EP156	EP	Azithromax	AZITH	250	09/2019	Tablet	O	FC - Genuine	N/A	100
EP082 to EP090/EP101	EP	Vactrim	SMTM	400-80	11/2019	Tablet	O	FC - Genuine	N/A	95-101
EP044/SPS14/G569	EP/SSM	Oflocee	OFLO	200	03/2021	Tablet	O	FC - Genuine	N/A	91.2 (1st test)<br>95.5 (2nd test)<br>96.2 (3rd test)
EP055/EP056/EP060/ G570	EP	Ofloxacin	OFLO	200	03/2020	Tablet	O	FC - Genuine	N/A	92.4
EP129 / EP130/G571	EP	Sulfatrim	SMTM	400-80	01/2022	Tablet	O	FC - Genuine	N/A	98-99
EP022 /EP023/ EP025 to EP027	EP	D-Artepp	DHAP	40-320	09/2018	Tablet	O	FC - Genuine	N/A	92.7-99.4
EP012 to EP021/EP154/EP155/ EP159/EP160	EP	Artesun	ART	60	01/2020	Powder for injection	T	FC - Genuine	N/A	99 (1st test)<br>100.4 (2nd test)
EP045 to EP051	EP	Oflocee	OFLO	200	02/2022	Tablet	O	FC - Genuine	N/A	95.8 (1st test)<br>94.9 (2nd test)
EP136 to 140	EP	Azithromax	AZITH	250	03/2020	Tablet	O	FC - Genuine	N/A	102
EP127 to EP128	EP	Sulfatrim	SMTM	400-80	06/2022	Tablet	O	FC - Genuine	N/A	91-92

Study Code (each blister of the sample)	Study Phase (EP, RL, SSM)	Stated brand name	API name	API Strength (mg)	Expiry Date (mm/ yyyy)	Formulation	Types of packaging	Origin - Quality of Sample	Mass Spectroscopy Result	UPLC Result (%)
EP064 to EP071/SPS23	EP/SSM	Ofloxin	OFLO	200	11/2019	Tablet	O	FC - Genuine	N/A	96.1 (1st test) 93.2 (2nd test)
EP001 to EP005/EP157	EP	Augmentin	ACA	500-125	11/2019	Tablet	T	FC - Genuine	N/A	102-99
EP033/EP035 to EP038/SPS22	EP/SSM	Coartem	AL	20-120	06/2015	Tablet	T	FC - Genuine	N/A	88¥-96
EP028 to EP032/ EP039/EP040/SPS09	EP/SSM	Coartem	AL	20-120	08/2017	Tablet	T	FC - Genuine	N/A	91-96
GT-K20-AD-3	RL	Coartem	AL	20-120	05/2017	Tablet	T	FC - Genuine	N/A	103-94
GT-K23-AD-3	RL	Coartem	AL	20-120	06/2017	Tablet	T	FC - Genuine	N/A	106-93
LA16-113	RL	Azithromax	AZITH	250	06/2018	Tablet	O	FC - Genuine	N/A	97
EP144/LA16-150	EP	Azithromax	AZITH	250	02/2018	Tablet	O	FC - Genuine	N/A	102
EP151	EP	Ofloxin	OFLO	200	10/2016	Tablet	O	FC - Genuine	N/A	91.8
LA16-17	RL	Strim-Side	SMTM	400-80	06/2016	Tablet	O	FC - Genuine	N/A	99-98
EP145	EP	Azithromax	AZITH	250	10/2018	Tablet	O	FC - Genuine	N/A	103 (1st test) 104 (2nd test)
LA16-180	RL	Ofloxin	OFLO	200	07/2018	Tablet	O	FC - Genuine	N/A	92.8
EP043	EP	Oflocee	OFLO	200	09/2018	Tablet	O	FC - Genuine	N/A	92.0
LA16-202	RL	Augmentin	ACA	500-125	04/2018	Tablet	T	FC - Genuine	N/A	99-102 (1st test) 99-102 (2nd test)
LA16-38	RL	Strim-Side	SMTM	400-80	05/2018	Tablet	O	FC - Genuine	N/A	100-102
LA16-41	RL	Ofloxin	OFLO	200	10/2017	Tablet	O	FC - Genuine	N/A	92.1
LA16-66	RL	Azithromax	AZITH	250	10/2018	Tablet	O	FC - Genuine	N/A	100
EP011	EP	Augmentin	ACA	500-125	02/2019	Tablet	T	FC - Genuine	N/A	97-98
LA17-03	RL	Augmentin	ACA	500-125	02/2019	Tablet	T	FC - Genuine	N/A	99-103
EP131 to EP135/EP158/LA17-06	EP	OralZicin	AZITH	500	09/2018	Tablet	T	FC - Genuine	N/A	104
*SPS11	SSM	Coartem	AL	20-120	05/2011	Tablet	T	FC – 0%/wrong API	Major Components: Ciprofloxacin Minor Components: Levamisole & Sildenafil	N/A
*SPS10	SSM	Coartem	AL	20-120	05/2011	Tablet	T	FC -0%/ wrong API	Major Components: Chloramphenicol Minor Components: Levamisole & Sildenafil (trace)	N/A
MM16-21	RL	Artemether-Lumefantrine	AL	20-120	07/2017	Tablet	T	FC - Genuine	N/A	101-93
SPS06	SSM	Artemether-Lumefantrine	AL	20-120	07/2017	Tablet	T	FC - Genuine	N/A	104-98

Study Code (each blister of the sample)	Study Phase (EP, RL, SSM)	Stated brand name	API name	API Strength (mg)	Expiry Date (mm/ yyyy)	Formulation	Types of packaging	Origin - Quality of Sample	Mass Spectroscopy Result	UPLC Result (%)
*SPS07	SSM	Artemether-Lumefantrine	AL	20-120	06/2016	Tablet	T	FC - 0% API	Major Components: Sucrose/Lactulose, Glucose/Fructose, Mannitol, and m/z 338 Minor Components: Maltitol	N/A
EP041	EP	Coartem	AL	20-120	06/2016	Tablet	T	FC – 0% API	Major Components: Sucrose/Lactulose, Glucose/Fructose, & Mannitol	N/A
EP042	EP	Coartem	AL	20-120	06/2016	Tablet	T	FC – 0%/wrong API	Major Components: Chloramphenicol & m/z 338 Minor Components: Sildenafil	N/A
EP034	EP	Coartem	AL	20-120	06/2016	Tablet	T	FC – 0%/wrong API	Major Components: Ciprofloxacin & m/z 338 Minor Components: Sildenafil	N/A
SS50-OFLO-CEL-SPS01	SSM	N/A	OFLO	N/A	N/A	Tablet	N/A	SM - 50%	N/A	N/A
EX-CEL-SPS02	SSM	N/A	None	N/A	N/A	Tablet	N/A	SM - 0%	N/A	N/A
SM-SMTM-CEL-SPS03	SSM	N/A	SMTM	N/A	N/A	Tablet	N/A	SM - 100%	N/A	N/A
SS50-SMTM-CEL-SPS04	SSM	N/A	SMTM	N/A	N/A	Tablet	N/A	SM - 50%	N/A	N/A
SM-OFLO-CEL-SPS05	SSM	N/A	OFLO	N/A	N/A	Tablet	N/A	SM - 100%	N/A	N/A

*Sample not tested by UPLC but underwent Mass spectrometry as part of another study - none of the correct API as stated on the packaging were present

ACA: Amoxicillin-clavulanic acid; AL: Artemether-lumefantrine; API: Active Pharmaceutical Ingredient; ART: Artesunate; AZITH: Azithromycin; DHAP: Dihydroartemisinin-piperaquine; EP: Evaluation Pharmacy inspection ; FC: Field-Collected; N/A: Not applicable; OFLO: Ofloxacin; O: Opaque packaging; RL: Reference Library; SSM : Sample Set of Medicines inspection; SM: Simulated medicines SMTM: Sulfamethoxazole-trimethoprim; T: transparent packaging

¥: Out of specification according to the 90-110% range considered in the present study
