# Supplementary material for: A comparative field evaluation of six medicine quality screening devices in Laos
Source: PLoS Negl Trop Dis. 2021 Sep 30;15(9):e0009674. doi: 10.1371/journal.pntd.0009674 (PMC8483322; doi:10.1371/journal.pntd.0009674)
Supplement: S2 Table — (PDF) [file pntd.0009674.s007.pdf]

S2 Table. User satisfaction questionnaire

1. Could you tell us your general feelings/views about the device?
2. Was there anything that you particularly like or dislike about the device?
3. Was there anything that you particularly found difficult when using the device?
4. What was your favourite device feature?
5. Do you think the device could be used, and would be useful for routine outlets inspections in Laos?

    If yes, tell us more about how it could be used for routine outlets inspections in Laos:

    If no, please specify:
