# Supplementary material for: A comparative field evaluation of six medicine quality screening devices in Laos
Source: PLoS Negl Trop Dis. 2021 Sep 30;15(9):e0009674. doi: 10.1371/journal.pntd.0009674 (PMC8483322; doi:10.1371/journal.pntd.0009674)
Supplement: S3 Table — (PDF) [file pntd.0009674.s008.pdf]

S3 Table. Outline of the focus group discussions (5 inspectors per group discussions*)

**1.**	**Set-up**
	The participants were asked to write their name and which devices they tested on a sticker placed in front of them<br><br>The participants were notified that the session was to be recorded to help the investigators with note taking<br><br>- Consent form for recording the discussion were provided<br>- Disclosure that all information will be anonymised, and that they are free to raise any opinion, good or bad
**2.**	**Introduction from the investigators**
	- Acknowledgments and explanation of the purpose of session
**3.**	**Introducing themselves**
	One by one, could you please describe which device(s) you tested?
**4.**	**Devices review:** *On a table, have laid out photos of the devices; each device was shown one by one by the moderator*
	- First, inspectors who used these devices were asked to say:<br>o What they liked?<br>o What they didn't like?<br>o Would they use it in their routine inspection?<br>o Do you have suggestions on how the device could be improved to help your drug inspection further?<br>o Where in the supply chain do they think it would be best used? (*A visual representation of the supply chain was printed and shown by the moderator: manufacturer → border → distributor → outlet)*<br>- Invite comments from inspectors who didn't use them

**5.**	**Sampling strategy-Decision making:** *We are interested in finding out how they decided to test some samples and not others, and to understand on what the decision to select a sample (or not) as suspicious was made*
	- How did it make you feel when the device gave a 'fail' result? What did you do next?<br>- How many times would you test the sample before deciding to treat it as suspicious?
**6.**	**Changing behavior:** *How does introducing the devices change the way of doing inspections?*
	- When you went to the pharmacy without the devices, how did you decide which medicines to inspect?<br>- When you went to the pharmacy with the device, how did you decide which medicines to test with the device?

**One of the 16 inspectors could not join the FGD*
