# Supplementary material for: A comparative field evaluation of six medicine quality screening devices in Laos
Source: PLoS Negl Trop Dis. 2021 Sep 30;15(9):e0009674. doi: 10.1371/journal.pntd.0009674 (PMC8483322; doi:10.1371/journal.pntd.0009674)
Supplement: S4 Table — Extract of the inspection record sheet for (A) Evaluation Pharmacy inspections (B) Sample set of medicines inspections. Note that the record sheets were adapted for the PAD, Progeny, and 4500a FTIR which are interpreted differently than other devices (e.g. inspectors were asked to fill in which column of the PAD they read for interpreting the results). Table A. Evaluation pharmacy inspection. Table B. Sample set medicine inspections. (PDF) [file pntd.0009674.s009.pdf]

S4 Table. Extract of the inspection record sheet for (A) Evaluation Pharmacy inspections (B) Sample set of medicines inspections

Note that the record sheets were adapted for the PAD, Progeny, and 4500a FTIR which are interpreted differently than other devices (e.g. inspectors were asked to fill in which column of the PAD they read for interpreting the results).

Table A. Evaluation pharmacy inspection

Date of Inspection:	Device name:	Inspector Name:

Active Pharmaceutical Ingredient	Brand name	Study sample code	Test through packaging?	Trial 1		Test through packaging?	Trial 2		Test through packaging?	Trial 3		Overall classification		Comment
				PASS	FAIL		PASS	FAIL		PASS	FAIL	Pass	Fail	
Example<br>*Amoxicillin trihydrate-potassium Clavulnate*	*Augmentin*	*EP 078-001*	√		√	√		√					√	
Amoxicillin-Clavulanic acid														
Artemether-Lumefantrine														

Table B. Sample set medicine inspections.

**Inspection date:**		**Device name:**			**Inspector name:**		
Manufacturer	Study sample code	Trial 1	Trial 2	Trial 3	**Overall classification**		Comment
					Pass	Fail	
KPN	***SPS-01***	***PASS***			√		
KPN	***SPS-02***	***FAIL***	***FAIL***			√	
KPN	***SPS-03***	***FAIL***	***PASS***	***FAIL***		√	
