# Supplementary material for: A comparative field evaluation of six medicine quality screening devices in Laos
Source: PLoS Negl Trop Dis. 2021 Sep 30;15(9):e0009674. doi: 10.1371/journal.pntd.0009674 (PMC8483322; doi:10.1371/journal.pntd.0009674)
Supplement: S5 Table — Time and motion study observer recording sheet in a) Evaluation Pharmacy inspection b) Sample Set of medicines inspection. Note that record sheets were adapted for the Paper Analytical Cards. Table A. Evaluation pharmacy inspection. Table B. Sample set inspections. (PDF) [file pntd.0009674.s010.pdf]

S5 Table. Time and motion study observer recording sheet in A Evaluation Pharmacy inspection B Sample Set of medicines inspection. Note that record sheets were adapted for the Paper Analytical Cards

Table A. Evaluation Pharmacy Inspection

Operation description	Details:	Observer:	
	Inspector:	Time of inspection:	
	Date of inspection:	**Instrument set-up time**	
		**Calibration time**	

**Phase**	**Cycle**		**1**	**2**	**3**	**4**	**5**	**6**	**7**	**8**	**9**	**10**	**11**	**12**	**13**	**14**	**15**
Inspecting stock																	
	**API**		**ACA**	**ART**	**DHAP**	**AL**		**OFLO**				**SMTM**				**AZIT**	
Blisters inspected																	
		Brand name	Augmentin	Artesun IV	D-Artepp	CoArtem	Combi Art	Oflocee	Oflox acin	Ofloxin 200	Di-Flo	Vactrim	Strimside	Biseptrim	Sulfatrim	Oralzicin	Azithromax
Visual inspection	**Cycle**	1															
		2															
		3															
		4															
		5															
		6															
		7															
		8															
		9															
		10															
Tablets inspected																	
Sample testing	**Cycle**	1															
		2															
		3															
		4															
		5															
		6															

		7															
		8															
		9															
		10															
Interpreting/ Recording result and sample details	**Cycle**	1															
		2															
		3															
		4															
		5															
		6															
		7															
		8															
		9															
		10															

Table B. Sample Set Inspection

**Observer:**		**Inspector name:**							
**Sample set :**		**Date:**							
				Samples N°					
**Phase**				1	2	3	4	5	6
		**Sample code**							
Sampling		**Cycle**	1						
			2						
			3						
			4						
			5						
Device testing		**Cycle**	1						
			2						
			3						
			4						
			5						
Interpreting/Recording result and sample details		**Cycle**	1						
			2						
			3						
			4						
			5						
