# Supplementary material for: A comparative field evaluation of six medicine quality screening devices in Laos
Source: PLoS Negl Trop Dis. 2021 Sep 30;15(9):e0009674. doi: 10.1371/journal.pntd.0009674 (PMC8483322; doi:10.1371/journal.pntd.0009674)
Supplement: S6 Table — (PDF) [file pntd.0009674.s011.pdf]

S6 Table. Definition of the times measured in the evaluation pharmacy and sample set inspections

EP Task	Definition in Evaluation pharmacy inspection	SSM Task	Definition in sample set inspection	Notes
**Visual inspection of sample**	Starts when the inspector takes a look at secondary packaging or primary packaging when no secondary packaging is available to inspect the sample(s). Ends when the inspector brings his/her hand to the device.	**Not applicable***	Not applicable*	
**Sample testing**	Starts when the inspector is about to start using the device (touches device or removes tablet to begin testing). Ends when the device returns the result¥.	***Sampling***	Starts when the inspector starts to use the device (e.g.touches device, or removes tablet from packaging to begin testing). Ends before the process to obtain a result is started (e.g. ‘scan’ button is pressed; or PAD is put into the solvent)	
		Device Testing	Starts when the process to obtain a result is started (mainly press the button to process to analysis). Ends when the device returns the result.	For the PAD this starts when the papercard is placed into the water.
**Results interpretation and recording**	Starts when the results appear on the screen of the device. Ends when the pen is put back down from recording the result on the record sheet and the inspector begins one of the earlier phases again.	**Results interpretation and recording**	Starts when the results appear on the screen of the device. Ends when the pen is put back down from recording the result on the record sheet and the inspector begins one of the earlier phases again.	For the PAD this starts when the inspector starts to read the result of the card by comparison to the reference picture.

*not performed during the sample set inspections; ¥ Sampling and device testing times could not be accurately measured individually during the evaluation pharmacy inspections without interfering too much with the inspection, and were thus considered as one task
