# Supplementary material for: A comparative field evaluation of six medicine quality screening devices in Laos
Source: PLoS Negl Trop Dis. 2021 Sep 30;15(9):e0009674. doi: 10.1371/journal.pntd.0009674 (PMC8483322; doi:10.1371/journal.pntd.0009674)
Supplement: S7 Table — Time spent inspecting the evaluation pharmacy by phase–(A) Wilcoxon rank sum test results and (B) primary data. Table A. P-values of the Wilcoxon rank sum test (times are not normally distributed) results for the comparison between evaluation pharmacy inspection with specified device vs initial visual inspection are presented. Table B. Time spent inspecting evaluation pharmacy by phase—primary data. (PDF) [file pntd.0009674.s012.pdf]

S7 Table. Time spent inspecting the evaluation pharmacy by phase – (A) Wilcoxon rank sum test results and (B) primary data.

Table A. P-values of the Wilcoxon rank sum test (times are not normally distributed) results for the comparison between evaluation pharmacy inspection with specified device vs initial visual inspection are presented.

	**Set-up and calibration** Median (IQR) time (seconds)	**Visual inspection** Median (IQR) time (seconds)	**p-value[a]**	**Sampling /Device testing** Median (IQR) time (seconds)	**p-value[b]**	**Interpretation /Recording** Median (IQR) time (seconds)	**p-value[c]**	**Total** Median (IQR) time (seconds)	**p-value[d]**
**4500a FTIR**	194 (155-324)	448 (82-999)	0.061	2,696 (2,590 – 2,735)	0.002**	505 (387-875)	0.865	3,749 (3,471-4,770)	0.002**
**MicroPHAZIR RX**	729 (681-819)	178 (51-643)	0.016*	1,454 (1,315-2006)	0.027*	341 (229-589)	0.228	3,006 (2,575-3,454)	0.007**
**NIR-S-G1**	80 (67-85)	259 (0-565)	0.006**	1,098 (971-1,507)	0.050	311 (209-419)	0.022*	1,953 (1,487-2,133)	0.307
**PAD**	282 (86-661)	214 (0-657)	0.016*	4,289(3,542-6,718)	0.007**	947 (360-1,183)	0.315	5,600 (5,343-6,808)	0.007**
**Progeny**	373 (325-446)	297 (15-635)	0.006**	1,581 (1,178-2,134)	0.014*	868 (645-944)	0.008**	3,158 (2,438-3,842)	0.004**
**Truscan RM**	342 (186-452)	277 (0-796)	0.014*	1,576 (1,345-2,058)	0.017*	302 (214-342)	0.006**	2,500 (1,904-3,486)	0.011**
**Initial visual inspection**	N/A	994 (784-1,516)		N/A		522 (437-649)		1,516 (1,246-2013)	

[a] *P-value for time for visual inspection during evaluation pharmacy with specified device vs initial visual inspection time*

[b] *P-value for time spent for visual inspection+sampling/device testing vs initial visual inspection time*

[c] *P-value for time spent for interpretation/recording of results vs initial visual inspection interpretation/recording*

[d] *P-value for time spent for total time (set-up/calibration + visual inspection sampling/device testing+interpretation/recording) vs initial visual inspection time*

NB: One inspector did not perform the negative control of the PAD and one observer failed to record the calibration time with the MicroPHAZIR RX during one inspection. These data were thus excluded from the 'Set-up and calibration' and the 'Total time' data shown.

Table B. Time spent inspecting evaluation pharmacy by phase - primary data

Device	Inspector code	Training type	Set-up & calibration	Visual inspection	Sampling /Device testing	Interpretation/ Recording
NIR-S-G1	ADB-010	Intensive	85	0	1091.5	176
NIR-S-G1	ADB-013	Intensive	76	0	1641.5	453.5
NIR-S-G1	ADB-014	Rudimentary	64.5	580	931	314
NIR-S-G1	ADB_017	Rudimentary	84.5	518.8	1105.2	308.8
Progeny	ADB-03	Rudimentary	467	0	1147	586.5
Progeny	ADB-04	Intensive	312	668	1270	916
Progeny	ADB-013	Intensive	381	58	1892	819.5
Progeny	ADB-017	Rudimentary	364	536	2214	953
Truscan RM	ADB-010	Rudimentary	261.4	0	1284	191.3
Truscan RM	ADB-012	Intensive	422.5	876.5	2203	281.5
Truscan RM	ADB-014	Intensive	161.5	553	1529.5	348
Truscan RM	ADB-015	Rudimentary	462	0	1623	323
4500a FTIR	ADB-03	Intensive	365.5	0	2688.5	369
4500a FTIR	ADB-05	Intensive	145	327	2703	440
4500a FTIR	ADB-09	Rudimentary	186	569	2558	570
4500a FTIR	ADB-16	Rudimentary	201	1142	2746	977
PADs	ADB-010	Intensive	86	0	4288.5	711.5
PADs	ADB-13	Rudimentary	661	214	3542	1183
PADs	ADB-014	Rudimentary	281.5	656.5	6718	359.5
MicroPhazir RX	ADB_01	Intensive	819	643	1315	229
MicroPhazir RX	ADB_08	Rudimentary	729	51	1454	341
MicroPhazir RX	ADB_18	Intensive	681	178	2006	589
Initial visual inspection	ADB_01	N/A	0	970	0	255
Initial visual inspection	ADB_02	N/A	0	1302.5	0	762.5
Initial visual inspection	ADB_03	N/A	0	1017.3	0	445.7
Initial visual inspection	ADB_04	N/A	0	74.5	0	440.5
Initial visual inspection	ADB_05	N/A	0	713.5	0	328.5
Initial visual inspection	ADB_06	N/A	0	1628.5	0	706
Initial visual inspection	ADB_07	N/A	0	1030.3	0	528.3
Initial visual inspection	ADB_08	N/A	0	946	0	644.5
Initial visual inspection	ADB_09	N/A	0	1524.5	0	554
Initial visual inspection	ADB_10	N/A	0	903.5	0	350
Initial visual inspection	ADB_11	N/A	0	1550.5	0	444
Initial visual inspection	ADB_12	N/A	0	1518	0	477
Initial visual inspection	ADB_13	N/A	0	1515.5	0	803.5
Initial visual inspection	ADB_14	N/A	0	785.5	0	523.5
Initial visual inspection	ADB_15	N/A	0	464.5	0	521
Initial visual inspection	ADB_16	N/A	0	924.5	0	548
Initial visual inspection	ADB_17	N/A	0	780.5	0	663
Initial visual inspection	ADB_18	N/A	0	1307.5	0	425
