# Supplementary material for: A comparative field evaluation of six medicine quality screening devices in Laos
Source: PLoS Negl Trop Dis. 2021 Sep 30;15(9):e0009674. doi: 10.1371/journal.pntd.0009674 (PMC8483322; doi:10.1371/journal.pntd.0009674)
Supplement: S8 Table — Table B. Factors influencing the total time per sample [ln(total time)] in sample set testing—mixed effects generalised linear regression model (with inspectors and observers as random effects). (PDF) [file pntd.0009674.s013.pdf]

S8 Table. Median (IQR) times (seconds) per sample per device in sample set testing (A) and results of the mixed effects generalised linear regression model (B)

Table A. Median (IQR) sampling, device testing and recording times (seconds) per sample per device in sample set testing

P-values for comparison between devices of ln (sampling time), ln (device testing time) and ln (recording time) using mixed effects generalised linear regression models adjusted on device and training, and clustered by inspectors and observers

	**NIR-S-G1**	**MicroPHAZIR RX**	**Truscan RM**	**Progeny**	**4500a FTIR**	**PAD**	**Minilab**
**Median (IQR) sampling time (seconds)**	50 (32-67)	96 (71-128)	101 (59-136)	110 (73-138)	242 (179-320)	229 (192-262)	632 (529-947)
**MicroPHAZIR RX**	<0.001***	-	-	-	-	-	-
**Truscan RM**	<0.001***	0.981	-	-	-	-	-
**Progeny**	<0.001***	0.356	0.366	-	-	-	-
**4500a FTIR**	<0.001***	<0.001***	0.001**	<0.001***	-	-	-
**PAD**	<0.001***	<0.001***	<0.001***	<0.001***	0.059	-	-
**Minilab**	<0.001***	<0.001***	<0.001***	<0.001***	<0.001***	<0.001***	-
**Median (IQR) device testing time (seconds)**	21 (12-26)	8 (5-16)	20 (16-139)	87 (48-117)	10 (5-17)	329 (286-361)	1134 (797-1662)
**MicroPHAZIR RX**	<0.001***	-	-	-	-	-	-
**Truscan RM**	<0.001***	<0.001***	-	-	-	-	-
**Progeny**	<0.001***	<0.001***	0.001**	-	-	-	-
**4500a FTIR**	<0.001***	0.948	<0.001***	<0.001***	-	-	-
**PAD**	<0.001***	<0.001***	<0.001***	<0.001***	<0.001***	-	-
**Minilab**	<0.001***	<0.001***	<0.001***	<0.001***	<0.001***	<0.001***	-
**Median (IQR) interpreting/recording time (seconds)**	14 (9-23)	22 (14-32)	20 (11-43)	44 (36-69)	34 (25-74)	59 (25-121)	264.5 (0-387)
**MicroPHAZIR RX**	0.777	-	-	-	-	-	-
**Truscan RM**	<0.001***	0.051	-	-	-	-	-
**Progeny**	<0.001***	<0.001***	0.025*	-	-	-	-
**4500a FTIR**	<0.001***	<0.001***	0.029*	0.385	-	-	-
**PAD**	<0.001***	<0.001***	<0.001***	0.349	0.953	-	-
**Minilab**	<0.001***	<0.001***	<0.001***	<0.001***	<0.001***	<0.001***	-

* p<0.05, **p<0.01,***p<0.001

Table B. Factors influencing the total time per sample [ln(total time)] in sample set testing - mixed effects generalised linear regression model (with inspectors and observers as random effects)

Random effects	Variance	Standard deviation		
Inspector	0.02	0.12		
Observer	0.13	0.01		
**Fixed effects**	**Estimate**	**Standard error**	**Z**	**p-value**
Device				
NIR-S-G1	-	-	-	-
MicroPHAZIR RX	0.42	0.11	3.68	<0.001
Truscan RM	0.82	0.11	7.37	<0.001
Progeny	0.9	0.09	10.03	<0.001
4500a FTIR	1.21	0.11	10.5	<0.001
PAD	2.1	0.09	23.29	<0.001
Minilab	3.34	0.15	22.43	<0.001
Training				
Intensive	-	-	-	-
Rudimentary	0.09	0.06	1.61	0.11
Sample set				
AL	-	-	-	-
OFLO	-0.14	0.07	-1.91	0.06
SMTM	-0.12	0.09	-1.25	0.21

OFLO, ofloxacin; AL, artemether-lumefantrine; SMTM, sulfamethoxazole-trimethoprim
