# Supplementary material for: A comparative field evaluation of six medicine quality screening devices in Laos
Source: PLoS Negl Trop Dis. 2021 Sep 30;15(9):e0009674. doi: 10.1371/journal.pntd.0009674 (PMC8483322; doi:10.1371/journal.pntd.0009674)
Supplement: S9 Table — (PDF) [file pntd.0009674.s014.pdf]

S9 Table. Factors influencing the wrong classification of samples in sample set testing - mixed effects logistic regression (with inspectors as cluster-specific random effects)

Random effects	Odds-ratio	Standard error			
Inspector	0.27	1.4E-17			
**Fixed effects**	**Odds-ratio**	**Standard error**	**Z**	**95% CI**	**p-value**
Device					
NIR-S-G1	-	-	-	-	-
MicroPHAZIR RX	0.36	0.5	-0.74	0.03-5.28	0.54
Truscan RM	0.7	0.73	-0.34	0.09-5.43	0.74
Progeny	1.61	1.72	0.44	0.20-13.07	0.66
4500a FTIR	0.45	0.59	-0.61	0.04-5.82	0.54
PAD	1	1.72	0.44	0.14-6.98	0.66
Minilab	0.95	1.15	-0.04	0.09-10.23	0.97
Training					
Intensive	-	-	-	-	-
Rudimentary	1.51	0.91	0.69		0.49
Sample set					
AL	-	-	-	-	-
OFLO	0.6	0.36	-0.85	0.19-1.94	0.4
SMTM	1	-	-	-	-

OFLO, ofloxacin; AL, artemether-lumefantrine; SMTM, sulfamethoxazole-trimethoprim
