# Supplementary material for: A comparative field evaluation of six medicine quality screening devices in Laos
Source: PLoS Negl Trop Dis. 2021 Sep 30;15(9):e0009674. doi: 10.1371/journal.pntd.0009674 (PMC8483322; doi:10.1371/journal.pntd.0009674)
Supplement: Attachment — Submitted filename: PNTD-D-20-02193_reviewer-sp.pdf [file pntd.0009674.s015.pdf]

PLOS Neglected Tropical Diseases

A comparative field evaluation of six medicine quality screening devices in Laos

--Manuscript Draft--

**Manuscript Number:**	PNTD-D-20-02193
**Full Title:**	A comparative field evaluation of six medicine quality screening devices in Laos
**Short Title:**	Evaluation of six medicine quality screening devices in Laos
**Article Type:**	Research Article
**Keywords:**	Screening devices; medicine quality; substandard medicines, falsified medicines; post-market surveillance; Low- and Middle-Income countries
**Abstract:**	Background<br>Medicine quality screening devices hold great promise for post-market surveillance (PMS). However, there is little independent evidence on their field utility and usability to inform policy decisions. This pilot study in the Lao PDR tested six devices' utility and usability in detecting substandard and falsified medicines (SF).<br>Methodology/Principal findings<br>Observational time and motion studies of the inspections by 16 Lao medicine inspectors of 1) the stock of an Evaluation Pharmacy (EP), constructed to resemble a Lao pharmacy and 2) a sample set of medicines (SSM); were conducted without and with six devices: four handheld spectrometers (two infrared: MicroPHAZIR, NIR-S-G1 & two Raman: Progeny, Truscan); one portable infrared spectrometer (4500a), and single-use paper analytical devices (PAD). User experience was documented by interviews and focus group discussions.<br>Significantly more samples were wrongly categorised as pass/fail with the PAD compared to the other devices in EP inspections (p<0.05). The numbers of samples wrongly classified in EP inspections were significantly lower than in initial visual inspections without devices for 3/6 devices (NIR-S-G1, MicroPHAZIR, 4500a). The NIR-S-G1 had the fastest time per sample (median 93.5 sec, p<0.001). The time spent on EP visual inspection was significantly shorter when using a device than for inspections without devices, except with the 4500a, risking missing visual clues of SF presence. The main user errors were the selection of wrong spectrometer reference libraries and wrong user interpretation of PAD results. Limitations included repeated inspections of the EP by the same inspectors with different devices and limited availability of SF medicines.<br>Conclusions/Significance<br>This pilot study suggests policy makers wishing to implement portable screening devices in PMS should be aware that overconfidence in devices may cause harm by reducing inspectors' investment in visual inspection. It also provides insight into the advantages/limitations of diverse screening devices in the hands of end-users.
**Additional Information:**	
**Question**	**Response**
**Financial Disclosure**<br><br>Enter a financial disclosure statement that describes the sources of funding for the work included in this submission. Review the submission guidelines for detailed requirements. View published research articles from *PLOS NTDs* for specific examples.<br><br>This statement is required for submission and **will appear in the published article** if the submission is accepted. Please make	This work has been co-funded by the Regional Malaria and other Communicable Disease Threats Trust Fund, which has been co-financed by the Government of Australia (Department of Foreign Affairs and Trade); the Government of Canada (Department of Foreign Affairs, Trade and Development); and the Government of the United Kingdom (Department for International Development). The grant (RETA 8763) was managed by the Asian Development Bank (https://www.adb.org/) and awarded to PNN. A Wellcome Trust Grant N°202935/Z/16/Z (https://wellcome.org/) was also awarded to PNN. The funders had no role in study design, data collection and analysis, decision to publish, or preparation of the manuscript.

sure it is accurate.<br><br>**Unfunded studies**<br><br>Enter: *The author(s) received no specific funding for this work.*<br><br>**Funded studies**<br><br>Enter a statement with the following details:<br>• Initials of the authors who received each award<br>• Grant numbers awarded to each author<br>• The full name of each funder<br>• URL of each funder website<br>• Did the sponsors or funders play any role in the study design, data collection and analysis, decision to publish, or preparation of the manuscript?<br>• **NO** - Include this sentence at the end of your statement: *The funders had no role in study design, data collection and analysis, decision to publish, or preparation of the manuscript.*<br>• **YES** - Specify the role(s) played.<br><br>* typeset	
**Competing Interests**<br><br>Use the instructions below to enter a competing interest statement for this submission. On behalf of all authors, disclose any competing interests that could be perceived to bias this work—acknowledging all financial support and any other relevant financial or non-financial competing interests.<br><br>This statement **will appear in the published article** if the submission is accepted. Please make sure it is accurate. View published research articles from *PLOS NTDs* for specific examples.	The authors have declared that no competing interests exist.

**NO authors have competing interests**<br><br>Enter: *The authors have declared that no competing interests exist.*<br><br>**Authors with competing interests**<br><br>Enter competing interest details beginning with this statement:<br><br>*I have read the journal's policy and the authors of this manuscript have the following competing interests: [insert competing interests here]*<br><br>* typeset	
**Data Availability**<br><br>Authors are required to make all data underlying the findings described fully available, without restriction, and from the time of publication. PLOS allows rare exceptions to address legal and ethical concerns. See the PLOS Data Policy and FAQ for detailed information.<br><br>A Data Availability Statement describing where the data can be found is required at submission. Your answers to this question constitute the Data Availability Statement and **will be published in the article**, if accepted.	Yes - all data are fully available without restriction

**Important:** Stating 'data available on request from the author' is not sufficient. If your data are only available upon request, select 'No' for the first question and explain your exceptional situation in the text box.<br><br>Do the authors confirm that all data underlying the findings described in their manuscript are fully available without restriction?	
**Describe where the data may be found in full sentences. If you are copying our sample text, replace any instances of XXX with the appropriate details.**<br><br>• If the data are **held or will be held in a public repository**, include URLs, accession numbers or DOIs. If this information will only be available after acceptance, indicate this by ticking the box below. For example: *All XXX files are available from the XXX database (accession number(s) XXX, XXX.*).<br>• If the data are all contained **within the manuscript and/or Supporting Information files**, enter the following: *All relevant data are within the manuscript and its Supporting Information files.*<br>• If neither of these applies but you are able to provide **details of access elsewhere**, with or without limitations, please do so. For example:<br><br>*Data cannot be shared publicly because of [XXX]. Data are available from the XXX Institutional Data Access / Ethics Committee (contact via XXX) for researchers who meet the criteria for access to confidential data.*<br><br>*The data underlying the results presented in the study are available*	The data will only be available after acceptance.

from (include the name of the third party and contact information or URL).<br>• This text is appropriate if the data are owned by a third party and authors do not have permission to share the data.<br><br>* typeset	
Additional data availability information:	Tick here if the URLs/accession numbers/DOIs will be available only after acceptance of the manuscript for publication so that we can ensure their inclusion before publication.

Powered by Editorial Manager® and ProduXion Manager® from Aries Systems Corporation

Manuscript

Click here to access/download;Manuscript;Collection screening devices - Paper 3 Field Evaluation.pdf

Article 3 in Collection 'A multi-phase evaluation of portable screening devices to assess medicines quality for national Medicines Regulatory Authorities'

Title: A comparative field evaluation of six medicine quality screening devices in Laos

Short title: Evaluation of six medicine quality screening devices in Laos

Authors: Celine Caillet[1,2,3,5*], Serena Vickers[1,2,3]*, Stephen Zambrzycki[4], Facundo M. Fernández [4], Vayouly Vidhamaly[1,2,3], Kem Boutsamay[1,2,3], Phonepasith Boupha[1,2,3], Pimnara Peerawaranun[5], Mavuto Mukaka[2,5], Paul N. Newton[1,2,3,5]

Preferred degree: C Caillet PhD, S Vickers MB BChir, S Zambrzycki PhD candidate, FM Fernández Vasser-Woolley Professor, V Vidhamaly MSc, K Boutsamay PharmD, P Boupha PharmD, PN Newton FRCP.

Affiliation

[1] Lao-Oxford Mahosot Hospital Wellcome Trust Research Unit, Microbiology laboratory, Mahosot Hospital, Vientiane, Lao PDR.

[2] Centre for Tropical Medicine and Global Health, Nuffield Department of Medicine, University of Oxford, United Kingdom.

[3] Infectious Diseases Data Observatory (IDDO)/WorldWide Antimalarial Resistance Network (WWARN), University of Oxford, Oxford, United Kingdom.

[4] School of Chemistry and Biochemistry, Georgia Institute of Technology, Atlanta, Georgia, USA.

[5] Mahidol Oxford Tropical Medicine Research Unit (MORU), Faculty of Tropical Medicine, Mahidol University, Bangkok, Thailand.

*Joint first authorship

Abstract

Background

Medicine quality screening devices hold great promise for post-market surveillance (PMS). However, there is little independent evidence on their field utility and usability to inform policy decisions. This pilot study in the Lao PDR tested six devices' utility and usability in detecting substandard and falsified medicines (SF).

Methodology/Principal findings

Observational time and motion studies of the inspections by 16 Lao medicine inspectors of 1) the stock of an Evaluation Pharmacy (EP), constructed to resemble a Lao pharmacy and 2) a sample set of medicines (SSM); were conducted without and with six devices: four handheld spectrometers (two infrared: MicroPHAZIR, NIR-S-G1 & two Raman: Progeny, Truscan); one portable infrared spectrometer (4500a), and single-use paper analytical devices (PAD). User experience was documented by interviews and focus group discussions.

Significantly more samples were wrongly categorised as pass/fail with the PAD compared to the other devices in EP inspections ($p<0.05$). The numbers of samples wrongly classified in EP inspections were significantly lower than in initial visual inspections without devices for 3/6 devices (NIR-S-G1, MicroPHAZIR, 4500a). The NIR-S-G1 had the fastest time per sample (median 93.5 sec, $p<0.001$). The time spent on EP visual inspection was significantly shorter when using a device than for inspections without devices, except with the 4500a, risking missing visual clues of SF presence. The main user errors were the selection of wrong spectrometer reference libraries and wrong user interpretation of PAD results. Limitations included repeated inspections of the EP by the same inspectors with different devices and limited availability of SF medicines.

Conclusions/Significance

This pilot study suggests policy makers wishing to implement portable screening devices in PMS should be aware that overconfidence in devices may cause harm by reducing inspectors' investment in visual inspection. It also provides insight into the advantages/limitations of diverse screening devices in the hands of end-users.

Author summary

Substandard and falsified (SF) medicines threaten the lives of millions of people, especially where pharmaceutical legislation and regulation are limited. Screening for SF medicines in the market ('post-market surveillance') by medicine inspectors is crucial but currently relies only on subjective visual inspection of medicines in most countries. Many innovative portable screening technologies now exist and could be key additional assets to the current practice, but none have been extensively evaluated for medicine quality post-market surveillance. We assessed the utility and usability of six screening devices in the hands of Lao medicines inspectors in a pharmacy constructed to resemble a Lao pharmacy. Five spectrometers showed promising accuracies to identify falsified medicines, but difficulties to correctly set them up before running tests were observed. Reading and interpreting colour barcodes of the 'paper analytical cards'– a lab-on-a-chip test by inspectors were difficult, leading to lower accuracy than with spectrometers. The study suggests that overconfidence in devices may cause harm by reducing inspectors' investment in visual inspection - a crucial step to identify falsified medicines. Advantages/limitations of the devices are also documented to inform policy.

Background

According to a recent World Health Organization (WHO) report, ~10.5% of medical products circulating in low- and middle-income countries (LMICs) are either substandard or falsified (SF) [1]. Falsified medicines are the result of criminal activity, purporting to be genuine, authorized medicines, but are deliberately and fraudulently mislabelled with respect to identity and/or source [2].

Currently, national Medicines Regulatory Authorities (MRA) medicine inspectors in LMICs performing post-marketing surveillance (PMS) largely rely only on their own senses and knowledge to detect circulating SF [3]. A plethora of portable analysis screening tools have been developed over the last decade [4,5], allowing some degree of objective analysis of medicines in the ‘field’. However, there are enormous key gaps regarding the evidence-base to inform national MRAs of the optimal choice of device to detect SF medical products [4,6].

This is the third paper in the Collection ‘A multi-phase evaluation of portable screening devices to assess medicines quality for national Medicines Regulatory Authorities’ evaluating devices in Laos. Six devices deemed ‘field suitable’ in the laboratory evaluation phase were evaluated in the hands of medicine inspectors from the Lao Bureau of Food and Drug Inspection (BFDI) of the Ministry of Health [7]. Inspectors of medicines quality in Laos typically undertake routine inspection of pharmacies bi-annually, focusing on adherence to legislation and drug registration. Occasionally, medicines are purchased from a selection of pharmacies for screening using the Minilab [8]. All samples which fail Minilab screening, and a further 10% of those which pass, are sent to the Food and Drug Quality Control Center (FDQCC), Vientiane, for pharmacopeial testing.

We aimed to assess the utility and usability of six portable screening devices in the hands of Lao medicine inspectors for inspection in a simulated Evaluation Pharmacy.

Methods

Six devices and the Minilab, in line with its current use in Laos, were evaluated (**Table 1**). An outline of the different steps is given in **Fig 1.**

Fig 1. Outline of the field evaluation study.
EP: evaluation pharmacy; SSM: Sample set of medicines

Table 1. Main characteristics of the devices included in the study*

Device name	Manufacturer or Institution	Market status	Technology<br>Main Specifications	Handheld	Cost[a]
**4500a FTIR Single Reflection Spectrometer**	Agilent Technologies [9]	M	FTIR-MIR<br>Spectral range<br>$4000cm^{-1}$-$650cm^{-1}$	N	US$ 31,067
**Minilab[b]**	Global Pharma Health Fund E.V. [10]	M	TLC, disintegration test	N	US$ 2,510 (without reference standards)
**MicroPHAZIR RX analyser**	ThermoFisher Scientific [11]	M	FTIR - NIR<br>Wavelength range<br>1600nm-2400nm	Y	US$47,500
**NIR-S-G1 Spectrometer**	Young Green Energy - Innospectra[c] [12,13] (Global Good Fund developed the smartphone application)	M[d]	NIR - Dispersive<br>Wavelength range<br>900nm-1,700nm	Y	US$1,199 (without smartphone)
**Paper Analytical Device**	University of Notre-Dame [14] and Veripad [15] (Kenya, New-York and Boston)	D	Paper-based colour test	Y (S)	US$3
**Progeny Spectrometer**	Rigaku [16]	M	Raman<br>1064 nm laser	Y	(ex-demo model)
**TruScan RM Spectrometer**	ThermoFisher Scientific [17]	M	Raman<br>785 nm laser	Y	US$ 62,500 (including chemometric software package and tablet holder)

**Rapid Diagnostic Tests (RDT) and single-use immunoassay devices were deemed field-suitable in the laboratory evaluation work* [7], *but could not be evaluated in the present study because the developers of the single-use immunoassay test were unable to supply sufficient samples of the devices within the timeframe of the project.*

D, Under development ; FTIR, Fourier Transform Infrared ; M, Marketed ; MS, Mass spectrometry ; N, No ; NIR, Near infrared ; Y, Yes ; NIR, Near Infrared ; MIR, Mid-Infrared ; TLC, Thin-layer chromatography ; S, Single-use device.

a The costs reported here do not include VAT that may vary by country of purchase. Ordering several devices from the manufacturer is subject to potential reduced purchase cost.

b Unlike other devices, the Minilab was evaluated by laboratory technicians involved in current routine quality control at the Food and Drug Quality Control Center.

c At the time of the study the NIR unit was produced by Young Green energy. It is now produced by InnoSpectra Corporation.

d The near-infrared sampling unit is marketed, but the smartphone application is not.

Setting

An evaluation pharmacy (EP) was fashioned at Mahosot Hospital, Vientiane to resemble a Lao Class 2 pharmacy [18,19] and stocked with genuine and falsified field-collected medicines (FCM) stated to contain 41 different API or API combinations. The participants were asked to focus on inspections of medicines containing seven targeted API: ofloxacin (OFLO), sulfamethoxazole-trimethoprim (SMTM), azithromycin (AZITH), amoxicillin-clavulanic acid (ACA), artemether-lumefantrine (AL), artesunate (ART) (intravenous/intramuscular formulation) and dihydroartemisinin-piperaquine (DHAP). Genuine medicines were obtained from manufacturers and distributors in Laos and Thailand. Falsified versions of the antimalarial Coartem, containing none of the stated artemether or lumefantrine API, were provided by collaborators. Ultra-performance liquid chromatography (UPLC) was used as the reference technique to determine the amount of API(s) contained in FCM (**S1 Text**, **S2 Table**), except the falsified field-collected samples that had been tested by mass spectrometry [20].

Training medicine inspectors

Sixteen medicine inspectors, employees of the Vientiane Bureau for Food and Drug Inspection (BFDI, n=10) and the Vientiane district BFDI offices (n=6), volunteered for the study. They were randomised to receive either a ‘rudimentary’ verbal training with opportunity to practice use of the device on a few practice samples just prior the EP inspection; or an ‘intensive’ training including verbal presentation and substantial practice with the device, plus an additional rudimentary verbal training and practice just prior to the EP inspection. The trainings were given by Lao post-graduate research pharmacists, trained by the lead chemist overseeing the laboratory evaluation phase (**S3 Text**).

Evaluation pharmacy inspections

To refine the study protocol, EP inspections without devices were piloted by three pharmacy students from the Faculty of Pharmacy (University of Health Sciences, Vientiane) prior to the initial visual inspections.

Subsequently, four EP inspections by four different medicine inspectors were conducted per device, between September and December 2017. Each medicine inspector performed one simulated inspection without any device as a baseline (‘Initial visual inspection’), and up to three simulated EP medicine inspections with a device. The inspectors were randomly assigned to a combination of training and devices using the Excel RandBetweenrandom number generator. Constraints were that no inspector would test more than one handheld spectrometer (Progeny, MicroPHAZIR RX or Truscan RM) due to operating procedure similarity. Only inspectors from the district office would test the NIR-S-G1 because some Vientiane BFDI office inspectors had already used the NIR-S-G1 before (unpublished data).

All inspections were carried out independently by the participant working alone with one device per inspection. Inspectors were asked to collect any suspicious samples containing the targeted API assuming: no time limit, no budget restriction, that it was June 2015 (to avoid bias because some of the medicines in the EP had already expired by the evaluation) and that all blisters had no tablets missing (as some tablets were removed for analysis). They were encouraged to test samples through the blisters where appropriate. However, if they wished to perform testing requiring opening of primary packaging, the observer provided inspectors with already unpackaged samples (in a small zip-lock bag). If the inspector regarded all medicines as not suspicious at the end of the inspection, the inspector was asked to select a random sample of 10% of those which did not look suspicious or passed the device

test, for the Minilab testing. To reduce recall bias by medicine inspectors inspecting the EP several times, brands were changed between inspections and moved to different places.

Sample set inspections

After each EP inspection, a pre-determined 'sample set' of medicines (SSM) was tested by each inspector with the device in an office outside the EP. These SSMs tests facilitated direct comparisons between the devices for the time taken to test a single sample and to observe user errors. The samples consisted of FCM and 'simulated' samples made during the laboratory evaluation that were presented as single tablets, with packaging removed, in transparent zip-lock plastic bags labelled with the brand name, manufacturer, and dosage [7]. Three of six SSM samples of either AL, SMTM or OFLO were prepared to ensure that no inspector assessed each SSM more than once. However, eight FCM samples used for the creation of five spectrometer reference library entries and one used as a test sample (an artemether-lumefantrine sample), were subsequently found to be out-of-specification by UPLC analyses, rendering these reference libraries unreliable [7]. We thus discarded spectrometer results of testing of five samples included in SSM inspections (**Table 2**), and eight samples included in the EP inspections. No brands were discarded from the analysis for the PAD as they use reference colour codes pictures provided by the device developers.

Table 2. Samples sets of medicines initially included in sample set testing.

API	Study Code	Brand name	Quality type and origin of the medicines
**SMTM**	*G269/SPS20*	*Sulfatrim*	*G - Field-collected*
	G541/SPS21	*Sulfatrim*	*G - Field-collected*
	G558/SPS16	Diabeta 250	F - Look-alike[¥] - Field-collected
	SPS03	N/A*	100% API simulated medicine
	SPS04	N/A*	50% API simulated medicine
	SPS02	N/A*	0% API simulated medicine
**AL**	*MM17-01/SPS06*	*IPCA*	*G - Field-collected*
	SS0044/SPS07	*IPCA*	*F - Field-collected*
	G592/SPS22	*Coartem*	*G - field-collected*
	G593/SPS09	Coartem	G - Field-collected
	LC6/SPS10	Coartem	F - Field-collected
	LC10/SPS11	Coartem	F - field collected
**OFLO**	G569/SPS14	Oflocee	G - Field-collected
	G557/SPS15	Ofloxacin	G - Field-collected
	G555/SPS13	Di-Flo	G- Field-collected
	SPS05	N/A*	100% API - Simulated medicine
	SPS01	N/A*	50% API simulated medicine
	SPS02	N/A*	0% API simulated medicine

The test sample (G592/SPS22) and the reference library samples (n=4: G269/SPS20, G541/SPS21, MM17-01/SPS06, SS0044/SPS07) that were subsequently discarded from the results because of unexpected out-of-specifications API content as per UPLC analysis, are given in italics and highlighted. The samples with out-of-specifications reference library samples were still used for the PAD evaluation as the PAD reference libraries are independent reference pictures provided by the device developers.

G: genuine; F: falsified.

*Simulated sample; [¥] ‘look-alike’ medicines are defined as medicines stated as containing specific API (not one of the seven API included in this study) but the tablets were visually indistinguishable from genuine medicines in order to mimic a falsified medicine with a wrong API; the actual medicine was Diabeta® (chlorpropamide), but the tablets looked identical to Sulfatrim® (SMTM)] [21].

Baseline screening method: Minilab

Three laboratory technicians from the Food and Drug Quality Control Centre (FDQCC) of the Lao Ministry of Health (formally trained with the Minilab and involved in training of provincial inspectors), tested the samples selected as suspicious by medicine inspectors during the 16 initial visual inspections (without device), a random set of 10 % of the samples considered good quality during the 16 initial visual inspections, and the medicines of the three SSM, in line with the current use of the Minilab in Laos. Each inspector tested all samples of two or three API.

User satisfaction and focus group discussions

After completion of each SSM testing, the medicine inspectors were asked five open-ended questions, through face-to-face interviews in Lao language (**S4 Table**). Two months after the last EP inspection, three focus group discussions (FGD) were held. Each FGD had five medicine inspectors to give further insight into the utility and usability of the tested devices to support PMS systems (**S5 Table**).

Outcomes

In the absence of spectrometer manufacturer's guidelines, when a sample failed the first test with a device, medicine inspectors were instructed to operate a 'best of three' system for overall sample classification. Three tests were performed with the device on the failing samples, the most frequently occurring of 'pass' or 'fail' would then be the overall sample classification. For the PAD, inspectors were instructed to re-run failing samples once, as recommended by the developer. If the sample failed again, the sample was classified as failing. For both the EP and the SSM inspections, medicine inspectors were asked to record the sample identifier, pass/fail results of each single test, and the overall pass/fail classification on a recording sheet (**S6 Table**). Data analysis was performed using results from the inspector's overall pass/fail classification of the sample.

Time and motion studies were conducted by two observers (only one observer for the Minilab). In EP inspections, one observer unobtrusively with no conversation allowed with the participant, recorded the times to perform specific tasks on a recording sheet (**S7 Table**). Another observer recorded deviations from device protocol ('user errors'). For SSM, two observers recorded the times to perform specific tasks. The tasks recorded in EP were the times spent to conduct sample visual inspections, testing with the device ('sample testing'),

and interpret/record the results (**S8 Table)**. For SSM no visual inspection was conducted by the inspectors, as the tablets were provided outside their packaging. In addition to the time to interpret/record the results, two phases were identified as part of the SSM 'sample testing'; 'sampling' (started when use of the device or removal of the tablet from the packaging to begin testing started; ended when the process to obtain a result is started) and 'device testing' (started when the process to obtain a result is started; ended when result is obtained).

Data analysis

The median and interquartile range (IQR) number of samples wrongly classified, and the percentage with 95% confidence intervals (CI) of samples wrongly classified over all the EP inspections per device, are presented. Fisher's exact tests were used for the comparisons of the proportions of the number of samples wrongly classified by device pairs. Wilcoxon rank-sum tests were used to compare the number of samples wrongly categorised with and without devices in EP inspections. For SSM differences in accuracy in correctly classifying samples between devices were examined using mixed effects logistic regression yielding adjusted odds ratios, adjusted for training type (rudimentary/intensive), and sample set type as factors and inspectors as cluster-specific random effects.

The total time spent in EP inspection, time spent per phase during SSM, and total time spent per sample in SSM testing are described using medians (IQR). Wilcoxon rank-sum tests were used to test the differences in the times between the initial EP visual inspection and EP inspection for each device. For SSM testing, differences in the times between devices were examined using mixed effect generalised linear regression models to obtain the estimated devices' effect compared to the reference devices, adjusted for training group and sample set as factors and inspectors and observers as cluster specific random effects. The data

demonstrated skewed distributions for time and we therefore used the variable transformed to natural logarithm.

All tests were performed using a 5% (0.05) significance level. Microsoft Excel 2013 and STATA version 14.0 were used for analyses.

The user error(s) observed during EP and SSM inspections are summarized as narratives by category of errors (e.g. selection of the wrong reference library for spectrometers). The information of face-to-face interviews and the FGD are summarized and presented as narratives highlighting emerging common themes. More details by device are provided in **S9 Text**.

Results

Times results

Time to inspect the evaluation pharmacy

EP inspections with each device took significantly longer to complete compared with the initial visual inspections (25 min 16 s) without devices ($p<0.05$, Wilcoxon rank sum), except for the NIR-S-G1 (32 min 33 s, $p=0.307$)(**Fig 2, S11 Table)**. Visual inspection duration when using a device was significantly shorter for all devices than for initial visual inspections of the EP with no device, except for the 4500a FTIR ($p=0.061$). During one-third of the inspections with devices (n=11, 34%), inspectors spent less than one minute in sample visual inspection (data not shown). As one inspector did not perform the negative control of the PAD and the observers failed to record the calibration time with the MicroPHAZIR RX during one inspection, these data were excluded from the analyses.

Fig 2. Time spent inspecting evaluation pharmacy, by device

Values in the figure are medians (IQR); 2,000 seconds is ~ 33 minutes and 8,000 seconds is about 2 hours

Time per sample in sample set inspections

For SSM inspections, the median time to test one sample ranged from 94 sec for the NIR-S-G1 to 2,063 sec (34 min 23 s) for the Minilab. The Minilab and PAD took significantly longer total times per sample compared to other devices ($p<0.001$)(**Table 3**).

Table 3. Pairwise comparisons of the median total time taken per sample in sample set testing

	4500a FTIR	MicroPHAZIR RX	Minilab	NIR-S-G1	PAD	Progeny	Truscan RM
**4500a FTIR**	-	<0.001	<0.001	<0.001	<0.001	0.004	0.009
**MicroPHAZIR RX**	-	-	<0.001	<0.001	<0.001	<0.001	0.002
**Minilab**	-	-	-	<0.001	<0.001	<0.001	<0.001
**NIR-S-G1**	-	-	-	-	<0.001	<0.001	<0.001
**PAD**	-	-	-	-	-	<0.001	<0.001
**Progeny**	-	-	-	-	-	-	0.51
**Truscan RM**	-	-	-	-	-	-	-
**Median total time per sample (IQR)/secs**	316 (206-373)	134 (98-170)	2,063 (1,766-2,920)	94 (61-112)	620 (562-716)	273 (163-302)	148 (109-299)

P-values of the mixed effects generalised linear regression model of ln(total time) adjusted by device and training, and clustered by inspectors and observers

The NIR-S-G1 had a significantly shorter total time per sample (median 94 s) than any other devices tested ($p<0.001$); sampling was significantly faster than for the other devices and in interpreting/recording compared to all the devices except the MicroPHAZIR RX (median of 14 s vs 22 s, $p=0.78$) (**S10 Table**). The MicroPHAZIR RX was significantly faster in testing one sample than all other devices except the NIR-S-G1. The Progeny was significantly slower in device testing and interpreting/recording times per sample than the MicroPHAZIR RX and the Truscan RM ($p<0.001$). The PAD and 4500a FTIR sampling times were not significantly different (4 min 2 s and 3 min 49 s, respectively, $p=0.059$).

The inspectors with rudimentary training did not spend longer testing one sample, compared to the inspectors with intensive training, adjusted for devices, sample set tested, and clustered by inspectors and observers (p=0.11, data not shown).

Device accuracy

Over all EP inspections, samples were wrongly categorized with a frequency of 0% with the Truscan RM and MicroPHAZIR RX, to 37.9% (95% CI: 20.7-57.7%) with the PAD (**Table 4**). Significantly more samples were wrongly classified with the PAD compared to all other devices (p<0.05). All incorrect classification results were for genuine medicines being classified as suspicious (false positive).

Table 4. Pairwise comparisons of the percentage of samples wrongly classified over all inspections out of total samples tested overall with the devices in the evaluation pharmacy inspections

	4500a FTIR	MicroPHAZIR RX	NIR-S-G1	PAD	Progeny[b]	Truscan RM[b]
**4500a FTIR**	-	0.103	1.000	0.014*	1.000	0.242
**MicroPHAZIR RX**	-	-	0.243	<0.001***	0.167	N/A[a]
**NIR-S-G1**	-	-	-	0.005**	1.000	0.269
**PAD**	-	-	-	-	0.023*	<0.001***
**Progeny**	-	-	-	-	-	0.225
**Truscan RM**	-	-	-	-	-	-
**% samples wrongly classified (95% CI)**	9.7 (2.0-25.8)	0 (0-10.3)	7.7 (1.6-20.9)	37.9 (20.7-57.7)	8.3 (1.0-27.0)	0 (0-13.2)

P-values of the Fisher's exact test are presented

* p<0.05, **p<0.01,***p<0.001

a Not applicable as no samples were wrongly categorised in inspections with the Truscan RM or MicroPHAZIR RX

b Artesunate samples were discarded from the results analysis because samples were scanned through the glass vials by the inspectors, although reference library was created by scanning through a replacement packaging (plastic packaging)

The median numbers of samples wrongly classified in EP inspections with the 4500a FTIR [1 (0.3-1)], MicroPHAZIR RX [0 (0-0)], NIR-S-G1 [1 (0.3-1)] and Truscan RM [0 (0-0)] were significantly lower than in initial visual inspections [p=0.048, p=0.008, p=0.048 and p=0.005, respectively] (**Table 5**). There were no statistical differences in the number of samples wrongly classified in EP inspections with the PAD [2 (1-5.3)] and Progeny [0 (0-1.5)], compared to initial visual inspections (p=0.631 and p=0.059, respectively).

Table 5. Comparison of the number of samples incorrectly classified in evaluation pharmacy inspections with devices vs initial visual inspection without device

Device	Z	p-value	Median (IQR) number of samples wrongly classified with the device	Median (IQR) number of samples wrongly classified in initial inspection$^{\$}$
**4500a FTIR**	-1.980	0.048*	1.0 (0.3-1.0)	2.0 (1.0-2.3)
**MicroPHAZIR RX**	2.638	0.008**	0 (0-0)	2.0 (1.0-2.3)
**NIR-S-G1**	1.980	0.048*	1.0 (0.3-1.0)	2.0 (1.0-2.3)
**PAD**	-0.480	0.631	2.0 (1.0-5.3)	2.0 (0.8-2.3)
**Progeny**	1.891	0.059	0 (0-1.5)	1.5 (1.0-2.3)
**Truscan RM**	2.814	0.005**	0 (0-0)	1.5 (1.0-2.3)

Z statistic and p-value of the Wilcoxon rank sum test are presented

*** p<0.05, **p<0.01,***p<0.001**

$ The numbers of samples wrongly categorized in initial inspections without devices used in the comparisons vary because we included only brands tested in initial inspections that each device were able to test (e.g. AL samples wrongly categorized during initial inspections were excluded for the PAD, as the PAD could not test samples containing AL). In both initial inspections without devices and inspections with devices, we excluded samples wrongly categorized from brands subsequently found to have reference library spectra obtained from poor quality reference samples (as per UPLC analyses), except for the PAD.

For SSM inspections there were no significant differences between devices that wrongly classified samples as suspicious or not suspicious, adjusted by training status, sample set tested, and clustered by inspectors (**Table 6**).

Table 6. Matrix of pairwise comparisons of accuracy of devices in classifying samples incorrectly during sample set inspections (test device in row vs reference device in column)

	4500a FTIR	MicroPHAZIR RX	Minilab	NIR-S-G1	PAD	Progeny	Truscan RM
**4500a FTIR**	-	1.2 (0.1-25.0)	0.5 (0.0-7.5)	0.5 (0.0-5.8)	0.5 (0.0-4.9)	0.3 (0.0-3.5)	0.6 (0.1-7.5)
**MicroPHAZIR RX**	-	-	0.4 (0.0-6.5)	0.4 (0.0-5.3)	0.4 (0.0-3.8)	0.2 (0.0-2.8)	0.5 (0.0-6.1)
**Minilab**	-	-	-	0.9 (0.1-10.2)	0.9 (0.1-6.5)	0.6 (0.1-4.2)	1.3 (0.2-10.8)
**NIR-S-G1**	-	-	-	-	1.0 (0.1-6.9)	0.6 (0.1-3.0)	1.4 (0.2-10.9)
**PAD**	-	-	-	-	-	0.4 (0.1-2.5)	1.4 (0.3-7.0)
**Progeny**	-	-	-	-	-	-	2.3 (0.4-13.0)
**% samples wrongly classified (95%CI)[$]**	5.6 (0.1-27.3)	7.7 (0.2-36.0)	11.8 (1.5-36.4)	11.1 (1.4-34.7)	20.8 (7.1-42.2)	23.5 (6.8-49.9)	15.0 (3.2-37.9)

Odds ratio (95% CI) of the mixed effect logit model, with adjustment on the type of training received (rudimentary or intensive), sample set type (OFLO, AL, SMTM), and clustered by inspectors, are presented

$95%CI for binomial distribution

Over all SSM inspections, 10 out of 18 (55.6%) misclassifications were false negative results with samples containing 50% of OFLO or SMTM, two (11.1%) were false negative falsified FCM stated to contain AL and six (33.3%) were false positive samples of OFLO and SMTM.

The two 50% API samples tested with both the MicroPHAZIR RX and NIR-S-G1 were correctly classified as suspicious whereas the 4500a FTIR correctly classified 1/2, the Minilab 0/2, the PAD 3/4, the Progeny 0/3, and the Truscan RM 1/4.

Inspectors with rudimentary training were not significantly more likely to wrongly classify the samples compared to those with intensive training in SSM [OR 1.5 (95% CI 0.5-4.9)] adjusted by devices and sample set tested and clustered by inspectors (data not shown).

Observed user errors

The main observed user errors with the MicroPHAZIR RX, NIR-S-G1, Progeny, and Truscan RM were the selection of wrong comparator reference libraries in 3.9%, 26.6%, 7.5% and 20.0% of the scans in EP inspections and 0.0%, 27.8%, 0.0% and 25.8% in SSM inspections, respectively (**Table 7**). The inspectors recognized their errors and repeated the tests during MicroPHAZIR RX and Progeny inspections, resulting in no overall

misclassification of samples. However, in 16 of 17 scans (88.9%) using wrong reference libraries with the Truscan RM and 21/27 (77.8%) using the NIR-S-G1, the users did not realize the errors. In all these cases, the wrong brand (a different brand stated to contain the same API and strength) was selected by the users as the reference library. None of the 11 samples scanned with wrong reference libraries for which the users did not recognize the errors were misclassified with the Truscan RM. However, four samples out of the 17 samples tested with wrong reference library with the NIR-S-G1, for which the users did not recognize the errors, were incorrectly classified (all were false positives). Two out of 29 samples tested (6.9%), and four out of 24 samples tested (16.7%) were misclassified as a result of errors in PAD interpretation in EP and SSM inspections, respectively.

Table 7. Observed user errors during EP and SSM inspections

Device	Selection of wrong reference libraries in EP			Selection of wrong reference libraries in SSM			Other errors		
	Scans % (n/N)	Samples % (n/N)	% (n/N) of samples misclassified (N=total number tested)	Scans % (n/N)	Samples % (n/N)	% (n/N) of samples misclassified (N=total number tested)	Description	% (n/N) of samples misclassified (N=total number tested)	Comments
**4500a FTIR**	N/A	N/A	N/A	N/A	N/A	N/A	7.9% (3/51) scans in EP and 3.0% (1/33) in SSM were not renamed after acquisition in the device memory	0	Samples were recorded on paper by the inspectors. Thus errors did not result in sample misclassification, but could affect traceability in practice
**MicroPHAZIR RX***	3.9% (2/51)	5.9% (2/34)	0.0% (0/34)	0.0% (0/33)	0.0% (0/13)	0.0% (0/13)$	5.9% (3/51 scans with tablets not inserted in sample cover) in EP	0$	All errors made by inspector with rudimentary training
**NIR-S-G1**	26.6% (17/63)	28.2% (11/39)	5.1% (2/39)	27.8% (10/36)	33.3% (6/18)	11.1% (2/18)	None	None	
**PAD**	N/A	N/A	N/A	N/A	N/A	N/A	Reading result errors in 24.1% (7/29) samples tested in EP and 20.8% (5/24) in SSM	6.9% (2/29) in EP and 16.7% (4/24) in SSM	In some cases both the PAD showed wrong colours and the user made an error of interpretation, leading to overall correct classification (more details in S9 TextS9 Text. Results of the evaluation by device)
							None of the failing samples were rerun despite clear instructions to rerun suspicious samples	Uncertain	
							Use of the same visibly contaminated water for multiple PAD during one EP inspection (inspector with rudimentary training)	Uncertain	
**Progeny¥**	7.5% (4/53)	13.3% (4/30)	0.0% (0/30)$	0.0% (0/21)	0.0% (0/13)	0.0%$	Deviation from study protocol: One inspector did not run the 'Application' test after running the 'Analyse' function	0	
**Truscan RM**	20.0% (9/45)	19.4% (6/31)	0.0% (0/31)	25.8% (8/31)	20.0% (5/20)	0.0%	None	None	Inspectors did not recognize they selected the wrong library entry, but the device returned correct result

*3 inspections only with the MicroPHAZIR RX because results of one inspection were discarded because of an issue over the inbuilt reference library; $Errors were recognized by the inspectors who re-tested the samples without mistakes; ¥ Wrong selection of the reference library can happen only with the 'Application' function. Using the analyse function, no user errors were observed during both EP and SSM

User satisfaction

All spectrometers, except the NIR-S-G1, were felt to be heavy and/or rather cumbersome. The portable 4500a FTIR spectrometer was perceived as suitable in inspections of manufacturing and distributing sites by inspectors who liked the extra information given by the table of results (table of matches, with its list of API and % match) as this was felt to increase confidence in the device results. However, the 4500a was identified as not suitable for routine pharmacy inspection due to its large size and the need for sample crushing and for cleaning the sampling window : ***"[…] in most of the big pharmacies in our country there's no space to test, people queue for hours to get their medicines; there's no way to place the heavy device like this and computer and if we want to test it's just rarely possible.***"

The MicroPHAZIR RX was described as easy to use, reliable, comfortable, and fast. The sample window indicator, which shows the inspector whether the sample is sufficiently covered by the sample window to produce a reliable result, was cited as a helpful additional feature giving inspectors additional confidence in their sampling technique. The device froze during one of the four EP inspection and all the records were lost, which made that inspector think that the use of the device would be '*wasting of time*'.

The NIR-S-G1 was singled out by medicine inspectors as well-suited for any level of the supply chain due to its small size, fast testing time, and easy-to-use smartphone application. However, the lack of capability to create and update the reference library of comparators locally was perceived as a key limitation.

Although medicine inspectors liked the PAD lack of reliance on electricity or sophisticated instrumentation, the need to prepare samples, to have a working space to carry out the analysis,

and the long experiment time were frequently raised as concerns with regards to their usability in pharmacies and at distributors sites. Difficulties in interpreting the results were often highlighted : ***"[…] for example in the protocol it's said it's pink and in reality it's a faded pink so it depends on the user's eyesight and his/her decision. So, it's difficult to tell the actual color."*** Two medicine inspectors acknowledged that the PAD would be useful to test raw materials at manufacturers.

Medicine inspectors liked the ability of the Progeny to display more than a 'pass' or 'fail' result. It was felt to be quite slow to scan, and three inspectors commented that the touchscreen was not very responsive. Three inspectors stated that the supplied tablet holder was difficult to use with small tablets. It was noted as interesting for inspections in manufacturers, distributors, or border points, but of limited use in pharmacy outlets.

The Truscan RM was perceived as easy and comfortable to use, but with a slow device testing time (this was raised during face-to-face interviews). It was deemed more suitable for use in inspection of manufacturers' plants, distributors, or border check points rather than in pharmacy outlets.

Discussion

This pilot study provides insight into the performances, the advantages/limitations of six screening devices in the hands of Lao medicines inspectors in simulated inspections. The NIR-S-G1 was the fastest spectrometer to test one sample whilst the PAD and the Minilab took significantly longer to test one sample than the all the spectrometers. Within the limited context of this study, the five spectrometers showed promising accuracies to identify falsified and genuine medicines. Inspectors difficulties to read and interpret colour barcodes with the single-use PAD may have led to a lower accuracy of these compared to other devices in the evaluation pharmacy. The selection of the wrong reference libraries by the inspector was observed with all spectrometers but these errors did not lead to final erroneous classifications of medicines, except in four cases with the NIR-S-G1. Findings also suggest that policy makers wishing to implement devices in PMS should be aware that overconfidence in devices may cause harm by reducing inspectors' visual inspection investment.

Of the six devices studied, five were spectrometers and there were no significant differences in the performance outcome measured in EP or SSM inspections between them in our limited data set. The inspections of the EP with all spectrometers except the Progeny were more accurate than the initial visual inspection with no device. Most spectrometers, except for the NIR-S-G1, were felt too heavy and cumbersome for pharmacy inspections. In one inspection, the MicroPHAZIR RX was used with the device resting on the bench of the simulated pharmacy rather than as a 'handheld' device. The handheld NIR-S-G1 was perceived as light, handy and user-friendly, thus suitable for routine inspections at any level of the supply chain.

The Truscan RM, Progeny (using the 'application' function), MicroPHAZIR RX and the NIR-S-G1 all gave a simple 'pass/fail' result, a feature appreciated by the medicine inspectors.

The ‘matching’ values given by the Progeny (using the ‘analyse’ function) and the 4500a FTIR gave reassurance in the results. Except the 4500a FTIR, the spectrometers require the user to select the correct reference library entry for comparison with the tested spectrum. Out of the all samples tested with the NIR-S-G1 with the inspector selecting the wrong reference library, almost one-fourth gave false classifications. In instances where the wrong reference libraries were selected with the TruScan RM, the Progeny, or the MicroPHAZIR RX, overall classification of the sample as suspicious or not suspicious by the inspector were not compromised. Indeed, with the MicroPHAZIR RX and Progeny, the mistakes were all recognised by the inspectors who repeated the analysis. In all cases with the Truscan RM, the library selection errors were not recognized by the inspectors but the device gave the correct result and the samples were accurately classified overall. There appeared to be a lack of awareness that different brands of the same API may contain different excipients, resulting in need for different reference libraries. Indeed, in all the cases the wrong ‘brands’ library entries selected by the inspectors were that of medicines containing the same API(s) at the same strength than the tested medicine, such as selecting Sulfatrim instead of Vactrim (both containing SMTM). In some instances, the result shown by the device was that expected if the correct library had been selected. For example, in the case of Sulfatrim vs. Vactrim, the device gave a ‘pass’ to the Vactrim tablet being tested against the Sulfatrim reference library. It is likely that in some cases different brands containing similar API and excipient compositions lead to a correct overall classification of the samples as suspicious or not because the medicines’ are chemically similar. Although little is known about the variability of response of portable spectrometers to different brands of the same API(s), our findings suggest that the Raman devices may be less susceptible to formulation-specific signature variations than the NIR [22]. Improvement of the function to select the reference library in the NIR-S-G1 and using the in-built

barcode reader (featured in the Truscan RM, MicroPHAZIR RX, and Progeny) are likely to reduce the risk of wrong library selection and thus the number of false results.

The MicroPHAZIR RX and the NIR-S-G1 correctly classified all 50% API medicines tested, whereas the other spectrometers correctly classified only none to less than half of them. Identification of substandard samples containing between 70 and 90% of API with spectrometers, and those containing higher than the upper limit of the specifications-commonly found in field surveys [23,24], should be further explored.

The PAD required the user to make subjective judgement on the visual likeness of the test sample result to the reference result. This is likely to have contributed to the significantly higher number of samples wrongly categorised by the medicine inspectors as compared to other devices in the EP inspections. Problems with colour interpretation were also observed for the Artemisinin derivative test (ADT), despite the high level of confidence in the results expressed by the laboratory technicians who were newly trained to use the ADT [25]. These and issues of colour blindness are likely to be greatly helped by automated smartphone interpretation software with image analysis software, such as ImageJ [26–28]. In addition, although the inspectors were told to change the water used for the PAD before running a new sample if water contamination occurred, one inspector, with rudimentary training, did not. This supports the impression that the training given may have been insufficient. For the advantages of the PAD to be realised, training schemes with user proficiency testing, continuing education, certification and quality control will be necessary. Interestingly, the PAD identified three out of four 50% API samples tested. Expert readers had previously identified 19/20 samples containing 40% of the stated amount of chloroquine as giving a ‘weak’ signal, compared to the 18/18 full-strength formulations giving a ‘strong’ signal, but mixed results were observed in identification of samples with 70% stated API

[29]. The PAD deserve more consideration as devices for semi-quantitative detection of substandard antimicrobials as they are probably important drivers of antimicrobial resistance. Indeed, samples containing low ceftriaxone concentrations were successfully identified with high sensitivity by a novice PAD user and ImageJ, showing promising results for identification of substandard samples containing less than 80% of the stated API [28]. Recently, the µPAD, a competitive enzyme assay on paper, has shown encouraging results in the hands of five users unfamiliar with the device, to identify falsified beta-lactams [30]. The semi-quantitative properties of the ChemoPADs, using similar features to the PAD, coupled with an image analyser identified 11 of 20 substandard cisplatin samples found in Ethiopia [27].

As expected, devices requiring sample preparation and user data interpretation (4500a FTIR, PAD, Minilab) took the inspectors significantly longer time per sample than those which do not. This was particularly pronounced for both the PAD and the Minilab, but this may be offset by their ability to run more than one sample concurrently. The NIR-S-G1 and MicroPHAZIR RX were the fastest devices to test individual samples. At the time of the study, the NIR-S-G1 did not have the ability to record sample details on the device and did not have a sample holder for unpackaged tablets. This contributed to its fast speed of analysis, but there may be inconsistency with tablets that are too small or do not fit flush against the sampling window.

The time spent on visual inspection in the EP was significantly shorter when using a device than for initial visual inspections alone, except for the 4500a FTIR ($p=0.061$). The selection of suspicious samples by visual inspection may be key to identify poor quality medicines, especially those with obvious defects such as discoloration or typographic errors [31]. Therefore, reduction in visual inspection time may have negative consequences in finding suspicious medicines samples. Hence, it is possible that device introduction could be counterproductive depending on

the prevalence of SF medicines that could be visually recognised. Instead of visually inspecting different blisters of the same brand as in the inspection without device, it seems that the inspectors chose to test a sample of one packet of a brand with the device, taking that result to be representative of all samples from that brand. This may be an artefact of the experimental set-up as the EP was inspected without devices first. When questioned about why they chose not to do visual inspection, some inspectors replied that they would expect samples of the same brand of medicine in the same pharmacy to be from the same batch, hence of identical quality. The paucity of visual inspection of samples could also be related to the increased perceived time pressure to complete an extra task within the 'normal' pharmacy inspection time. Further work is needed to investigate these findings and the impact of device use on real-life inspection effectiveness.

Non-destructive testing of samples is preferable for pharmacy inspections [6]. The lack of budget to buy medicines to test, and the waste of samples for the pharmacy being inspected were mentioned by medicine inspectors as pitfalls of destructive technologies. Even for non-destructive devices, testing can currently only be carried out through transparent packaging. Of the fourteen brands of the target EP medicines, ten were in opaque packaging and therefore had to be removed from the packaging (thus 'destroyed') prior to testing. Innovations to blister pack and packaging could facilitate accurate spectroscopic evaluation [4]. We have been unable to find discussion as to the impact of different plastics on spectral acquisition. Spatially offset Raman spectroscopy (SORS) technology deserves attention to scan through opaque packaging [32,33].

Few have discussed training requirements for device users [34–39], and limited scientific evidence can be retrieved from studies that were not primarily designed for that purpose. All the inspectors in our study were able to successfully complete pharmacy inspection and sample set testing with the devices regardless of the training they had received. In our limited data set,

inspectors with intensive training were not significantly more likely to correctly classify the samples as suspicious or not than those with less training.

Important limitations exist in our study and those, as well as the difficulties to perform such research encountered in our study are listed in **Box 1**.

Box 1. Limitations and difficulties encountered to perform the research study

- For the spectrometers, only one unit of each device was evaluated. We therefore make no assessment of variability between different units of the same device type.
- Only six API, all antimicrobials, and all sourced from one region were evaluated. One API (DHAP) of the seven initially selected for investigation had to be removed from analysis due to poor quality samples being used in construction of the device reference libraries.
- Only one parenteral formulation was investigated; all other samples were formulated as tablets. No testing of topical/liquid/capsule dosage forms was conducted.
- For laboratory-created spectrometer reference libraries (does not include NIR-S-G1, for which the developer created the reference library):
    - Manufacturer-set default values were used with no attempt to optimise these for specific medicines tested
    - Limited consideration of batch-to-batch variability for field-collected medicines
- Evaluation pharmacy included a small proportion of falsified medicines (3/~110 blisters stocked)
- Due to limited stock, some samples had exceeded their expiry date. Inspectors were specifically asked to overlook important normal cues for visual inspection (expiry date, inclusion on national list of registered medicines, condition of packaging, storage conditions). Overlooking these cues during inspection of the evaluation pharmacy has limited resemblance to the inspectors' standard practice.
- The field-study team did not receive any direct training from the manufacturer and followed protocols in a second language.
- The 4500a FTIR does not give a pass/fail result and requires interpretation. Bias may have been introduced in the measure and comparisons of effectiveness between the 4500a FTIR and other devices because of instructions given to the inspectors that were incorrect, due to a misunderstanding by the trainer (**S9 Text**).
- Whenever possible, the EP stock consisted of complete blisters, in original packaging. Medicines inspectors were encouraged to test samples through the blisters where appropriate. If needed, they were provided with already unpackaged samples. This was because of the limited number of samples available for the study in the EP, especially falsified medicines, and to preserve the complete blisters to avoid inspection bias introduced by progressively having more incomplete blisters stocked in the pharmacy.
- To avoid recall bias by medicine inspectors inspecting the EP several times, some of the brands stocked in the pharmacy were changed between inspections, samples were moved to different places, and the samples stocked in the pharmacy was thus not always consistent between inspections.
- Opinions from the inspectors were formed in the context of a 'routine' pharmacy inspection. Use of devices in different contexts, e.g. by manufacturers, or in a basic laboratory such as might be found at a provincial level, may have resulted in different user opinions.
- Samples of artesunate powder were scanned with the Truscan RM and Progeny through the glass vials by the inspectors, although the reference library was created by scanning through a replacement packaging (plastic packaging). These results were discarded from our analysis.
- Some of the samples were found to be poor quality by UPLC analysis, but results were not available until after completion of the study. As a result, we did not have access to good reference library comparators, and it was decided to discard results of the 13 affected samples.
- Number of inspections carried out with each device and the number of samples stocked, particularly of SF medicines, were limited.

As an independent public health investigation performed in a field setting, this study gives evidence to facilitate MRAs decisions as to whether these new technologies are appropriate for screening of diverse medicines in their countries. Without further objective validation, device implementation should be cautious. Their advantages, limitations, and cost-effectiveness [40] should be clearly understood and further investigated. However, the innovation of testing devices in an evaluation pharmacy holds promise for enhancing our understanding of their use between laboratory and real-life field scrutiny. With further work such devices hold great promise to empower medicine inspectors globally.

Authors contribution

SV, VV, KB, PB, SZ, PNN and CC were involved in the design of the methodology and the conceptualization. SV, VV, KB, PB, SZ and CC were involved in the investigation and data curation. SV, CC, PP and MM conducted the analysis and validation of the results. CC wrote the original draft. SV, PNN, FMF, and SZ reviewed and edited the manuscript. PNN organized the funding acquisition. SV, CC and PNN supervised the project and provided project administration support.

Acknowledgments

We are very grateful to the Government of the Lao PDR for their support, especially the Bureau of Food and Drug Inspection, the Food and Drug Department, the Food and Drug Quality Control Centre and the University of Health Sciences. We are grateful to Miss Viphavanh Soulaphy, Miss Orlathai Saiyasane, Miss Thipphaphone Keonakhone, Miss Sonethalee Senboutthalath, Miss Anousone Phengsombut, Miss Viengnakhone Thongphachanh, Miss Toutana Hormkinkeo, Miss Bouakham Saiyphimchai, Mr Amkha Senethavysouk, Mr Somboun

Nadonhai, Mr Xayasith Sengaroundeth, Miss Vilailad Phetlavanh, Miss Veosavanh Keovoravong, Miss Nongluck Xayyalath, Miss Maniphone Phimmaleen, Miss Anback Hongsivilay, Mr Lamngern Phodchanthonthavong who played vital roles in the project as inspectors in the Evaluation Pharmacy; Mr Somchai Chanthapany, Mr Sathaphone Bounmala, Mr Soulivong Souphanhthavong who conducted the Minilab analysis of the samples. We are very grateful to the Directors and staff of Mahosot Hospital for allowing us to install the Evaluation Pharmacy in the hospital grounds and to Assoc. Prof Mayfong Mayxay for his advice. We are grateful for the support of the Infectious Diseases Data Observatory and the Centre for Tropical Medicine & Global Health of Oxford University who invested in the administration of the project, and to the members of the Substandard and Falsified Medical Products Team of the World Health Organisation, Geneva. We are very grateful for the discussions with the manufacturers and developers of the devices and to Dr. Fred Behringer for conducting the UPLC analyses of the samples. We are very grateful for the technical assistance of Douglas Ball, Sonalini Khetrapal and Susann Roth.

References

1. World Health Organization. WHO Global Surveillance and Monitoring System for substandard and falsified medical products: executive summary. [Internet]. Geneva, Switzerland; 2017. Available from: WHO/EMP/RHT/SAV/2017.01

2. SF Medical Products Group, Essential Medicines and Health Products WHO. WHO Member State Mechanism on Substandard/Spurious/Falsely-Labelled/Falsified/Counterfeit (SSFFC) Medical Products. In: Seventieth World Health Assembly [Internet]. Geneva, Switzerland; 2017. p. A70/23: 33-36. Available from: http://www.who.int/medicines/regulation/ssffc/A70_23-en1.pdf?ua=1

3. Roth L, Nalim A, Turesson B, Krech L. Global landscape assessment of screening technologies for medicine quality assurance: stakeholder perceptions and practices from ten countries. Global Health [Internet]. 2018 Dec 25 [cited 2018 May 4];14(1):43. Available from: http://www.ncbi.nlm.nih.gov/pubmed/29695278

4. Vickers S, Bernier M, Zambrzycki S, Fernandez FM, Newton PN, Caillet C. Field detection devices for screening the quality of medicines: a systematic review. BMJ Glob Heal. 2018 Aug 29;3(4):e000725.

5. Kovacs S, Hawes SE, Maley SN, Mosites E, Wong L, Stergachis A, et al. Technologies for detecting falsified and substandard drugs in low and middle-income countries. PLoS One. 2014;9(3):e90601/1-e90601/11, 11 pp.

6. Roth L, Nalim A, Turesson B, Krech L. Global landscape assessment of screening technologies for medicine quality assurance: stakeholder perceptions and practices from ten countries. Global Health. 2018 Dec 25;14(1):43.

7. Zambrzycki SC, Caillet C, Vickers S, Bouza M, Donndelinger D V., Winalski LC, et al. Laboratory evaluation of twelve portable devices for medicine quality screening. PLoS Negl Trop Dis. ,submitted.

8. Global Pharma Health Fund. The GPHF-Minilab™ - Fact Sheet [Internet]. GPHF - Global Pharma Health Fund. 2016 [cited 2020 Dec 10]. Available from: http://www.gphf.org/minilab/factsheet.htm

9. Agilent. FTIR Compact & Portable Systems - 4500 Series Portable FTIR [Internet]. [cited 2020 Dec 10]. Available from: https://www.agilent.com/en/products/ftir/ftir-compact-portable-systems/4500-series-portable-ftir

10. Global Pharma Health Fund. GPHF | The GPHF-Minilab™ [Internet]. [cited 2020 Dec 10]. Available from: https://www.gphf.org/en/minilab/

11. ThermoFisher Scientific. microPHAZIR™ RX Analyzer [Internet]. [cited 2020 Dec 1]. Available from: https://www.thermofisher.com/order/catalog/product/MICROPHAZIRRX?SID=srch-srp-MICROPHAZIRRX

12. Young Green Energy Co. Young Green Energy Co. [Internet]. [cited 2020 Dec 1].

Available from: http://www.young-green.com/en/home

13. InnoSpectra Corporation. Reflective Mode - Spec [Internet]. [cited 2020 Dec 10]. Available from: http://www.inno-spectra.com/en/product

14. University of Notre-Dame. Paper Analytical Device Project - University of Notre Dame [Internet]. [cited 2020 Dec 10]. Available from: https://padproject.nd.edu/

15. VERIPAD. Product - The Veripad Solution - Counterfeit Medicines Detection Device [Internet]. [cited 2020 Dec 10]. Available from: https://www.f6s.com/veripad

16. Rigaku. Handheld Raman | Progeny [Internet]. [cited 2020 Dec 10]. Available from: https://www.rigaku.com/products/raman/progeny

17. ThermoFisher Scientific. TruScan^{TM} RM Handheld Raman Analyzer [Internet]. [cited 2020 Dec 10]. Available from: https://www.thermofisher.com/order/catalog/product/TRUTOOLS?SID=srch-srp-TRUTOOLS

18. Caillet C, Sichanh C, Syhakhang L, Delpierre C, Manithip C, Mayxay M, et al. Population awareness of risks related to medicinal product use in Vientiane Capital, Lao PDR: a cross-sectional study for public health improvement in low and middle income countries. BMC Public Health [Internet]. 2015;15:590. Available from: http://www.ncbi.nlm.nih.gov/pubmed/26116373

19. Syhakhang L, Lundborg CS, Lindgren B, Tomson G. The quality of drugs in private pharmacies in Lao PDR: a repeat study in 1997 and 1999. Pharm World Sci PWS. 2004 Mar 1;26(6):333–8.

20. Bernier MC, Li F, Musselman B, Newton PN, Fernandez FM. Fingerprinting of falsified artemisinin combination therapies via direct analysis in real time coupled to a compact single quadrupole mass spectrometer. Anal Methods. 2016;8(36):6616–24.

21. Caillet C, Sichanh C, Assemat G, Malet-Martino M, Sommet A, Bagheri H, et al. Role of Medicines of Unknown Identity in Adverse Drug Reaction-Related Hospitalizations in Developing Countries: Evidence from a Cross-Sectional Study in a Teaching Hospital in the Lao People's Democratic Republic. Drug Saf. 2017 Sep 20;40(9):809–21.

22. Ciza PH, Sacre P-Y, Waffo C, Coïc L, Avohou H, Mbinze JK, et al. Comparing the qualitative performances of handheld NIR and Raman spectrophotometers for the detection of falsified pharmaceutical products. Talanta. 2019 Sep 1;202:469–78.

23. Tabernero P, Swamidoss I, Mayxay M, Khanthavong M, Phonlavong C, Vilayhong C, et al. A random survey of the prevalence of falsified and substandard antibiotics in the Lao PDR. J Antimicrob Chemother [Internet]. 2019 May 2 [cited 2019 Jul 11]; Available from: http://www.ncbi.nlm.nih.gov/pubmed/31049576

24. Antignac M, Diop BI, Do B, N'Guetta R, Toure IA, Zabsonre P, et al. Quality Assessment of 7 Cardiovascular Drugs in 10 Sub-Saharan Countries: The SEVEN Study. JAMA Cardiol. 2017 Feb 1;2(2):223–5.

25. Lalani M. Surveillance approaches to detect the quality of medicines in low-middle

income countries with a focus on artemisinin combination therapies for malaria. London School of Hygiene & Tropical Medicine; 2018.

26. Banerjee S, Sweet J, Sweet C, Lieberman M. Visual Recognition of Paper Analytical Device Images for Detection of Falsified Pharmaceuticals. Proc IEEE Winter Conf Appl Comput Vis [Internet]. 2017;arXiv:1704. Available from: http://arxiv.org/abs/1704.04251

27. Eberle MS, Ashenef A, Gerba H, Loehrer PJ, Lieberman M. Substandard Cisplatin Found While Screening the Quality of Anticancer Drugs From Addis Ababa, Ethiopia. JCO Glob Oncol [Internet]. 2020 Mar 6 [cited 2020 Jun 22];6(6):407–13. Available from: http://ascopubs.org/doi/10.1200/JGO.19.00365

28. Bliese SL, Maina M, Were P, Lieberman M. Detection of degraded, adulterated, and falsified ceftriaxone using paper analytical devices. Anal Methods [Internet]. 2019 Sep 26 [cited 2020 Jun 22];11(37):4727–32. Available from: http://xlink.rsc.org/?DOI=C9AY01489F

29. Weaver AA, Lieberman M. Paper test cards for presumptive testing of very low quality antimalarial medications. Am J Trop Med Hyg. 2015;92(6 Suppl):17–23.

30. Boehle KE, Carrell CS, Caraway J, Henry CS. Paper-Based Enzyme Competition Assay for Detecting Falsified β-Lactam Antibiotics. ACS Sensors. 2018 Jul 27;3(7):1299–307.

31. Nkansah P, Smine K, Pribluda V, Phanouvong S, Dunn C, Walfish S, et al. Guidance for Implementing Risk-Based Post-Marketing Quality Surveillance in Low- and Middle-Income countries. 2018.

32. Coïc L, Sacre P-Y, Avohou H, Deidda R, De Bleye C, Dumont E, et al. Recent developments of Raman spectroscopy for the qualitative analysis of falsified and substandard medicines. Eur Pharm Rev. 2018;23(6):46–8.

33. Mansouri MA, Sacré P-Y, Coïc L, De Bleye C, Dumont E, Bouklouze A, et al. Quantitation of active pharmaceutical ingredient through the packaging using Raman handheld spectrophotometers: A comparison study. Talanta. 2020 Jan 15;207:120306.

34. World Health Organization. Survey of the quality of Selected antimalarial medicineS circulating in Six countrieS of Sub-Saharan africa [Internet]. 2011 [cited 2019 Jun 4]. Available from: http://apps.who.int/medicinedocs/documents/s17835en/s17835en.pdf

35. Risha P, Msuya Z, Ndomondo-Sigonda M, Layloff T. Proficiency testing as a tool to assess the performance of visual TLC quantitation estimates. J AOAC Int. 2006;89(5):1300–4.

36. Lanzarotta A, Lorenz L, Batson JS, Flurer C, A. L, L. L, et al. Development and implementation of a pass/fail field-friendly method for detecting sildenafil in suspect pharmaceutical tablets using a handheld Raman spectrometer and silver colloids. J Pharm Biomed Anal. 2017;146:420–5.

37. US Pharmacopeial Convention. USP Technology Review: CBEx [Internet]. 2017 [cited 2020 Dec 10]. Available from: https://www.usp.org/sites/default/files/usp/document/our-work/global-public-health/tr-report-cbex.pdf

38. US Pharmacopeial Convention. USP Technology Review: Speedy Breedy [Internet]. 2017 [cited 2020 Dec 10]. Available from: https://www.usp.org/global-public-health/other-initiatives/speedy-breedy

39. US Pharmacopeial Convention. USP Technology Review: ASD QualitySpec® (Trek) [Internet]. 2019 [cited 2020 Dec 10]. Available from: https://www.who.int/medicines/regulation/ssffc/2019-6-7Final-TR-report-QualitySpek.pdf?ua=1

40. Luangasanatip N, Khonputsa P, Caillet C, Vickers S, Zambrzycki S, Fernández FM, et al. Implementation of Field Detection Devices in Medicine Quality Screening in Lao PDR – A Cost-Effectiveness Analysis. PLoS Negl Trop Dis. ,submitted.

Supplementary information captions

S1 Text. UPLC confirmatory methods protocols

S2 Table. Main characteristics and UPLC results of the medicines utilized in the evaluation

S3 Text. Instructing the trainers

S4 Table. User satisfaction questionnaire

S5 Table. Outline of the focus group discussions (5 inspectors per group discussions*)

S6 Table. Extract of the inspection record sheet for a) Evaluation Pharmacy inspections b) Sample set of medicines inspections. Note that the record sheets were adapted for the PAD, Progeny, and 4500a FTIR which are interpreted differently than other devices (e.g. inspectors were asked to fill in which column of the PAD they read for interpreting the results).

S7 Table. Time and motion study observer recording sheet in a) Evaluation Pharmacy inspection b) Sample Set of medicines inspection. Note that record sheets were adapted for the Paper Analytical Cards

S8 Table. Definition of the times measured in the evaluation pharmacy and sample set inspections

S9 Text. Results of the evaluation by device

S10 Table. Median (IQR) sampling, device testing and recording times (seconds) per sample per device in sample set testing. P-values for comparison between devices of ln (sampling time), ln (device testing time) and ln (recording time) using mixed effects generalised linear regression models adjusted on device and training, and clustered by inspectors

S11 Table. Time spent inspecting evaluation pharmacy by phase

Fig 1. Outline of the field evaluation study

Click here to access/download;Figure;Fig1.tif

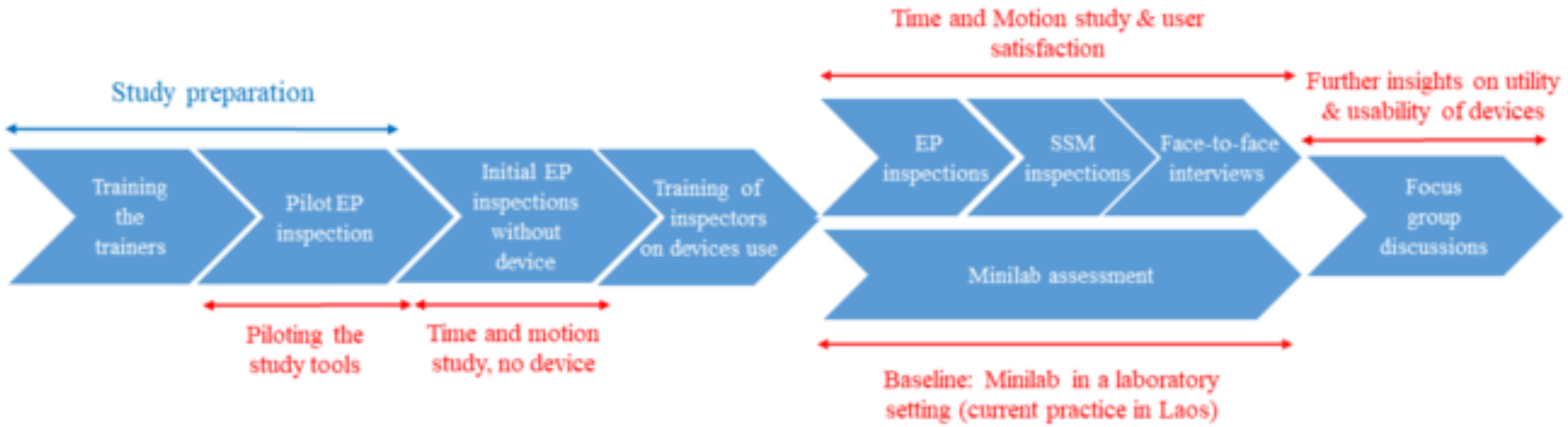

Fig 2. Time spent inspecting evaluation pharmacy, by device

Click here to access/download;Figure;Fig2.tif

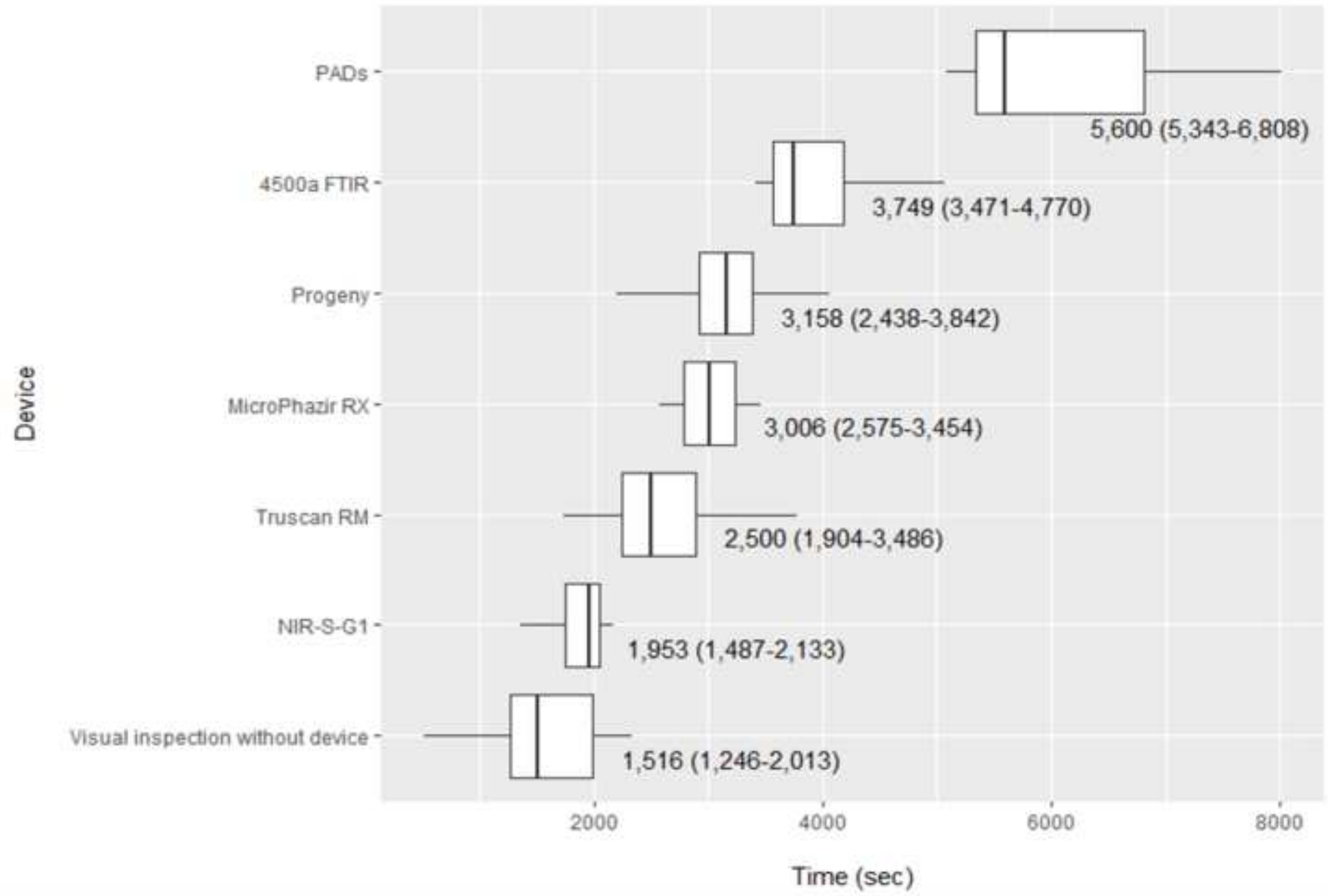

S1 Text. UPLC confirmatory methods protocols

Click here to access/download
Supporting Information
S1 Text.pdf

S2 Table. Main characteristics and UPLC results of the medicines utilized in the evaluation

Click here to access/download
Supporting Information
S2 Table.pdf

S3 Text. Instructing the trainers

Click here to access/download
Supporting Information
S3 Text.pdf

S4 Table. User satisfaction questionnaire

Click here to access/download
Supporting Information
S4 Table.pdf

S5 Table. Outline of the focus group discussions (5 inspectors per group discussions*)

Click here to access/download
Supporting Information
S5 Table.pdf

S6 Table. Extract of the inspection record sheet for a) Evaluation Pharmacy inspections b) Sample set of medicines inspections

Click here to access/download
Supporting Information
S6 Table.pdf

S7 Table. Time and motion study observer recording sheet in a) Evaluation Pharmacy inspection b) Sample Set of medicines

Click here to access/download
Supporting Information
S7 Table.pdf

S8 Table. Definition of the times measured in the evaluation pharmacy and sample set inspections

Click here to access/download

Supporting Information

S8 Table.pdf

S9 Text. Results of the evaluation by device

Click here to access/download
Supporting Information
S9 Text.pdf

S10 Table. Median (IQR) sampling, device testing and recording times (seconds) per sample per device in sample set testing

Click here to access/download
Supporting Information
S10 Table.pdf

S11 Table. Time spent inspecting evaluation pharmacy by phase

Click here to access/download
Supporting Information
S11 Table.pdf

Related Manuscript

Article 2 in Collection ‘A multi-phase evaluation of portable screening devices to assess medicines quality for national Medicines Regulatory Authorities’

Title: Laboratory evaluation of twelve portable devices for medicine quality screening.

Stephen C. Zambrzycki[1,†] Celine Caillet[2,3,4,†], Serena Vickers[2,3,4], Marcos Bouza[1], David V. Donndelinger[1], Laura C. Winalski[1], Matthew C. Bernier[1], William R. Grigger[1], Paul N Newton[2,3,4], Facundo M. Fernández[1,*].

[1] School of Chemistry and Biochemistry, Georgia Institute of Technology, Atlanta, Georgia, USA.

[2] Lao-Oxford Mahosot Hospital Wellcome Trust Research Unit, Microbiology laboratory, Mahosot Hospital, Vientiane, Lao PDR.

[3]Centre for Tropical Medicine and Global Health, Nuffield Department of Medicine, University of Oxford, United Kingdom.

[4]Infectious Diseases Data Observatory (IDDO) & WorldWide Antimalarial Resistance Network (WWARN), University of Oxford, Oxford, United Kingdom.

*Corresponding author.

E-mail: facundo.fernandez@chemistry.gatech.edu

Ph: 404 385 4432

Fax: 404 385 6447

Short Title: Devices for Medicine Quality Screening

This is the second paper in the Collection ‘A multi-phase evaluation of portable screening devices to assess medicines quality for national Medicines Regulatory Authorities’

[†]Joint first authorship

Abstract

Background:

Post-market surveillance is a key to prevent substandard and falsified (SF) medicines being consumed by patients. Field deployable technologies offer the potential for rapid objective screening for SF medicines.

Methods and Findings:

We evaluated twelve devices: three near infrared spectrometers (MicroPHAZIR RX, NIR-S-G1, Neospectra 2.5), two Raman spectrometers (Progeny, TruScan RM), one mid-infrared spectrometer (4500a), one disposable colorimetric assay (Paper Analytical Devices, PAD), one disposable immunoassay (Rapid Diagnostic Test, RDT), one portable liquid chromatograph (C-Vue), one microfluidic system (PharmaChk), one mass spectrometer (QDa), and one thin layer chromatography kit (GPHF-Minilab). Each device was tested with a series of field collected medicines (FCM) along with simulated medicines (SIM) formulated in a laboratory. The FCM and SIM ranged from samples with good quality active pharmaceutical ingredient (API) concentrations, reduced concentrations of API (80% and 50% of the API), no API, and the wrong API. All the devices had high sensitivities (91.5 to 100.0%) detecting medicines with no API or the wrong API. However, the sensitivities of each device towards samples with 50% and 80% API varied greatly, from 0% to 100%. The infrared and Raman spectrometers had variable sensitivities for detecting samples with 50% and 80% API (from 5.6% to 50.0%). The devices with the ability to quantitate API (C-Vue, PharmaChk, QDa) had sensitivities ranging from 91.7 % to 100 % to detect all poor quality samples. The specificity was lower for the quantitative C-Vue, PharmaChk, & QDa (50.0% to 91.7%) than for all the other devices in this study (95.5% to 100%).

Conclusions:

The twelve devices evaluated could detect medicines with the wrong or none of the APIs, consistent with falsified medicines, with high accuracy. However, API quantitation to detect formulations similar to those commonly found in substandards proved more difficult, requiring further technological innovation.

Author Summary

Criminally falsified or poorly manufactured medicines can lead to patients becoming sicker and losing trust in the health system. Portable tools beyond just documentation checks and visual inspection (the current practices in many poor income countries) can help pharmacy inspectors with early detection of poor quality medicines. Currently, many tools are available to detect poor quality medicines, but their performances have not been properly assessed and compared. In this study, 12 different devices ranging from disposable single use tests to portable spectrometers were tested in a laboratory. All the tested devices could identify medicines that contained none or the wrong active ingredient, a common trait of falsified medicines. Disposable tests required few resources to be implemented, but had difficulties identifying medicines with reduced amounts of active ingredients. Spectrometers used 'out-of-the-box' required minimal consumables had and had varying degrees of success at detecting medicines with reduced amounts of active ingredients. Finally, instruments with more quantitative abilities, such as benchtop simple chromatographs or mass spectrometers, offered the best sensitivity for detecting medicines with reduced amounts of active ingredients, but required the most resources and training and were deemed to be more suitable for centralized testing.

Introduction

Poor quality medicines are classified by the World Health Organization (WHO) as substandard or falsified (SF)[1]. Falsified medicines purport to be real, authorized medicines, but

they ‘deliberately/fraudulently misrepresent their identity, composition or source'[1]. Falsified medicines are the result of criminal activity and usually have packaging that is a copy of a genuine product. They may contain the incorrect amount of the correct active pharmaceutical ingredient (API), wrong API(s), or more commonly, no API at all. Substandard medicines are those authorized medical products ‘that fail to meet either their quality standards or their specifications, or both’ [1]. They negate the benefits of modern medicines, failed treatments, increased antimicrobial resistance, and distrust in the health system [2].

The recent expansion of the diversity of portable devices for medicine quality screening holds great hope for empowering inspectors in the field, making their work more cost-effective and actionable, improving MRA capacity, and protecting patients from the harm of SF medicines. However, significant knowledge gaps exist regarding the optimal choice of device, or combination of devices. These gaps impede decisions on how to best use these portable devices [3,4].

As described in the first paper in this Collection ‘A multi-phase evaluation of portable screening devices to assess medicines quality for national Medicines Regulatory Authorities’, we undertook a multi-phase collaborative project to evaluate diverse devices for SF medicine detection, producing key information required for deciding which of these technologies may be the most appropriate for field medicine quality screening.

In this second paper we present the results for the evaluation of 12 portable screening technologies for distinguishing between genuine good quality, 50% and 80% API medicines (mimicking substandard medicines), and 0% and wrong API medicines (mimicking falsified products) under controlled laboratory conditions. We present protocols for utilizing each device, an evaluation of the devices’ ease of operation and diagnostic accuracy, and an assessment of the

devices' resource needs. We close with suggestions of which devices were prioritized for field testing, described in the third paper of the series.

Methods and Materials

Devices:

Following a detailed literature review [5] and further discussions with experts, twelve devices were chosen for evaluation (Table 1). These included single-use Paper Analytical Devices (PAD), Rapid Diagnostic Tests (RDT), a portable liquid chromatograph, a single quadrupole benchtop mass spectrometer, the PharmaChk microfluidic device, an attenuated total reflectance mid-IR (MIR) spectrometer, three different near-infrared (NIR) spectrometers, two Raman spectrometers, and the Minilab thin layer chromatography kit. Brief fundamentals of each underlying technology in explained S1 Appendix.

These 12 devices were chosen to represent a variety of different technologies within the project's budget and time constraints. The ideal device should be portable, battery powered, durable, and require minimal training, consumables, sample preparation, and test the APIs in this study. The two Raman spectrometers (TruScan RM and Progeny) were chosen for their different laser excitation sources. The infrared spectrometers (4500a, MicroPHAZIR RX, Neospectra 2.5, NIR-S-G1) were selected because they each had different testing modalities along with sampling in different infrared spectral ranges. The remaining devices were selected because they are representative of a key technology used in the screening of small molecule medicines. Key configuration exceptions and constraints are described in S2 Appendix.

Table 1: List of instruments and devices evaluated, including their underlying technology and basic instrument specifications. Photographs of each device are in S3 Appendix. Detailed descriptions of each of these devices, including a more detailed overview of the technology and device specifications, are provided in S4 Appendix.

**Technology**	**Device Name (Manufacturer)**	**Basic Instrument Specifications**	**APIs Tested**	**Market Status at the Time of this Study**
Colorimetric Assay	Paper Analytical Devices (PAD, Notre Dame University).	12 colorimetric chemical tests on a single-use paper card.	A, AZITH, P, OFLO, & SM	Under Development [6–8]
Lateral Flow Immunoassay	Rapid Diagnostic Tests, (RDT, China Agricultural University of Beijing and University of Pennsylvania).	Single-use disposable dipsticks, concentration specific.	Artemether, ART, & DHA	Under Development [9–11]
Liquid Chromatography	C-Vue (C-Vue).	Mercury lamp (≈254 nm) detector; Millipore Chromolith RP18e 25 x 4.6 mm column.	ACA, OFLO, & SMTM	Marketed [12]
Mass Spectrometry	QDa (Waters).	Selected ion monitoring mode per API; flow injection analysis.	All	Marketed [13]
Microfluidics	PharmaChk (Boston University).	Luminescence detection, 490 & 515 nm LED & filters.	ART	Under Development [14]
Mid-Infrared Spectroscopy	4500a FTIR Single Reflection† (Agilent)	Attenuated Total Reflectance. Spectral range: 2,500-15,384 nm.	All	Marketed [15]
Near-Infrared Spectroscopy	NeoSpectra 2.5* (Si-Ware).	Spectral range: 1,350-2,500 nm.	All	Marketed [16]
	MicroPHAZIR RX*† (Thermo Fisher Scientific).	Spectral range: 1,600-2,400 nm.	All	Marketed [17]
	NIR-S-G1*†° (Innospectra).	Spectral range: 900-1,700 nm.	All	Marketed [18,19]
Raman Spectroscopy	Progeny*† (Rigaku).	Excitation Laser: 1,064 nm, Spectral range 200-2,500 cm^{-1}.	All	Marketed [20]
	TruScan RM*† (Thermo Fisher Scientific).	Excitation Laser: 785 nm, Spectral range 250-2,875 cm^{-1}.	All	Marketed [21]
Thin Layer Chromatography	Minilab‡ (Global Pharma Health Fund E.V.).	Detection by chemical staining and UV light exposure.	All	Marketed [22]

A: Amoxicillin; API, Active pharmaceutical ingredient; ART: Artesunate; AZITH: Azithromycin; CA: Clavulanic Acid; DHA: Dihydroartemisinin; FTIR: Fourier Transform Infrared, OLFO: Ofloxacin; P: Piperaquine; SM: Sulfamethoxazole; TM: Trimethoprim.
*Instrument/device could also scan samples through transparent packaging.
†Automatic spectral reference library comparison capabilities.
‡Only TLC portion of the Minilab was used in this study, but not the dissolution and weighing tests.
°Also referred to in this study as, “NIRscan”, the beta version used in this study.

The device specific operating protocols developed for this project, sample preparation conditions, and instrument parameters are detailed in S5 Appendix.

Samples:

Antibiotic and antimalarial formulations represent a majority of the total number of SF medicines reported to the WHO Global Surveillance and Monitoring System between 2013-2017 [23]. Seven key antibiotics and antimalarials used in South-East Asia were chosen for device evaluation: AL = Artemether-Lumefantrine; ART=Artesunate; AZITH=Azithromycin; ACA= Amoxicillin-Clavulanic Acid; DHAP=Dihydroartemisinin-Piperaquine; OLFO=Ofloxacin; and SMTM=Sulfamethoxazole-Trimethoprim.

Field-collected medicines (FCM) and simulated medicines (SIM) were used to test the devices. FCM included good quality, falsified, and “look-alike” medicines. All FCM samples were tested with ultra-performance liquid chromatography (UPLC) or mass spectrometry (MS) to ensure quality (S6 Appendix). Good quality FCM were purchased from reliable wholesalers in the Greater Mekong Sub-Region or donated by manufacturers. Falsified FCM were collected during earlier studies (see [24] and references therein). Look-alike FCM were visually indistinguishable from genuine medicines, and contained APIs not evaluated in this study [25]. Five brands of FCM were sold in transparent packaging such as clear plastic blister packs or clear glass vials. Five devices tested claimed to be capable of performing nondestructive testing through transparent barriers (Table 1), whenever feasible, FCM were also evaluated while sealed in their original packaging. Field-collected parenteral artesunate (Artesun®) powder was removed from the vial and transferred to a plastic re-sealable bag for testing with Raman instruments because the powder was too dispersed in the original vial for one of the spectrometers to yield a stable signal and a consistent result. Device operators were not blinded to the identity of the samples being tested.

Most portable devices are only available for checking the presence/absence of the API or whole formulation spectra. Therefore, we did not examine dissolution rates, homogeneity of the API distribution within tablets, or tablet coating thicknesses. SIM were produced in the laboratory to mimic good quality, 80% and 50% API concentration tablets, mimicking substandard medicines, and no API and wrong API tablets, mimicking falsified medicines. All SIM were prepared as 100 mg 6 mm-diameter tablets, except for ART which was tested as a loose powder to simulate Artesun®. ART, AZITH, OLFO, and SMTM powders (>98% purity) were purchased from TCI Chemical (Portland, OR). For simulated ACA, AL, and DHAP medicines, the APIs were sourced from good quality FCM (AMK® 1000 mg for ACA, Coartem® for AL, and D-Artepp® for DHAP). These FCM tablets were crushed, mixed, and re-pressed into tablets. The excipients included cellulose, lactose, or starch as the bulking agents, and magnesium stearate as the lubricant, all sourced from Sigma Aldrich (St. Louis, MO). Magnesium stearate was excluded from the ART SIM formulation. To mimic 80% and 50% substandards, the excipients listed above were added to dilute the API. Acetaminophen was used as the wrong API for falsified SIM and was sourced from Sigma Aldrich (St. Louis, MO). Details on specific formulations and methods for making each SIM are found in S7 Appendix, and protocols describing sample handling for each instrument/device in S8 Appendix.

Data Analysis:

When testing medicine samples, devices generated results that were either qualitative or quantitative. Qualitative results were based upon pattern comparison between a known good quality medicine reference and the test medicine sample data. PAD chemically reacted with the medicine ingredients generating a color pattern, that was then visually compared to a reference photograph to determine the presence of an API. The 4500a, MicroPHAZIR RX, NIR-S-G1, Progeny, and TruScan RM spectrometers computationally compared experimentally-collected

spectra to reference spectra of good quality medicines stored in the device's database. Each sample spectrum acquired was given a score by the device software resulting from the comparison with the good quality reference medicine spectrum. Such scores had to meet a given threshold to determine if a medicine passed (Fig 2A). Reference library creation is described in S9 Appendix. For the NIR-S-G1 spectrometer, reference samples were sent to the developer who prepared the reference libraries. The passing threshold values for the correlation coefficient or p-value testing initially set as default by the developer in the MicroPHAZIR RX, NIR-S-G1, Progeny, and TruScan RM spectrometers were utilized. These devices would directly tell the user 'pass' or 'fail', which were recorded. The pass threshold for the 4500a MIR spectrometer's correlation coefficient was set by us at >0.9 because the device would not output a direct pass/fail result, but rather give a list of matches with their associated correlation coefficients. The Neospectra 2.5 spectrometer did not include software to compare the experimentally-collected spectra to reference spectra. Therefore, experimental Neospectra 2.5 spectra were overlaid with reference spectra and visually compared by an analyst blinded to sample identity for determining the final pass/fail result.

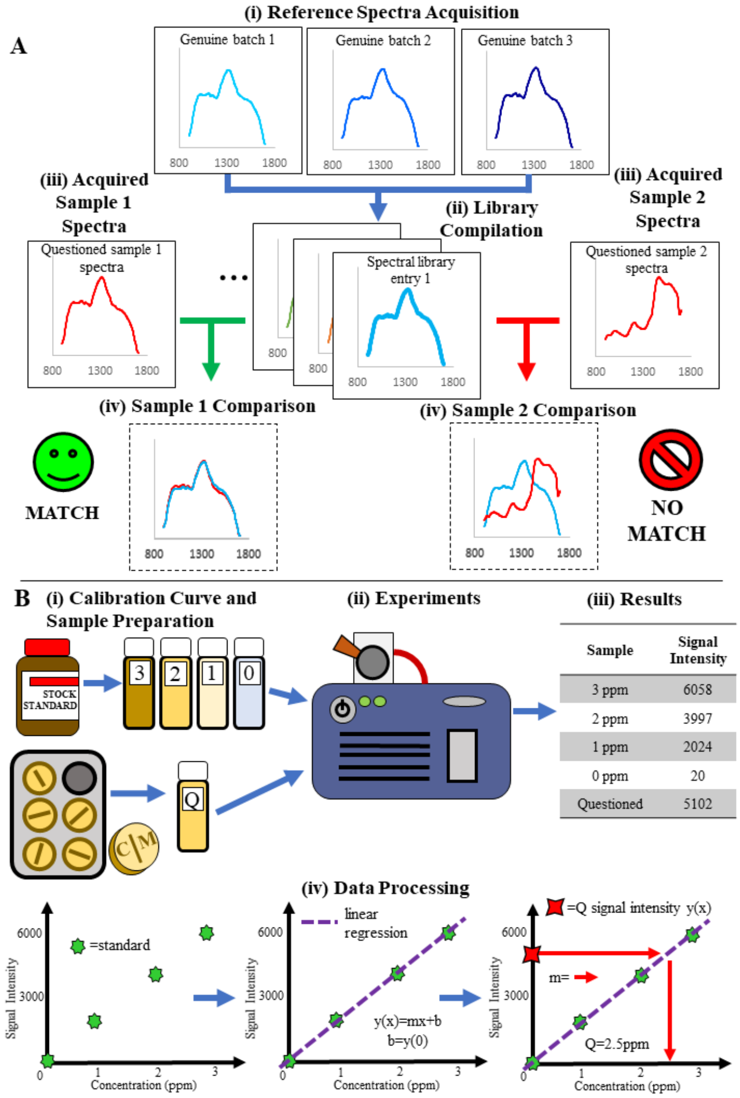

Figure 2. Illustration of the basic processes for qualitative spectral comparison (A) and quantitative analysis (B). **(A)** Process for reference library creation and spectral comparison analysis. From top to bottom: (i) spectra are collected from different batches of the same medicine and compiled into a mean spectrum representative of that medicine. (ii) This mean spectrum is used to build a "library" or database that serves as the comparator against which test samples are compared. (iii) Test samples are scanned and then (iv) the test sample spectra are overlaid with the reference spectrum for visual or computational comparison to determine a pass or fail. **(B)** Illustration of a basic quantitative experiment. From left to right and top to bottom. (i) A set of standard calibration samples with increasing API concentration is prepared along with a solution of the test sample that should fit in the concentration range of those standards. (ii) All solutions are then tested on the instrument and (iii) the data collected. (iv) The data obtained is then used to build a calibration curve via linear least-squares regression. Interpolation of the peak area of the questioned sample into this curve yields the estimated API concentration.

To estimate the amount of API with quantitative instruments (C-Vue, PharmaChk and QDa) a calibration curve approach was used (Fig 2B). After sample preparation and data acquisition with the instruments, the calculated API amount had to be within ± 10 % of the stated API amount for the sample to be classified as good quality, for both single and combination API medicines. The reference ranges of percent API(s) vary according to different pharmacopeias and different APIs (see S10 Appendix) so for simplicity, we considered that medicines with %API outside the 90-110% range of the manufacturer's stated amount of API(s) were out of specification for all APIs included.

Both the RDT and the Minilab-TLC are semi-quantitative devices. RDT rely on the color density of control and test lines to confirm the presence of an API at a specific concentration. The TLC portion of the Minilab kit relies on the size and migration distance of a spot that is formed by a small sample deposited on the TLC plate, compared to a good quality reference standard on the same plate.

One of the major differences between devices was whether they were specific for the API, or if they could screen the entire formulation (see S11 Appendix).

When a sample failed the first test, we operated a 'best of three' system for overall sample classification (out of the three tests performed on the failing samples, the most frequently occurring of 'pass' or 'fail' would then be the overall sample classification), in the absence of devices manufacturer's guidelines. For the single-use RDT the failing samples were rerun only once due to a limited number of tests available. For the PAD, the failing samples were rerun once, as recommended by the developer. For both the RDT and PAD a third run was conducted when the first two test results were discordant, and the 'best of three' result was retained.

Statistical Analysis

The overall sample binary classification (‘pass’ or ‘fail’) was used to calculate the sensitivity and specificity for each instrument. Sensitivity was defined as the percentage of true positives over the total of true positives and false negatives, and specificity as the percentage of true negatives over the total of true negatives and false positives. A true positive was defined as the sample being poor quality with the device correctly giving a fail result. Sensitivity and specificity were expressed as percentages and their 95% confidence intervals (95% CI). The exact confidence interval was based on Jeffreys’ confidence interval formula [26]. Sensitivities and specificities were compared by device pairs using McNemar tests. Data analysis was carried out using Microsoft Excel 2013 and STATA 14.2. The level of significance was set at p=0.05 (two-sided).

To gauge whether the passing correlation coefficient or p-value threshold values initially set in the 4500a, MicroPHAZIR RX, Progeny, and TruScan RM spectrometers were optimal, receiver operating characteristic (ROC) curves were created. Only substandard and good quality SIM were used in the ROC curve analysis because these samples were shown to be the most challenging for the devices to distinguish. A variety of thresholds were tested to best visualize the curve and optimize the sensitivity and false positive rate (1-specificity). ROC curve analysis was also applied to the C-Vue, PharmaChk, and QDA, but the correlation coefficient or p-value thresholds were replaced with percent concentration thresholds.

Results

All devices showed 100% sensitivity to correctly identify tablets with 0% or wrong API after removal from their packaging, except for the NIR-S-G1 that showed a sensitivity (95% CI) of 91.5% (79.6-97.6%,). Specificities of 100% were observed for most of the devices, except the

C-Vue [60.0% (32.3-83.7%)], PharmaChk [50.0% (1.3-98.7%)], Progeny [95.5% (77.2-99.9%)] and the QDa [91.7% (73.0-99.0%)](Table 2). The following subsections detail significant strengths and weaknesses observed for each during testing.

Table 2. Sensitivity and specificity to correctly determine quality of medicine samples for the 12 tested devices.

**Devices**	**0% API and wrong API samples**		**Genuine Good Quality**		**50% and 80% API samples**		**All poor quality samples**	
--------	**Sensitivity (95% CI)**	**n**	**Specificity (95% CI)**	**n**	**Sensitivity (95% CI)**	**n**	**Sensitivity (95% CI)**	**n**
*4500a FTIR**	100 (93.3-100)	53	100 (85.8-100)	24	28.6 (15.7-44.6)	42	68.4 (58.1-77.6)	95
C-Vue	100 (82.4-100)	19	60.0 (32.3-83.7)	15	100 (81.5-100)	18	100 (90.5-100)	37
*MicroPHAZIR RX**	100 (92.5-100)	47	100 (84.6-100)	22	50.0 (32.9-67.1)	36	78.3 (67.9-86.6)	83
Minilab	100 (93.3-100)	53	100 (85.8-100)	24	59.5 (43.3-74.4)	42	82.1 (72.9-89.2)	95
*Neospectra 2.5**	100 (92.5-100)	47	100 (84.6-100)	22	5.6 (0.7-18.7)	36	59.0 (47.7-69.7)	83
NIR-S-G1*	91.5 (79.6-97.6)	47	100 (84.6-100)	22	30.6 (16.3-48.1)	36	65.1 (53.8-75.2)	83
PAD	100 (88.8-100)	31	100 (83.2-100)	20	0 (0-11.6)	30	50.8 (37.7-63.9)	61
PharmaChk	100 (54.1-100)	6	50.0 (1.3-98.7)	2	83.3 (35.9-99.6)	6	91.7 (61.5-99.8)	12
*Progeny**	100 (92.5-100)	47	95.5 (77.2-99.9)	22	16.7 (6.4-32.8)	36	63.9 (52.6-74.1)	83
QDa	100 (93.3-100)	53	91.7 (73.0-99.0)	24	100 (91.6-100)	42	100 (96.2-100)	95
RDT	100 (73.5-100)	12	100 (29.2-100)	3	16.7 (2.1-48.4)	12	58.3 (36.6-77.9)	24
*TruScan RM**	100 (92.5-100)	47	100 (84.6-100)	22	22.2 (10.1-39.2)	36	66.3 (55.1-76.3)	83

*With substantial and upfront work, optical spectrometers could theoretically perform API quantitation. Parameters could be adjusted for better analysis of medicines containing lower-than-stated amount of API(s). However, in this study, only default parameters provided by the manufacturer were used. It is believed, however, that potential enhancements in sensitivity and specificity could be made by optimizing threshold values and experimental settings for specific medicines.

Thin Layer Chromatography: Minilab

The Minilab TLC kit consisted of a hard-shell case containing all the chemical analysis equipment necessary to perform TLC experiments. It correctly characterized all good quality, no-API falsified, and wrong-API falsified FCM and SIM. Only 1 out of 21 of the SIM 50% API substandards were misidentified as good quality. A majority (16/21) of SIM 80% API substandards were misidentified as good quality as differences between the 100% and 80% reference spots on the TLC plates were difficult to distinguish visually. The Minilab is designed to detect samples with API below 80% API. The primary limitation identified was the higher requirements in terms of resources needed to conduct experiments, and the difficulties associated with visual interpretation of the results. Its primary strength was the wide variety of APIs covered and its detailed instructions (see S12 Appendix).

Single Use Devices: PAD and RDT

The PAD are paper card colorimetric assays with 12 testing lanes. Test samples were applied by rubbing them onto the testing lanes. The bottom of the cards are placed in water, where they develop to generate a color pattern that confirms the presence (or not) of a given API. They correctly characterized all good quality, no-API falsified, and wrong-API falsified FCM and SIM. The PAD were not designed to detect substandard medicines containing lower API amounts than stated and all 50% and 80% API substandards samples were incorrectly classified as good quality. The primary limitation of PAD was that the colors were difficult to interpret for weakly colored reaction products (S13 Appendix). Their primary strengths were that the experiments required minimal effort and consumables (see S14 Appendix).

RDT are lateral flow immunoassays that typically target a single API. They have the stated capability of distinguishing between substandard and falsified antimalarials. A subset of the RDT tested claimed to be able to detect artemether in artemether-lumefantrine co-formulated medicines.

These RDT, however, were defective and not considered further. RDT specific for DHA and ART had a sensitivity of 16.7% in detecting substandards. RDT correctly characterized all the remaining good quality, no-API falsified, and wrong-API FCM and SIM. The primary limitations of RDT were the additional consumables required for their operation and the relatively extensive sample preparation needed compared to PAD. In a few cases it was difficult to read the RDT control lines on the cartridges due to inconsistent colors observed between RDT units (S15 Appendix). One advantage of RDT over PAD was their ability to detect some substandards (see S16 Appendix).

MIR Spectrometer: 4500a

The 4500a is a MIR spectrometer that performs attenuated total reflection experiments on powdered samples. It is controlled through a computer or Windows-based smartphone. With a correlation coefficient threshold of >0.9 for pass/fail analysis, the 4500a MIR spectrometer correctly characterized all good quality, no-API falsified, and wrong-API falsified FCM and SIM. None of the 80% API substandard SIM were correctly classified as poor quality; however, over half (12/21) of the 50% API substandard SIM were correctly classified as poor quality. Primary weaknesses were its inability to test through transparent packaging because many plastics completely absorb MIR radiation, and the need to crush samples into powders. A major strength of the 4500a MIR spectrometer software was the step-by-step instructions provided to aid in correcting background, cleaning the unit, and labeling IR spectra (see S17 Appendix).

NIR Spectrometers: Neospectra 2.5, NIR-S-G1, & MicroPHAZIR RX

The Neospectra 2.5 Fourier transform NIR detector module can be used to build a device that fits a user’s custom needs. It correctly characterized all good quality, no-API falsified, and wrong-API falsified FCM and SIM. All the 80% API substandards and 16 out of 21 of the 50% API substandards were misidentified as good quality samples. The primary limitation was the inability to conduct automated spectral library matching. Although users can extract the raw

spectra using third-party library comparison tools, this feature was not evaluated as it was outside the study scope. Its modularity was its primary strength, allowing to configure the system for sampling both solids and liquids. Its detector had the widest NIR wavelength range in this study, allowing for more spectral information to be collected (see S18 Appendix).

The NIR-S-G1 NIR spectrometer is controlled by a smartphone via Bluetooth®. Although this instrument correctly characterized all good quality medicines, it had difficulties in the analysis of the OFLO SIM. The no-API falsified, 50% substandard, and 80% substandard OFLO SIM were all incorrectly deemed as being good quality. This may have been because the limited device spectral range (Fig 3) did not reveal a sufficiently large number of significant features when comparing against the reference library entry. The library processing software was not able to detect the relatively small spectral differences observed in the ~1500 nm region (Fig 3B). Since this device was able to correctly determine that the wrong-API OFLO medicines were poor quality, the API itself may have had limited NIR spectral features distinguishable from the excipients. Additionally, one falsified SIM sample containing only starch was misidentified as good quality DHAP. The simulated DHAP sample used D-Artepp® as the source of API. It is possible that if D-Artepp® contained starch, this excipient may have contributed to the misclassification. Cellulose and lactose-containing falsified tablets were correctly characterized as poor quality DHAP. All other falsified samples were correctly identified. Four out of 21 (19%) of the 80% API substandards, and 10 out of 21 (48%) of the 50% API substandards were correctly identified as poor quality. The limited spectral range and limited chain-of-custody capabilities, which included the inability to record tested sample information in the software at the time of testing, were key limitations. The primary strengths were the user interface simplicity and that the NIR-S-G1 was the most portable spectrometer tested (see S19 Appendix).

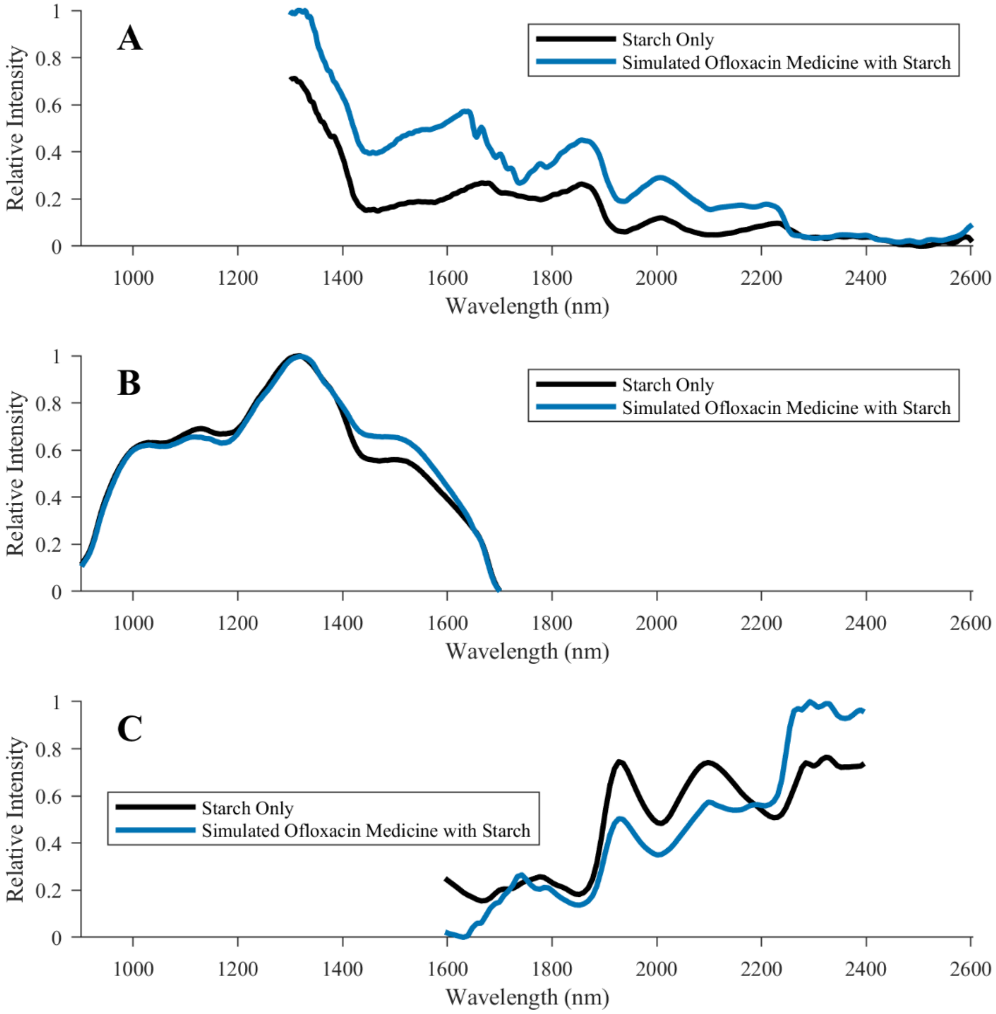

Figure 3.Comparison of NIR spectra obtained for ofloxacin-containing simulated medicines using the **(A)** Neospectra 2.5, **(B)** NIR-S-G1, and **(C)** MicroPHAZIR RX spectrometers. The black trace is of a falsified simulated medicine tablet containing only starch. The blue trace is of a simulated good quality ofloxacin sample that contained starch as the bulk excipient.

The MicroPHAZIR RX handheld NIR spectrometer contains the instrument and user interface in one module. It correctly characterized all good quality, 50% API substandard, no-API falsified, and wrong-API falsified FCM and SIM. Only 1 of the 21 (4.8%) 80% API SIM substandards was correctly classified as poor quality. The primary limitation of the MicroPHAZIR RX were the instrument's bulkiness and advanced skills necessary to process the spectra to generate reference libraries, but it had an easy-to-use interface. (see S20 Appendix).

Raman Spectrometers: Progeny and TruScan RM

The Progeny is a handheld Raman spectrometer with a 1064 nm excitation laser, resulting in lower fluorescence than shorter wavelength lasers. It correctly characterized all the no-API falsified and wrong-API falsified FCM and SIM. All good quality samples were correctly characterized, except the FCM Augmentin® (ACA), which was incorrectly determined as being Roxythroxyl® (roxithromycin). The outer tablet coating of Augmentin® may have been chemically similar to that of Roxythroxyl®, a spectra stored in the master Progeny reference library. None of the 80% SIM substandards and only 7 out of 21 (33%) of the 50% SIM substandards were correctly characterized as poor quality. The Progeny spectrometer was heavier than other units (1.6 kg) and risked burning samples with the laser if the instrument's focal length was not properly set-up. The primary strengths were its simple to use touch screen interface, and the lack of fluorescence signals that could overwhelm the detector (see S21 Appendix).

The TruScan RM is a handheld Raman spectrometer with a 756 nm excitation laser that correctly classified all the good quality, no-API falsified, and wrong-API falsified medicines in the FCM and SIM collections. The device only correctly classified 3 out of 21 (14%) SIM 80% API, all three being DHAP samples. Seven out of 21 (33%) SIM 50 % API substandard were correctly classified. Limitations included the need for significant computer knowledge (e.g. setting

up IP addresses, firewalls) to correctly complete the initial setup with the master computer and the excitation laser that caused significant fluorescence for ACA FCM and SIM samples when compared to the other non-destructive spectrometers tested (S22 Appendix). Sampling Artesun® through the original sample glass vial yielded spectra that were indistinguishable from those of an empty glass vial, whereas this issue was not observed with the Progeny (Fig 4). The primary strengths of the TruScan RM were its relatively lower weight and bulkiness when compared with the Progeny and its ease of use (see S23 Appendix).

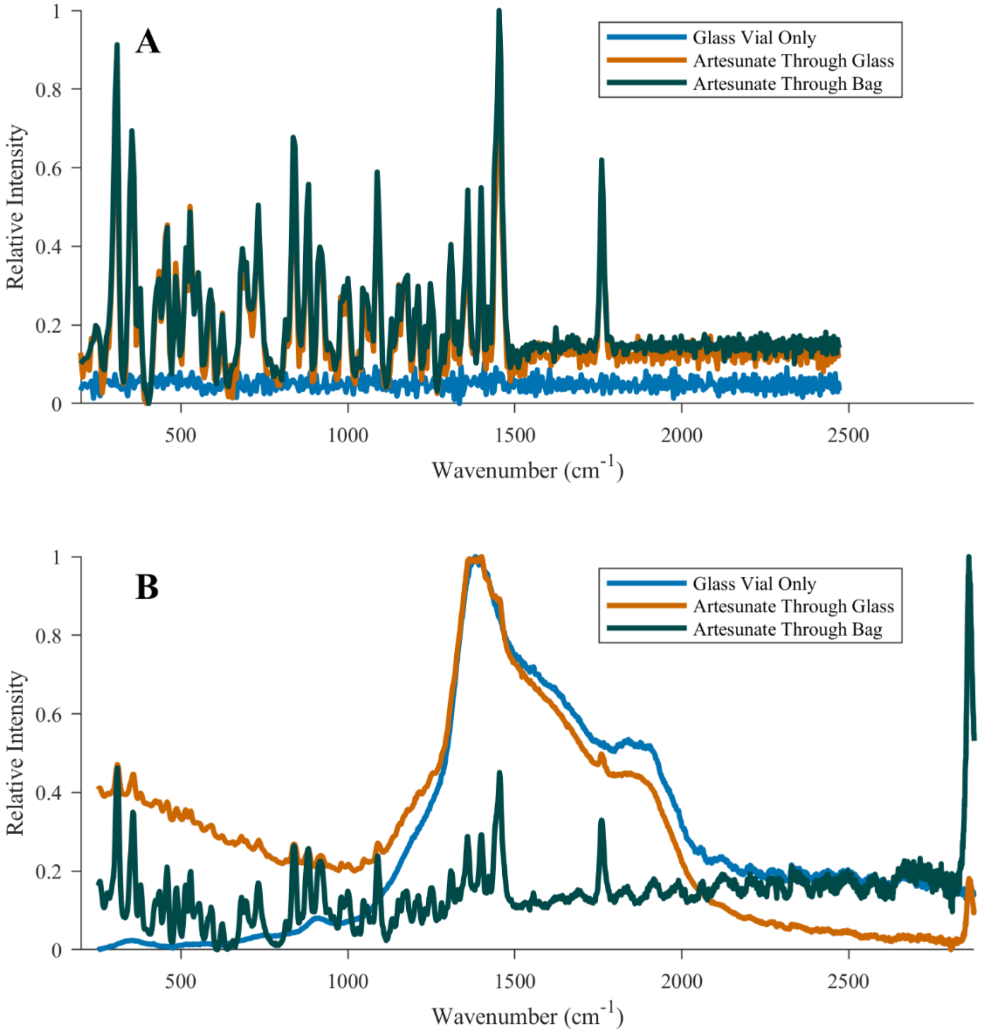

Figure 4. Comparison of Raman spectra obtained with the **(A)** Progeny and **(B)** TruScan RM spectrometers for Artesun® artesunate powder for injection. Spectra are provided for 1) a scan of the bottom of the Artesun® glass vial containing no artesunate (blue trace), 2) a sample containing 60 mg of artesunate powder, scanned through the bottom of the glass vial (orange trace), and 3) the artesunate powder transferred to a polypropylene bag and compacted into a more localized area to enable more focused analysis (green trace).

Quantitative Instruments: C-Vue, PharmaChk, & QDa

The C-Vue is a portable lightweight tabletop liquid chromatograph equipped with a manual syringe pump, a manual injector, a reverse-phase column, two detectors, and a control laptop. It was able to correctly characterize all the 80% API substandard, 50% API substandard, no-API, and wrong-API OFLO, SMTM, and ACA. The C-Vue specificity was one of the lowest in this study (60% good quality FCM and SIM misidentified as poor quality), potentially due to potential matrix effects from the excipients that were not used in the preparation of the calibration solutions. The lowest specificity was observed for medicines formulated with more than one API (0% and 40% for ACA and SMTM, respectively), but none of the single API good quality OFLO FCM and SIM were misclassified. Two notable limitations are its inability to detect artemisinin derivatives with the setup tested and the significant effort required to continuously ensure that the pump was pressurized by hand. Newer versions of this instrument include an upgraded mobile phase electric pump that simplifies operation. Its primary strength was the simplicity of its design, making it easy to transport, repair, or modify in the field (see S24 Appendix).

The PharmaChk is a portable microfluidic system that uses luminol-based chemiluminescence detection, contained inside a hard-shell case. The system can automatically calibrate itself and assay samples in a single experiment. It was able to correctly characterize all the 50% API substandard, no-API falsified, and wrong-API falsified ART SIM. One of the three 80% API substandards was misclassified as good quality. The FCM good quality ART was correctly classified, while the SIM 100% ART sample was incorrectly classified in two trials as poor quality, with yields of 51.6 mg (86%) and 53.75 mg (89.5%) of the stated 60 mg amount. Its primary limitations were that the prototype used could only test for ART and the reagents degraded within a few hours after being prepared. Its primary strengths system were the automation of all

API concentration calculations in the embedded computer system, the capability of simultaneously infusing reagent and samples, and the clear step by step instructions (see S25 Appendix).

The QDa is a benchtop single quadrupole mass spectrometer with an electrospray ion source. All the 80% API substandards, 50% API substandards, no-API falsified, and wrong-API falsified medicines were correctly classified. Two good quality FCM were misclassified as poor quality, likely due to incomplete API extraction. Limitations of the QDa were that it required significantly more extensive sample preparation than the C-Vue or the PharmaChk because it required several dilutions for the tested sample for the extracts to fall within the linear range of the instrument and not overwhelm the detector. The requirements for additional consumables such as compressed nitrogen gas, and the higher mechanical complexity of the instrument could make portability and serviceability challenging. Its primary strength was its quantitative capabilities for a wide range of APIs, and the high sample throughput once sample preparation was complete (see S26 Appendix).

Device Comparison

Paired-wise comparisons of the sensitivities showed that no device had statistically significantly lower or higher sensitivities to correctly identify 0% and wrong API samples than any other device (S27.1 Appendix**)**.

Specificity of the C-Vue was significantly lower than that of all other devices except the Progeny ($p=0.0625$) and the QDa ($p=0.1250$) (S27.2 Appendix**)**. The performances of the PharmaChk and RDT could not be compared with the C-Vue as this device was limited to test ACA, OFLO and SMTM in the present study. Because only a few genuine medicine samples were available for specificity calculations, the interpretation of statistical comparisons is limited.

The C-Vue and QDa performed better to correctly identify 50% and 80% API samples than other devices they could be compared to, except the PharmChk ($p<0.05$) (S27.3 Appendix); the Minilab showed higher sensitivity than other devices, except the C-Vue, QDa, PharmChk and MicroPHAZIR RX ($p<0.05$). The MicroPHAZIR RX showed higher sensitivity than other spectrometers ($p<0.05$) to correctly identify 50% and 80% API samples except the NIR-S-G1 ($p=0.0923$); the Neospectra 2.5 had lower sensitivity than other spectrometers ($p<0.05$) except the Progeny ($p=0.2188$). The PAD showed significantly lower sensitivity to correctly identify 50% and 80% API samples than the other devices except the RDT ($p=1.0$) and the Neospectra 2.5 ($p=0.50$) but the number of samples for comparisons was limited.

Discussion

These results suggest that all tested devices are well-suited for detecting no API and wrong API medicines, these being common falsified medicines chemical compositions. Although detecting falsified medicines is of great public health importance, the co-detection of substandard medicines is also essential, especially as these will lead to cryptic therapeutic failure and engender antimicrobial resistance. Much needed technological improvements, as revealed by this study, are the ability to quantitate APIs *via* Raman/IR spectrometers or disposable devices, and improvement of the specificity of some of the quantitative devices. Although all substandards were successfully detected with quantitative devices, except two ART samples with the PharmaChk, their less-than-optimal specificity is of concern. Reduced specificities increase the number of confirmatory testing required in quality control laboratories, resulting in increasing cost and loss of time and resources. Quarantine or withdrawal of the samples failing the screening technologies from the market due to device limitations can also negatively impact manufacturers, pharmacies, and ultimately patients.

Many FCM tablets tested had coatings that made sampling of the tablet bulk difficult by spectroscopic techniques. To illustrate this point, the spectrometers (except the 4500a FTIR that cannot scan intact tablets) were used to sample AMK® 1000 mg (ACA) tablets, both intact and crushed. The corresponding overlaid spectra shown in S22 Appendix indicate clear differences between the spectra of coated and crushed medicines. The spectral fingerprint of the outer coating of intact tablets, depending on coating composition and thickness, may be insufficient to derive the quality of the medicine itself as a whole. Reference library creation could also be affected if the operator sampled the coating but attempted to match the spectrum to that of a crushed tablet. We were unable to find evidence on the consequences of different coatings and their thickness on spectra [5]. S28 Appendix summarizes the perceived difficulty with testing different medicine formulations, such as capsules, water based medicines, powders, and creams/gels with each device.

Optimization of the spectrometers' software's pass/fail threshold values in performing database matching could boost the instruments' sensitivities and specificities. The ROC curves constructed in Fig 5 for SIM with the 4500a, MicroPHAZIR RX, Progeny, and TruScan RM spectrometers show that setting a higher threshold improve devices sensitivities, but at the cost of reduced specificity. The quantitative devices' precision could also be optimized by adjusting the concentration threshold used for pass/fail decisions. Lowering the pass threshold concentrations for the quantitative devices (C-Vue, PharmaChk, & QDa) improves their specificities, but at the cost of reduced sensitivity (S29 Appendix). Therefore, the pass/fail threshold values for these instruments must be balanced to optimize the reliability of medicine quality classification. In most circumstances public health risks suggest that optimizing sensitivity for detecting SF medicines will be more important than specificity.

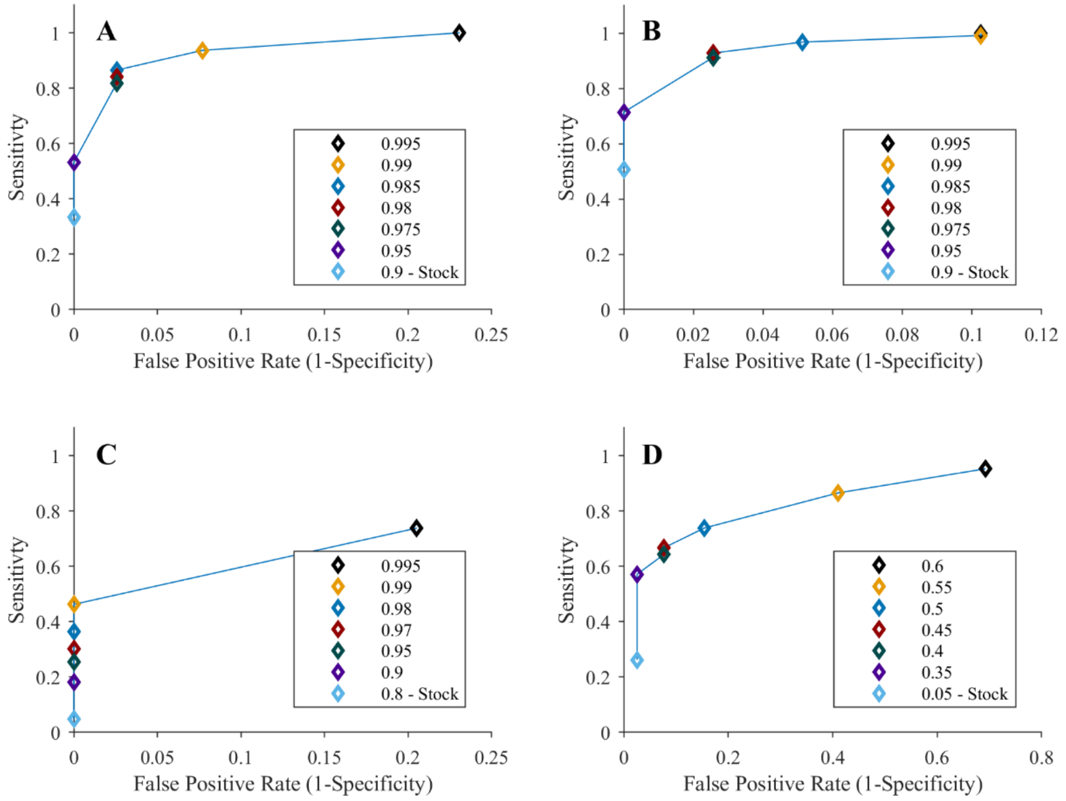

Figure 5. Receiver operating characteristic (ROC) curves for the **(A)** 4500a, **(B)** MicroPHAZIR RX, **(C)** Progeny, and **(D)** TruScan RM spectrometers. ROC curves were based only on the results for simulated substandard and good quality medicines. Each legend identifies the threshold chosen for each point, with the one labelled "Stock" being the threshold used for the study. The stock thresholds for the MicroPHAZIR RX's correlation coefficient, Progeny's correlation coefficient, and TruScan RM's p-value were the default values set by the manufacturer. The 4500a stock threshold was selected for the study since that instrument did not output pass/fail results.

Limitations of our study include: the limited number of APIs tested; difficulties with reference libraries creation for spectrometers; the limited comparability of reference libraries due to the variability of spectrometers; the investigators not being blinded to sample identity; the limited optimization of the devices to allow API quantitation; and the lack of power to compare sensitivities and specificities between devices (see S30 Appendix for more details).

With additional development and optimization, many of these devices may be able to perform both quantitative and qualitative analysis. For example, methods could be developed for spectrometers to perform quantitative analyses by selecting a spectral feature correlated to a specific chemical bond in an API, or by monitoring changes in the entire spectrum and correlate those to the differences in API concentration. A key obstacle to portable spectrometer quantitation would be instrument calibration per brand because they analyze the entire formulation that will vary between different excipient recipes. To ensure a reliable calibration at a variety of different API concentrations, the calibrants should be chemically similar to the medicine of interest due to potential matrix effects. The QDa mass spectrometer could be adapted for qualitative analysis by allowing the instrument to record the entire mass spectrum to analyze as many ingredients in the formulation as possible. However, analyzing a wider mass range risks less accurate quantitation compared to single ion monitoring used in this study.

Which device is optimal for different positions within the medicine distribution system will depend on the question being asked. For example, what type of medicine or API is tested, whether qualitative or quantitative assays are required, and what human and financial capacity is available. The Minilab, PAD, and RDT require minimal training and infrastructure to implement in the field rapidly. However, the PAD and RDT are limited due to narrow range of detected API. The spectrometers have the advantages of being non-destructive (except the 4500a MIR spectrometer)

and can analyze a broader API diversity. The spectrometers suffer from issues with tablet coatings, the spectral range of the NIR-S-G1 spectrometer and fluorescence issues with the TruScan RM can reduce accuracy for some medicines. The smartphone simplicity with the NIR-S-G1 is highly desirable. With the increasing global prevalence of smartphone technology, connectivity using cell phone networks, and Wi-Fi to constantly update reference libraries, software, and back-up data could ensure these devices are performing to the best of their ability with rapid access to troubleshooting [27]. The simplicity of the C-Vue liquid chromatographer, the automation of the PharmaChk microfluidic system, and the sample variety and accuracy for the QDa mass spectrometer are all desirable traits for an optimal portable confirmatory instrument.

The following devices were selected for field testing described in the field evaluation in Laos (third paper of the PLoS NTD's collection): 4500a, Minilab, MicroPHAZIR RX, NIR-S-G1, PAD, Progeny, and TruScan RM. The main reasons were their ease of use, ease of training, minimal consumables, and ease of export. The RDT were selected as field-suitable but could not be included in the field evaluation because not enough tests were available. The quantitative devices were not selected because of the consumables and training requirements. In addition, the QDa had high mechanical complexity, making export and setup difficult.

Acknowledgments.

We acknowledge invaluable support from Vayouly Vidhamaly, Kem Boutsamay and Phonepasith Boupha in compiling data for data tables. We are very grateful to Bounthaphany Bounxouei, past Director of Mahosot Hospital; to Bounnack Saysanasongkham, Director of Department of Health Care, Ministry of Health; to H.E. Bounkong Syhavong, Minister of Health, Lao PDR; to the staff of the Microbiology Laboratory and LOMWRU for their help and support.

We are very grateful for the support of ADB, especially Dr Susann Roth, Dr Sonalini Khetrapal, Editha S. Santos and ADB Consultant Dr Douglas Ball.

Author Contributions.

SCZ, CC, SV, PNN and FMF wrote the manuscript. MB tested Raman instrumentation and served as blinded analyst for Neospectra data. DVD optimized and tested QDa mass spectrometer. LCW developed methods and prepared simulated medicine tablets. MCB, SCZ, CC, and FMF designed experiments and procured instrumentation. WRG performed Minilab testing.

Supporting information captions.

S1 Appendix. Brief technology description.

S2 Appendix. Notable device configurations and constraints

S3 Appendix. Photographs of the devices.

S4 Appendix. Device descriptions.

S5 Appendix. Device protocols.

S6 Appendix. Field collected medicines tested and UPLC results.

S7 Appendix. Details about simulated medicine preparation.

S8 Appendix. Brief samples handling protocols.

S9 Appendix. Reference library creation questionnaires.

S10 Appendix. US, International, Chinese, And British pharmacopeial standards for the studied APIs.

S11 Appendix. API vs formulation screening.

S12 Appendix. Minilab thin layer chromatography kit results.

S13 Appendix. PAD color analysis

S14 Appendix. Paper Analytical Devices (PAD) results

S15 Appendix. RDT analysis.

S16 Appendix. Rapid Diagnostic Test (RDT) results.

S17 Appendix. 4500a single reflection FTIR spectrometer results

S18 Appendix. Neospectra 2.5 NIR spectrometer results.

S19 Appendix. NIR-G-G1 spectrometer results.

S20 Appendix. MicroPHAZIR RX NIR spectrometer results.

S21 Appendix. Progeny Raman spectrometer results.

S22 Appendix. Intact vs. crushed ACA tablet analysis by Raman and NIR spectrometers.

S23 Appendix. Truscan RM Raman spectrometer results.

S24 Appendix. C-Vue liquid chromatograph results.

S25 Appendix. PharmaChk microfluidic device results.

S26 Appendix. QDa mass spectrometer results.

S27 Appendix. Pair-wise analysis.

S28 Appendix. Table describing the degree of difficulty to analyze other, less common, medicine formulations relative to the analysis of a tablet.

S29 Appendix. Receiver operating characteristic (ROC) curves for the quantitative devices.

S30 Appendix. Limitations of the study

References:

1. World Health Organization. A study on the public health and socioeconomic impact of substandard and falsified medical products. Geneva: World Health Organization; 2017. Available: https://www.who.int/medicines/regulation/ssffc/publications/se-study-sf/en/
2. Nayyar GML, Attaran A, Clark JP, Culzoni MJ, Fernandez FM, Herrington JE, et al. Responding to the pandemic of falsified medicines. Am J Trop Med Hyg. 2015;92: 113–8. doi:10.4269/ajtmh.14-0393
3. Vickers S, Bernier M, Zambrzycki S, Fernandez FM, Newton PN, Caillet C. Field detection devices for screening the quality of medicines: a systematic review. BMJ Glob Heal. 2018;3: e000725. doi:10.1136/bmjgh-2018-000725
4. Roth L, Nalim A, Turesson B, Krech L. Global landscape assessment of screening

technologies for medicine quality assurance: stakeholder perceptions and practices from ten countries. Global Health. 2018;14: 43. doi:10.1186/s12992-018-0360-y

5. Vickers S, Bernier M, Zambrzycki S, Fernandez FM, Newton PN, Caillet C. Field detection devices for screening the quality of medicines: a systematic review. BMJ Glob Heal. 2018;3: e000725. doi:10.1136/bmjgh-2018-000725

6. Weaver AA, Reiser H, Barstis T, Benvenuti M, Ghosh D, Hunckler M, et al. Paper Analytical Devices for Fast Field Screening of Beta Lactam Antibiotics and Antituberculosis Pharmaceuticals. Anal Chem. 2013;85: 6453–6460. doi:10.1021/ac400989p

7. Weaver AA, Lieberman M. Paper Test Cards for Presumptive Testing of Very Low Quality Antimalarial Medications. Am J Trop Med Hyg. 2015;92: 17–23. doi:10.4269/ajtmh.14-0384

8. University of Notre Dame. Paper Analytical Device Project. [cited 30 Aug 2019]. Available: https://padproject.nd.edu/

9. He L, Nan T, Cui Y, Guo S, Zhang W, Zhang R, et al. Development of a colloidal gold-based lateral flow dipstick immunoassay for rapid qualitative and semi-quantitative analysis of artesunate and dihydroartemisinin. Malar J. 2014;13: 127. doi:10.1186/1475-2875-13-127

10. Guo S, Zhang W, He L, Tan G, Min M, Kyaw MP, et al. Rapid evaluation of artesunate quality with a specific monoclonal antibody-based lateral flow dipstick. Anal Bioanal Chem. 2016;408: 6003–6008. doi:10.1007/s00216-016-9363-9

11. Guo S, He L, Tisch DJ, Kazura J, Mharakurwa S, Mahanta J, et al. Pilot testing of dipsticks as point-of-care assays for rapid diagnosis of poor-quality artemisinin drugs in endemic

settings. Trop Med Health. 2016;44: 15. doi:10.1186/s41182-016-0015-8

12. C-Vue Chromatography. [cited 30 Aug 2019]. Available: http://www.cvuechromatography.com/

13. Waters. ACQUITY QDa Mass Detector for Chromatographic Analysis. [cited 30 Aug 2019]. Available: https://www.waters.com/waters/en_US/ACQUITY-QDa-Mass-Detector-for-Chromatographic-Analysis/nav.htm?cid=134761404&locale=en_US

14. Desai D, Zaman MH. Continuous flow microfluidic solution for quantitative analysis of active pharmaceutical ingredient content and kinetic release †. 2015 [cited 30 Aug 2019]. doi:10.1039/c4ay02884h

15. Agilent. 4500 Series Portable FTIR. [cited 30 Aug 2019]. Available: https://www.agilent.com/en/products/ftir/ftir-compact-portable-systems/4500-series-portable-ftir

16. Si-Ware Systems. NeoSpectra-Module. [cited 30 Aug 2019]. Available: https://www.neospectra.com/our-offerings/neospectra-module/

17. ThermoFisher Scientific. microPHAZIR™ RX Analyzer. [cited 30 Aug 2019]. Available: https://www.thermofisher.com/order/catalog/product/MICROPHAZIRRX

18. Wilson BK, Kaur H, Allan EL, Lozama A, Bell D. A New Handheld Device for the Detection of Falsified Medicines: Demonstration on Falsified Artemisinin-Based Therapies from the Field. Am J Trop Med Hyg. 2017;96: 1117–1123. doi:10.4269/ajtmh.16-0904

19. InnoSpectra Corporation. [cited 21 Feb 2020]. Available: http://www.inno-spectra.com/en/product

20. Rigaku. Advanced handheld Raman spectrometer. [cited 30 Aug 2019]. Available: https://www.rigaku.com/en/progeny

21. ThermoFisher Scientific. TruScan^{TM} RM Handheld Raman Analyzer. [cited 30 Aug 2019]. Available: https://www.thermofisher.com/order/catalog/product/TRUSCANRM

22. Global Pharma Health Fund. The GPHF-Minilab^{TM} - Protection Against Counterfeit Medicines. [cited 30 Aug 2019]. Available: https://www.gphf.org/en/minilab/index.htm

23. World Health Organization. Report of the Sixth Meeting of the Member State Mechanism on Substandard and Falsified Medical Products. Geneva; 2018. Available: https://apps.who.int/gb/sf/pdf_files/MSM6/A_MSM6_4-en.pdf

24. Bernier MC, Li F, Musselman B, Newton PN, Fernández FM. Fingerprinting of falsified artemisinin combination therapies via direct analysis in real time coupled to a compact single quadrupole mass spectrometer. Anal Methods. 2016;8: 6616–6624. doi:10.1039/C6AY01418F

25. Caillet C, Sichanh C, Assemat G, Malet-Martino M, Sommet A, Bagheri H, et al. Role of Medicines of Unknown Identity in Adverse Drug Reaction-Related Hospitalizations in Developing Countries: Evidence from a Cross-Sectional Study in a Teaching Hospital in the Lao People's Democratic Republic. Drug Saf. 2017;40: 809–821. doi:10.1007/s40264-017-0544-z

26. Brown LD, Cai TT, DasGupta A. Interval Estimation for a Binomial Proportion. Statistical Science. Institute of Mathematical Statistics; 2001. pp. 101–117. doi:10.2307/2676784

27. Taylor K, Silver L. Smartphone Ownership Is Growing Rapidly Around the World, but Not Always Equally. In: Pew Research Center [Internet]. 2019 [cited 25 Mar 2019] pp. 1–10. Available: https://www.pewglobal.org/2019/02/05/smartphone-ownership-is-growing-rapidly-around-the-world-but-not-always-equally/
