# Supplementary material for: A comparative field evaluation of six medicine quality screening devices in Laos
Source: PLoS Negl Trop Dis. 2021 Sep 30;15(9):e0009674. doi: 10.1371/journal.pntd.0009674 (PMC8483322; doi:10.1371/journal.pntd.0009674)
Supplement: Attachment — Submitted filename: Answer to reviewers - First revision ADB article 3.docx [file pntd.0009674.s016.docx]

Dear reviewers and editors,

We would like to thank you for having carefully commented on our paper, and for giving us the opportunity to improve the manuscript.

Please find below our responses to the comments and questions.

All the corrections are highlighted in yellow in the text of the revised manuscript.

We hope that this revised version will respond satisfactorily to your questions.

Reviewer's Responses to Questions

- Key Review Criteria Required for Acceptance?
- Methods

Reviewer #1: The methods are well applied and the study design was appropriate. However, several general comments regarding the method may be pointed out:

1. The different devices are compared in the same way, which is, in my opinion, a misleading representation of their applicability. Indeed, the devices do not provide the same kind of information and should be used in different situations. For example, the PADs only provide information on the presence (only qualitative and not quantitative information) of a specific API and a few common excipients. This device cannot, and is not intended to (at least to the extent of my knowledge), identify specific brands or detecting substandards. Therefore, I think that the devices should be compared regarding their intended use. This should be clarified and maybe specified for each device.

Thank you for this comment. Indeed, we have not sufficiently stressed this point in the manuscript. We have now emphasized this in the discussion of our revised manuscript (L420-424): ‘Whilst the spectrometers were expected to give information on the dosage formulation of the tested samples, the PADs were designed to tell whether specific API(s) or excipients were present in them, but not to identify samples containing lower than stated amount of the expected API. Hence, caution is needed to interpret the PADs results in comparison with those from the spectrometers.’ And L436-437: ‘Interestingly, although they are not designed to identify samples with API content below specifications, the PAD identified three out of four 50% API samples tested.’

2. In my opinion, the different results regarding the performances should be counterbalance by the advantages and drawback of the different techniques. Indeed, PADs have a poorer capacity at detecting falsified medicines but there is no calibration phase and their price is very low compared to most Raman and NIR devices. This may also be part of the final decision for the choice of a device.

Thank you for this comment. The current article is part of a collection of different publications from a multi-phase study. In other publications, the readers will be able to access our interpretation of the different advantages and drawbacks identified (this was a collaborative project with chemists, public health and economics experts). Notably, the laboratory analysis paper explains the difficulties with spectrometers for the creation of quality reference libraries and the advantages of the PADs. Another article of the series describes and compares upfront and recurring costs of the different devices and provides a cost-effectiveness analysis of the devices’ implementation in the context of Laos. These different aspects are then summarized in a further article submitted to PLOS Medicine in which these advantages/drawbacks are collated to give recommendations on the choice of device and help the readers to make decisions on which device would be the best in their specific settings.

We have added a paragraph in the discussion of the revised manuscript (L492-499): ‘As an independent public health investigation performed in a field setting, this study gives evidence on some aspects of the use of devices in the field to facilitate MRAs decisions as to whether these new technologies are appropriate for screening of diverse medicines in their countries. This article is part of a series of publications describing studies of multiple aspects of the use and implementation of devices in PMS such as their costs, cost-effectiveness, and barriers identified for their implementations (e.g. the difficulties for creating quality reference libraries for spectrometers). These should be considered when making decisions on the best devices to use in specific settings.’

3. An important information is missing regarding the calibration and comparison algorithms used with the spectroscopic devices. This information may possibly be found elsewhere but it is not clear to the reader where to find it. However, this information is crucial when comparing the performances of the devices since a very wide panel of algorithms and calibration strategies may be envisaged. In addition, this calibration phase and the afferent difficulties should be emphasized since it constitutes a major limitation to the use of spectroscopic techniques.

Thank you for this comment. This information is available in the second paper of the series (also referred as the ‘laboratory evaluation’) in which the calibration of the devices were completed, but we omitted this information in the present manuscript. We have now added a short description in the revised manuscript as well as a supplementary file describing these different aspects in more detail.

Indeed, there have been difficulties with the creation of reference libraries for the spectrometers. Those were highlighted in the laboratory evaluation article mentioned above. We have now added this in the revised manuscript L123-125 and more details can be found in Supplementary file **S3 Text – ‘Basic processes for qualitative spectral comparison and protocols for reference library creation’.**

4. Have the statistical models (used to transform the spectral information into a PASS/FAIL result) been validated? If yes, this information together with the validation results should be provided.

As much as possible, we used the default pass/fail thresholds set-up by the manufacturers, with some exceptions as there were differences between devices. We have added a supplementary material (**S3 Text**) and a section ‘Device settings’ in the revised manuscript that explains this procedure (L117-135):

‘Qualitative results obtained with the devices were based upon pattern comparison between a known good quality medicine reference and the test medicine sample data. PAD chemically reacted with the medicine ingredients generating a color pattern that was then visually compared to a reference photograph. The spectrometers computationally compared experimentally-collected spectra to reference spectra of good quality medicines stored in the device’s database. Reference spectra were created for each brand tested in the study and each ‘good quality’ simulated medicine during the laboratory evaluation phase of the project [7]. The protocol for creation of reference spectra is available in **S3 Text**. Each sample spectrum acquired was given a score by the device software resulting from the comparison with the good quality reference library entry. Such scores needed to meet a given threshold to determine if a medicine passed. For the NIR-S-G1 spectrometer, reference samples were sent to the developer who prepared the reference libraries. The passing threshold values for the correlation coefficient or p-value testing initially set as default by the developer in the MicroPHAZIR RX, NIR-S-G1, Progeny, and TruScan RM spectrometers were utilized. These devices would directly tell the user ‘pass’ or ‘fail’, which were recorded. The pass threshold for the 4500a MIR spectrometer’s correlation coefficient was set by us at >0.9 because the device would not output a direct pass/fail result, but rather give a list of matches with their associated correlation coefficients.’

As far as we are aware, these thresholds have not been independently validated for the analysis of specific medicines formulations included in our study. By using default thresholds set-up by the devices’ manufacturers, we believe we reproduced what is likely to occur in settings where there are no expert chemists/chemometricians to validate models.

Reviewer #2: The objectives were clearly articulated and the pilot study design was appropriate. The sample size should have been larger to increase statistical power. Also the authors do not describe sufficiently and clearly how the 'simulated' medicine samples were prepared and verified against their original products especially in term of their packaging and labeling. The study should have included more dosage forms, including tablet, capsule, injectable, suspension, etc. Overall, the study method is quite creative!

Thank you for this comment. Unfortunately, at the time of the study we did not have access to a larger number of falsified field-collected samples. This was stressed in the limitations of the study (**Box 1**).

The simulated medicine samples were loose tablets with no packaging/labelling. They were produced by expert chemists who performed the laboratory evaluation of the devices at the Georgia Institute of Technology, using a pill press as described in the Laboratory paper in this Collection. They were presented loose to the inspectors during the sample set inspections (no simulated medicines were used during the evaluation pharmacy inspections). How the simulated medicine samples were produced is now added in a supplementary material (**S5 Text**). The simulated medicines were tested with devices against reference libraries of simulated medicines containing 100% of the correct API.

Because of the known difficulties of some devices with some dosage forms (e.g. NIR and water-based solutions, spectrometers with capsules shells etc), significantly more development would have been required with each device which was not possible in the timeframe of our study. As this was an exploratory study, we thus decided to only include tablets and powder form of injectable artesunate in this study.

Reviewer #3: Are the objectives of the study clearly articulated with a clear testable hypothesis stated? 5/10

-Is the study design appropriate to address the stated objectives? 5/10

-Is the population clearly described and appropriate for the hypothesis being tested? N/A

-Is the sample size sufficient to ensure adequate power to address the hypothesis being tested? N/A

-Were correct statistical analysis used to support conclusions? 4/10

-Are there concerns about ethical or regulatory requirements being met? N/A

--------------------

Thank you for the scores. We would happily revise the sections that were difficult to read/understand if reviewer n°3 would provide more details on those.

Results

-Does the analysis presented match the analysis plan?

-Are the results clearly and completely presented?

-Are the figures (Tables, Images) of sufficient quality for clarity?

Reviewer #1: _Table 1: The microphazir is not a FT based NIR device but it rather use a Hadamard transform. It is therefore more comparable to the NIR-S-G1 than to a FT instrument (for more information see doi: 10.1177/0003702818809719)

Thank you for finding this error. Indeed, the MicroPhazir RX is dispersive, as is the NIR-S-G1. We have edited this in the revised manuscript (**Table 1**).

_The authors should add a table of abbreviations

We thank reviewer1 for this suggestion to add a table of abbreviations. We would be happy to include it if this is allowed by the journal.

Dear Editor, could you please let us know if we can and in what format to include an abbreviation list.

Reviewer #2: Results were clearly presented with appropriate graphical presentations

Reviewer #3: - Does the analysis presented match the analysis plan? 5/10

-Are the results clearly and completely presented? 7/10

-Are the figures (Tables, Images) of sufficient quality for clarity? 10/10

Thank you for the scores. We would happily revise the sections that were difficult to read/understand if Reviewer N°3 would provide more details on those.

--------------------

Conclusions

-Are the conclusions supported by the data presented?

-Are the limitations of analysis clearly described?

-Do the authors discuss how these data can be helpful to advance our understanding of the topic under study?

-Is public health relevance addressed?

Reviewer #1: _A supplementary limitation that should be listed is the fact that the tested device may not correspond to the actual devices due to software/hardware evolution.

Thank you for this comment. We have added this in the limitations list (**Box 1**): ‘The devices used in this study may have evolved since this study was performed and their performances may have changed. The results of this study may not reflect these changes.’

_Line 144: a risk when analysing tablets through the blisters is the impact of the latter on the final decision. Indeed, from the reviewer experience, a same brand of medicine may be blistered with plastics that exhibit a different spectral signature depending on the batches or providers. In addition, degradation of the blister (e.g. due to exposition to UV light) may distort the spectral signature and lead to wrong conclusion.

Thank you for this remark. For the devices with the ability to scan through the packaging, specific reference libraries were created ‘through the packaging’, using different batches of the same brand of medicine to mitigate the risks of different plastics interference during the study (please refer to the newly added supplementary file: **S3 Text. Basic processes for qualitative spectral comparison and protocols for reference library creation**). However, we did not investigate whether the plastics were different between different batches used.

Samples were always stored in refrigerators away from light. Although this was not investigated in our work, we believe that the time between the creation of the reference libraries ‘through the blister’ and the field study (6 months) was short enough to minimize the risks of wrong conclusion due to degraded plastics.

We have added this in the Limitations box (**Box 1**): ‘We did not investigate whether the plastics of primary packaging were different between batches used for the creation of the reference libraries and the tested samples. Samples were always stored in fridges away from light. Although this was not investigated in our work, we believe that the time between the creation of the reference libraries ‘through the blister’ and the field study (6 months) was brief enough to minimize the risks of wrong conclusions due to degraded plastics.’

_Table 2: In my opinion, it is risky to use laboratory-made tablets to calibrate a NIR device and use this calibration set to analyse industrially manufactured tablets. The differences in excipient origin/particle size but also the tablet shape, force of compression etc. will have an impact on the spectrum. This may lead to over optimistic results because the detection of the “falsified” or “substandard” tablet may be linked to these differences rather on the API strength. This kind of information should be present in the validation of the methods.

We did not use calibration sets made with laboratory-made tablets to analyse industrially manufactured tablets in our study. ‘Laboratory-made tablets‘ of various quality (containing only excipients, or reduced %API) were tested against ‘good quality laboratory-made tablets’ (containing the expected API and various excipients) references. Industrially manufactured tablets were tested against good quality manufactured tablets (exactly same brand & formulation, but different batch).

We have added information on how the ‘simulated medicines’ were produced in **S5 Text** and how the reference library entries were developed and used to analyse samples in **S3 Text**.

_The authors should discuss the performances of the techniques regarding mono vs multi component formulations. Indeed, it is very hard for spectroscopic techniques to detect the absence of artemether in a lumefantrine/artemether formulation whether it is easier for colorimetric techniques.

We agree in the importance of this and discuss this in our background review (Vickers et al. 2019). We would have liked to investigate this topic using experiments with co-formulated APIs versus the APIs alone, but this was beyond the capacity of the project.

Reviewer #2: The conclusions drawn from this pilot study emphasize the need for further investigations and studies in real life settings and proper validation of the technologies prior to their deployment for post-market surveillance by the regulatory agencies. The limitations of this pilot study are clearly explained. The study is very relevant to the current public health issues on substandard and falsified medicines that need to be addressed.

Thank you very much for this comment.

Reviewer #3: Are the conclusions supported by the data presented? 2/10

-Are the limitations of analysis clearly described? 8/10

-Do the authors discuss how these data can be helpful to advance our understanding of the topic under study? 8/10

-Is public health relevance addressed? 6/10

--------------------

Thank you for the scores. We would happily revise the sections that were difficult to read/understand if Reviewer N°3 would provide more details on those.

Editorial and Data Presentation Modifications?

Use this section for editorial suggestions as well as relatively minor modifications of existing data that would enhance clarity. If the only modifications needed are minor and/or editorial, you may wish to recommend “Minor Revision” or “Accept”.

Reviewer #1: _Line 34: please specify “near infrared” for the Phazir and NIR-S-G1 to avoid confusion with the “mid infrared “ 4500a device at line 35.

This has been added in the revised manuscript (**L34-35**).

Reviewer #2: No

Reviewer #3: Please see comments to the authors

--------------------

Summary and General Comments

Use this section to provide overall comments, discuss strengths/weaknesses of the study, novelty, significance, general execution and scholarship. You may also include additional comments for the author, including concerns about dual publication, research ethics, or publication ethics. If requesting major revision, please articulate the new experiments that are needed.

Reviewer #1: The paper submitted by Caillet et al. describes the comparison of six devices to screen the quality of medicines. The paper is well written and easy to read despite the huge amount of data and results. The present paper is one of the first papers to try to have an objective evaluation and comparison of screening devices in real (or almost realistic) conditions. This should be emphasized and was well appreciated by the reviewer.

The paper deserves publication in PLOS NTD since it provides very useful information regarding the acceptability of these techniques by field inspectors. This kind of information is crucial when developing new devices or methods to ensure their correct application and acceptance by end-users. Another interesting finding is the impact that the use of screening devices has on the usual visual inspection leading to potentially sloppy inspection. Indeed, the screening devices should be considered as a supplementary tool to help inspectors detecting falsifications but not as the ultimate solution.

Thank you very much.

Reviewer #2: (No Response)

Reviewer #3: The objective of this original work is to evaluate the usefulness and ease of use of six portable screening devices in the hands of Lao medical inspectors for an inspection in a simulated evaluation pharmacy.

This is a topical issue in the fight against the use of counterfeit drugs in non-industrialized countries that affect both human and animal health.

The text is well written, the summary tables are clear and even the limitations of the study are extremely well presented.

No worries on the form.

I wonder about the options chosen by the authors to clarify, to promote decision support for the use of such or such control devices.

The current article does not cover all different aspects that impact the decision as to what device is the best in specific settings. We have submitted another article (under revision) to PLOS Medicine that summarizes the results of the articles in this Collection that includes the laboratory evaluation, the current study, and a cost-effectiveness analysis of the devices. It describes recommendations on how best to choose device(s) for implementation in specific settings. We have added this in the discussion (**L495-499**): ‘This article is part of a series of articles describing studies of multiple aspects of the use and implementation of devices in PMS such as their costs, cost-effectiveness and the barriers identified for their implementations (e.g. the difficulties for creating quality reference libraries for spectrometers), that should be taken into account when making decision on the best devices to use in specific settings.’

First, I would like to come back to the two objectives: usefulness and ease of use

Could we define the term usefulness. because if I understood correctly the objective is still to discern "the true" from the "false" for drugs in terms of sensitivity specificity and/or false positive, etc. .....

The aim of this study was to evaluate the ‘usability’ and the ‘utility’ of the devices. The usability of a device includes three aspects (ISO 9241-11:2017, *Ergonomics of human-system interaction — Part 11: Usability: Definitions and concepts.* <https://www.iso.org/obp/ui/#iso:std:iso:9241:-11:ed-2:v1:en>). First, the effectiveness is the ability of users to complete tasks using the system and the quality of the output of those tasks. Second, is the efficiency or level of resource consumed in performing tasks. Third, the satisfaction is the users’ subjective reactions to using the system. Utility of the devices for implementation in post-market surveillance was assessed through the satisfaction questionnaire and during focus group discussions.

**Effectiveness** was measured in our study by:

- The extent to which the protocol for device use was followed by the inspectors. This was determined by the real time observation of device use in the evaluation pharmacy and sample set testing. Mistakes were recorded by the observer and the stored data in the device was reviewed when available.
- The number of samples wrongly categorised (when the inspector’s final decision about sample quality differed from the UPLC result). Wrong categorisation can be due to error(s) at any point in the process of testing including: preparation of the sample and device prior to testing, during device analysis, user reading of the result, and user interpretation of the result.

The final result for the sample is the sum of the previous steps.

The overall effectiveness of the inspection is a combination of the inspector’s ability to correctly use the device and the device’s ability to deliver the correct test result (‘correct’ where the result returned by the device is the same as that given by UPLC, the current gold standard test).

**Efficiency** was measured in our study by assessing the level of resource (primarily time) consumed by the device in performing the desired task.

To summarize, are we evaluating the "machine" or the "assistant" or both? If I understand correctly, the last option is retained.

Indeed, we are evaluating both.

Then the question arises of the assistants and more exactly of their training in terms of calibration (intra, extra examiner variability) as recommended by the WHO. It is impossible to evaluate the performance of the devices if one biases from the beginning by not placing oneself in correct human resources conditions. Unless you place yourself in a pragmatic and not explanatory logic, which is not the case of your study.

Thank you for this comment. We did not primarily aim to evaluate the performances of the devices themselves. This was a separate part of our multi-phase project by expert chemists at the Georgia Institute of Technology. It is the second article published in the PLOS NTD’s series, currently under revision.

We are not aware of any recommendations from WHO on the training needed for the use of devices. This is sorely needed.

The statistical analysis can be improved. We have a "machine" effect, an "examiner" effect (which varies in intra and inter) and a "Machine "X "Examiner" dependent effect.

Thanks for your suggestion. Indeed, an interaction gives useful information as to whether the performances of the devices would depend on the inspector. The interaction test would indeed be the best in that case to tease out such information. However, with the nature of our study design and the nature of the data, an interaction test does not have a good practical meaning. The reason is that there were many inspectors (categorical data) and many devices (categorical data). This results in a factorial of linear combinations when fitting an interaction tests model and hence the model becomes overparameterized and not efficient. In addition, there are few data points in each of the interaction cells, hence a lot of uncertainty.

Addressing this by considering inspector as a clustering variable seemed to be the most statistically efficient approach that we took, while we agree that if when had fewer categories of devices and inspectors, we would have performed a model that contains the interaction terms between device and inspectors.

By considering the inspector as a clustering variable, it captures the intracluster correlation, i.e. the ratio of variance in outcomes between inspectors to the total variance in the outcomes (variance in outcomes between inspectors plus variance in outcomes within inspectors).

We have added this limitation in the Box 1: ‘A statistical interaction between ‘device’ and ‘inspector’ was not included in our statistical analyses. This would have led to overparameterization of the models. We included inspectors as cluster-specific random effect.’

You thus approach in a very precise way the time spent by acts which is not in itself on a priority evaluation grid (absent in the material and methods).

We are not aware of the use of priority evaluation grids for the evaluation of medicine quality screening devices. We agree that this would be a useful approach for future work.

In the same way, approaching the perception or satisfaction of users in a "QALY study" manner is not logical. Here again, it is necessary to prepare a robust evaluation questionnaire beforehand.

The satisfaction of users and their perceived utility was assessed qualitatively after the inspections using a questionnaire and during focus group discussions. As far as we are aware, our study is the first investigating this aspect and we are not aware of any prior robust questionnaire to assess this. Our study was an exploratory study. We believe that further developing and validating a questionnaire to assess the satisfaction of users of screening technologies should be the aim of a study on its own.

Concerning the discussion and the conclusion, if I am a public health decision-maker, I remain uncertain.

The logic, like any comparative trial, would be to prioritize the products or group of products and produce operational recommendations. Otherwise, we miss our target.

As explained above, this article is part of a larger project that covers different aspects of the implementation of screening technologies, that we believe are important for producing informed-based recommendations. A paper submitted to PLOS Medicine (currently under revision) describes the advantages/drawbacks of the devices identified in the 3 different studies of our multi-phase project as well as a 2-days multi-stakeholders meeting with regulators from 7 countries (mainly South-East Asian) and representatives of other organizations (including WHO, UNDP, USP). The article provides recommendations on the choice of device in their specific settings.

My advice, but it is only advice, would be to restructure your article. The priority would be: What are in terms of sensitivity, specification, etc... the best or worst devices. And this, by placing you in a pragmatic logic, ie, a standard level of training, "basic" by describing the nature of training in the form of figure or table of synthesis. And therefore take out the criteria "time" and "perception" of the analysis to arrive at objective "machine" recommendations more or less hierarchical. The time factor could then be a secondary outcome or... a second article

We discuss the integration of the laboratory, Lao and economics aspects of this study in the final paper of this Collection. Such hierarchical classification is complicated and will vary depending on the settings where the devices will be used, the medicines being tested and the context, such as whether the prevalence of falsified is much higher than that of substandard medicines, and where the devices will be used within the pharmaceutical supply chain will affect the decision-makers’ choice.

We describe in great details the different aspects decision-makers should consider before implementing devices in PMS in the Recommendations paper submitted to PLOS Medicine.

--------------------

PLOS authors have the option to publish the peer review history of their article (<a href="https://journals.plos.org/plosntds/s/editorial-and-peer-review-process#loc-peer-review-history" target="_blank">what does this mean?</a>). If published, this will include your full peer review and any attached files.<br><br>

If you choose “no”, your identity will remain anonymous but your review may still be made public.<br><br>

Do you want your identity to be public for this peer review?</b> For information about this choice, including consent withdrawal, please see our <a href="https://www.plos.org/privacy-policy" target="_blank">Privacy Policy</a>.

Reviewer #1: No

Reviewer #2: Yes: Souly Phanouvong

Reviewer #3: No

**Further edits requested by Reviewer 2**

P4. Consider updating the name of the FDQCC. Since September 2019, the center new name is 'National Center for Food and Drug Analysis' or NCFDA’

Thank you. This was changed in the revised manuscript (**L92-93**).

L117-118: pls clarify whether or not Vientiane BFDI is the Vientiane municipality BFDI or it is the 'central level BFDI' based in Vientiane Capital City or it is the Vientiane Province BFDI?

Thank you for this comment. We have changed in the revised manuscript (**L137-138**): ‘Sixteen medicine inspectors, employees of the Vientiane Central Bureau for Food and Drug Inspection based in Vientiane Capital (BFDI, n=10) and the Vientiane District BFDI offices (n=6), volunteered for the study.’

L121-122: would be helpful to readers if authors specify number days or hours the training took

We have added this in the methods section of the revised manuscript (**L139-145**). ‘They were randomised to receive either a ‘rudimentary’ verbal training with opportunity to practice use of the device on a few practice samples just prior the EP inspection (5-10 min); or an ‘intensive’ training including verbal presentation and substantial practice with the device (1h-2 h), plus an additional rudimentary verbal training and practice just prior to the EP inspection.’

L130-131: on how many medicine samples?

For all the evaluation pharmacies inspections, the inspectors were free to test as many medicines as they wanted. We have specified this further in the revised manuscript (**L160-165**): ‘All inspections were carried out independently by the participant working alone with one device per inspection. Inspectors were asked to collect any suspicious samples containing the targeted API assuming: no time limit (hence the collectors were free to inspect as many samples as they wished to), no budget restriction, that it was June 2015 (to avoid bias because some of the medicines in the EP had already expired by the evaluation) and that all blisters had no tablets missing (as some tablets were removed for analysis).’

L144-146: pls elaborate a bit more whether or not the unpacked samples were the same lot/batch number of the same product that were stored at the same conditions.

Thank you. We have added this precision in the revised manuscript (**L166-169**): ‘However, if they wished to perform testing requiring opening of primary packaging, the observer provided inspectors with already unpackaged samples (in a small zip-lock bag) of the same batch number of the product that were stored at the same conditions.’

L174-175: this sentence is not clear. As is, sounds like samples were FDC containing 2 APIs or 3 APIs.

Thank you. We hope it is now clearer in the revised manuscript (**L198-199**): ‘Each technician was assigned to the testing of all samples of two or three API (e.g. inspector A tested all the samples of SMTM and AL).
